# Refining bulk segregant analyses: ontology-mediated discovery of flowering time genes in *Brassica oleracea*

**DOI:** 10.1186/s13007-022-00921-y

**Published:** 2022-07-04

**Authors:** Rutger A. Vos, Catharina A. M. van der Veen-van Wijk, M. Eric Schranz, Klaas Vrieling, Peter G. L. Klinkhamer, Frederic Lens

**Affiliations:** 1 Naturalis Biodiversity Center, P.O. Box 9517, 2300 RA Leiden, The Netherlands; 2 Institute of Biology Leiden, Leiden University, Sylviusweg 72, 2333 BE Leiden, The Netherlands; 3 Biosystematics Group, Wageningen University and Research, P.O. Box 16, 6700AP Wageningen, The Netherlands

**Keywords:** Bulk segregant analysis, Quantitative trait locus, Gene Ontology, Pathway analysis, Enrichment analysis, SNP effects

## Abstract

**Background:**

Bulk segregant analysis (BSA) can help identify quantitative trait loci (QTLs), but this may result in substantial bycatch of functionally irrelevant genes.

**Results:**

Here we develop a Gene Ontology-mediated approach to zoom in on specific genes located inside QTLs identified by BSA as implicated in a continuous trait. We apply this to a novel experimental system: flowering time in the giant woody Jersey kale, which we phenotyped in four bulks of flowering onset. Our inferred QTLs yielded tens of thousands of candidate genes. We reduced this by two orders of magnitude by focusing on genes annotated with terms contained within relevant subgraphs of the Gene Ontology. A pathway enrichment test then led to the circadian rhythm pathway. The genes that enriched this pathway are attested from previous research as regulating flowering time. Within that pathway, the genes *CCA1*, *FT*, and *TSF* were identified as having functionally significant variation compared to *Arabidopsis*. We validated and confirmed our ontology-mediated results through genome sequencing and homology-based SNP analysis. However, our ontology-mediated approach produced additional genes of putative importance, showing that the approach aids in exploration and discovery.

**Conclusions:**

Our method is potentially applicable to the study of other complex traits and we therefore make our workflows available as open-source code and a reusable Docker container.

**Supplementary Information:**

The online version contains supplementary material available at 10.1186/s13007-022-00921-y.

## Background

Identifying the genes that underlie quantitative trait variation is one of the main challenges in genetics and, to the extent that this is attainable in silico, in bioinformatics. One appealingly straightforward approach to discovering candidate loci involved in quantitative trait differences is to sort individuals of a segregating, crossed population into pools defined by extremes in trait values and then interrogating the genetic contrasts between these pools, i.e. bulk segregant analysis (BSA [1, 2]). High-throughput sequencing of DNA in pools has made it possible to quickly generate haystacks of data at low cost, within which are the genetic needles (genomic regions, specific genes, and finally SNPs) that caused the salient differences between the pools.

Several statistics have been developed to aid in the discovery of candidates of these needles. For each SNP in a sequenced pool, metrics exist that express its relative coverage compared to other pools (the Δ(SNP-index) Sensu Takagi et al. [3]) or whether its allele frequency deviates from the expectation (the modified Gʹ statistic of Magwene et al. [4]). Then, having defined a threshold value for the metric and using a sliding window approach, regions of (more or less) contiguous SNPs in whose metric values the pools differ can be found, resulting in putative quantitative trait loci (QTLs) in the form of genomic regions. If the analysis is performed using a sufficiently annotated reference genome to map SNPs to genes, SNPs that regulate the trait and intersect with the intervals can be directly pinpointed. However, this general approach is somewhat imprecise (and more so with low thresholds or large window sizes), resulting in a lot of ‘bycatch’ of irrelevant genes. Here, we present an approach to remove such bycatch and obtain more refined result sets by traversing and pruning subgraphs of Gene Ontology [5] annotations and KEGG pathways [6] enriched by the initial QTL finding.

We apply and validate this approach using pools determined by contrasting flowering time in a *Brassica oleracea* cross. The remarkable variation in *B. oleracea* in flowering time is a critical agronomic trait. For example, whereas broccoli is a short-lived annual that flowers in the year it was planted, cabbage is biannual, needing a cold period to induce flowering (i.e. vernalization) [7]. Research in *Brassica* has been advanced by the release of genomes from various species (e.g. [8–10]), including two reference genomes from *B. oleracea*: the rapid cycling line TO1000DH3 [11] and *B. oleracea* var. *capitata* [12]. The genetics that underlies variation in flowering time within and among *Brassica* species is reasonably well characterized [13]. The *FLOWERING LOCUS T* (*FT*) locus, its transcriptional repressor *FLOWERING LOCUS C* (*FLC*), and its transcriptional activator *CONSTANS* (*CO*) all play a central role both in *B. oleracea*, *B. rapa* and in *Arabidopsis*. However, the way *FT* expression is modulated differs between *Brassica* and *Arabidopsis*. The overall flowering time pathway is much more complex in all cases, involving over two dozen other genes in multiple, divergent copies scattered across the genome [13]. As the exact locations of these copies are mostly known, sufficient background information is available to validate and interpret the results of the analysis we present here and assess its potential for applicability in less well-characterized traits. To be specific, with this background information we demonstrate that our approach both recovers the precise genes involved in regulating flowering time in other kale cultivars as well as other, plausible candidate genes. As such, our novel approach may help tackle issues of candidate gene prioritization.

**Fig. 1:**
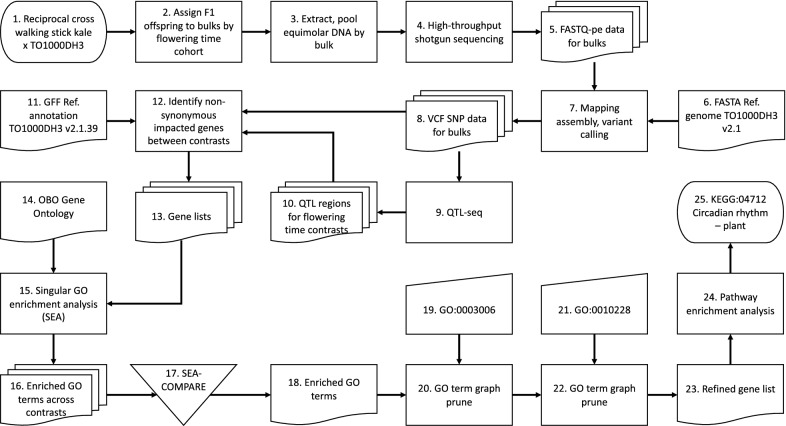
Flowchart of the main workflow. This flowchart omits the separate genome sequencing of the Jersey kale accession and subsequent analysis thereof. In the Methods section, we refer to the panels in this flowchart by the number prefixes shown here to orient the reader on the procedure

The workflow we present here is shown in Fig. 1; we reference the constituent steps in the Methods section. As we demonstrate, this workflow helps discover and filter candidate genes from BSA QTLs in our model system, i.e. flowering time in certain *B. oleracea* cultivars. This system is a useful test of the approach, as the regulation of flowering time in other cultivars is fairly well characterized [13], which helps verify our results. However, this previously published characterization of flowering time can also be applied directly to additional cultivars, which has the advantage that genetic variation in the same set of homologs and paralogs can be interrogated—with the drawback that no novel loci will be discovered. Nevertheless, we also present this approach here, because the outcomes were so complementary with the BSA. We sequenced the novel genome of the late-flowering, heterozygous, giant woody walking stick kale native to Jersey Island (cultivar *B. oleracea* convar. *acephala* var. *viridis*), one of the two parents of the BSA crosses (the other being the rapid cycling, homozygous line TO1000DH3, which has been sequenced before [11]). For this Jersey kale genome, we assessed the impact of SNPs within known flowering time pathway genes [13] and compared and contrasted these with the genes discovered through our BSA analysis. The substantial finding that complements the BSA results is that high-impact SNPs (i.e. those where the gene is inactivated due to lost start or gained stop codons) occur in paralogs outside of the QTLs we recovered, while moderate-impact SNPs (i.e. with non-synonymous substitutions) fall within the QTLs. Hence, the combination of the approaches allows us to infer that flowering time in our crosses is regulated by the additive effect of non-synonymous substitutions and not, for example, through pseudogenation.

## Methods

### Plant material, crosses, genotyping and phenotyping

We crossed the homozygous doubled haploid *B. oleracea* kale-like alboglabra line TO1000DH3 [11] with the giant woody walking stick kale (*B. oleracea* convar. *acephala* var. *viridis*) native to Jersey (Channel Islands, UK [14, 15]), the latter grown from seeds ordered from Mr and Mrs Johnson, who own a company making artisanal walking sticks (Homestill, La Grande Route de St. Jean, St. Helier, Jersey, Channel Islands). We selected TO1000DH3 for its rapid flowering time and short generation time (approx. 65 days). In contrast, the Jersey kale is extremely late flowering, has a much longer generation time (at least 6 months), and requires a vernalization period. We crossed the two parents reciprocally, resulting in F1 seeds from both parents, which we established in tissue culture and potted in soil (Fig. 1, panel 1).

**Fig. 2:**
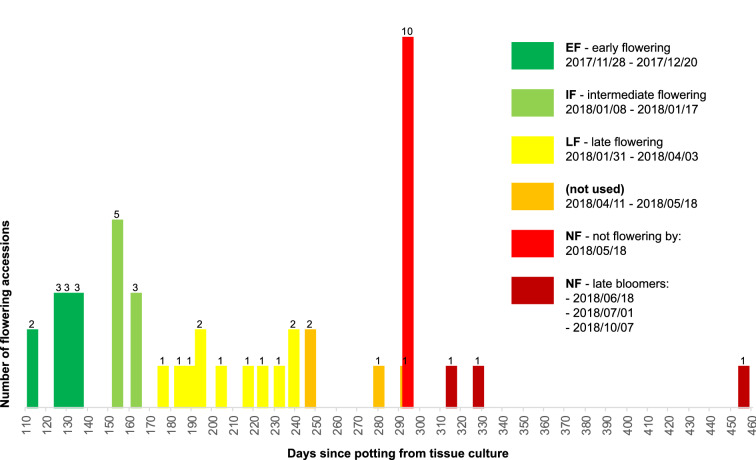
Phenotyping results and assignment to pools. F1 individuals were assigned to one of four pools: Early Flowering (EF), Intermediate Flowering (IF), Late Flowering (LF) or Non-Flowering (NF). Of the latter, three individuals (late bloomers) flowered anyway, after DNA extraction. Four accessions were not used in order to create a greater contrast between late and non-flowering phenotypes

We genotyped the F1 population with an allele-specific assay (KASP) on our in-house high throughput SNP genotyping platform and phenotyped the plants on time till first flowering based on two individuals per genotype, distinguishing early (EF), intermediate (IF), late (LF) and non-flowering (NF, at time of DNA extraction) cohorts (Fig. 1, panel 2). We set the boundaries between these different cohorts such that we obtained pools of roughly equal numbers of individuals (around ten per pool; Fig. 2) and increased phenotypic contrast between late and non-flowering accessions by skipping four especially late flowering individuals in the LF pool.

### DNA extraction and sequencing data pre-processing

We performed genomic DNA extractions on a KingFisher Flex magnetic particle processor robot (Thermo Scientific) using a NucleoMag^®^ 96 Plant kit (Macherey–Nagel GmbH & Co.). We used a volume of 150 μl for elution. We measured DNA concentrations on a Dropsense (TRINEAN NV) using a DropPlate 96-S. Based on these measurements, we pooled the DNAs of the same phenotype (EF, IF, LF and NF) equimolarly to create four DNA pools for sequencing (Fig. 1, panel 3). We prepared libraries according to the protocol of Macrogen, containing random fragmentation of the DNA sample followed by 5ʹ and 3ʹ adapter ligation, amplification of the adapter-ligated fragments using unique index primers and gel purification. From this, we sent 400 ng DNA aliquot to Macrogen for paired-end sequencing on the Illumina HiSeq X platform (read length 150 bp) on a shared run (Fig. 1, panel 4).

We used the BWA-MEM [16] and SAMtools [17] toolchain to map (Fig. 1, panel 7) each pool’s reads (Fig. 1, panel 5) against the *B. oleracea* TO1000DH3 reference genome v2.1 of EnsemblPlants release 39 (Fig. 1, panel 6), which we filtered so that we mapped against chromosomes only. We then used GATK HaplotypeCaller [18, 19] for variant (i.e. SNP and indel) calling, yielding the results summarized in Table 1.

**Table 1:** Summary results of the sequencing of pools of early (EF), intermediate (IF), late (LF) and “non” flowering (NF, actually not flowering at time of DNA extraction) phenotypes

Phenotype	Pool size	Total read bases (bp)	Total reads	GC (%)	Q20 (%)	Q30 (%)	Coverage a, b	Variants
*EF*	11	60,303,831,724	399,363,124	36.93	95.02	89.25	123, 108	40,224,519
*IF*	8	56,804,209,216	376,186,816	36.92	94.84	88.94	116, 103	43,785,856
*LF*	11	54,587,863,530	361,509,030	37.14	96.00	91.34	112, 100	42,852,937
*NF*	9	54,296,890,456	359,582,056	37.13	96.84	92.81	111, 99	42,213,427

Pool size refers to the number of individuals pooled for that phenotype. Coverage is given as (a) total read bases divided by reference genome size; and (b) average mapped coverage

### Pool genotyping and QTL region analysis

Given that we phenotyped the F1s by flowering time binned in four pools, there are six pairs of contrasts (i.e. EF ↔ IF, EF ↔ LF, EF ↔ NF; IF ↔ LF, IF ↔ NF; and LF ↔ NF). We performed joint genotyping for these contrasts using the GATK CombineGVCFs/GenotypeGVCFs workflow. We then filtered these genotypes further, excluding low coverage per sample (< 40×), low coverage across the pair of merged samples (< 100x), unusually high coverage (> 400×, e.g. repeats), low values for the GATK Genotype Quality score (< 99), and low values for the frequency of the reference allele (< 0.2, a conservative value as TO1000DH3 is homozygous). We calculated smoothed G statistics (G×ʹ, see [4]) over a sliding window 1 Mb wide, filtering outliers by Δ(SNP-index) [3] and retaining all SNPs with Gʹ > 2.5 for further analysis (Fig. 1, panel 8).

**Fig. 3:**
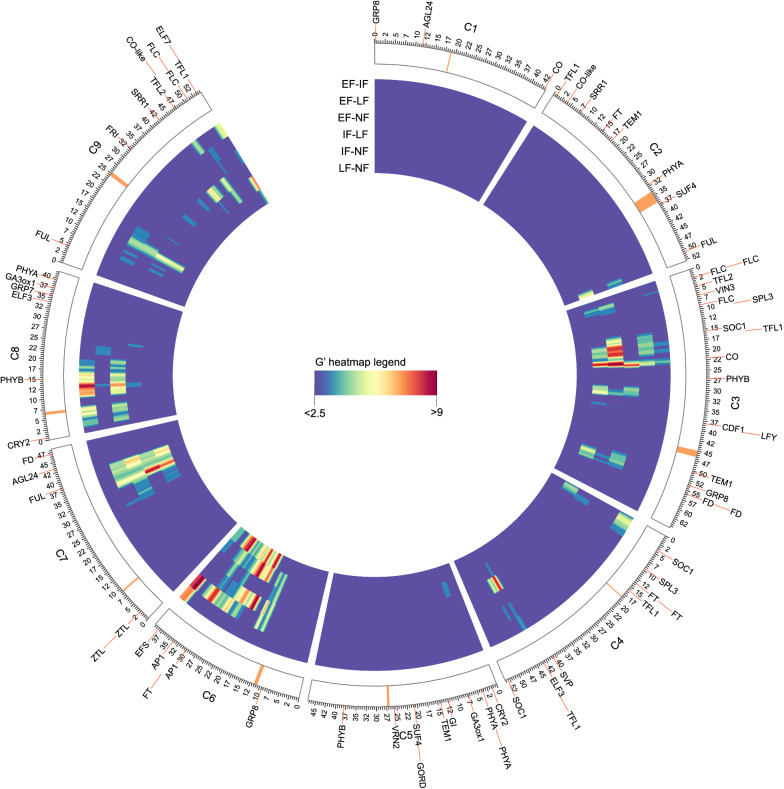
‘Circos’ plot of QTL regions. Six concentric heatmaps show QTLs identified for the six possible contrasts among the four pools. The QTLs are mapped on the annotated TO1000DH3 reference, showing the locations of flowering time genes previously identified for *B. oleracea* [13]. Centromeres indicated in orange; units of chromosomal locations in megabases

We then performed a QTL-seq analysis [3] to identify candidate QTL regions by simulation using 10 k replicates and a two-sided 95% confidence interval (Fig. 1, panel 9 and Fig. 3). For the Gʹ and QTL-seq calculations and simulations, we used the R package QTLseqr [20]. Based on our inferred QTL regions (Fig. 1, panel 10) and smoothed Gʹ values, we scanned the mapped assembly of each pool for genes that fall within QTL regions and have non-synonymous SNPs with high Gʹ (Fig. 1, panel 11). Gene coordinates were based on the annotation of the TO1000DH3 (i.e. the *B. oleracea* GFF3 release v2.1.39 of EnsemblPlants; [11], Fig. 1, panel 12). To cross-reference the products of these genes with other information resources, we then mapped the *B. oleracea* genes to the curated and machine-predicted proteomics identifiers of UniProtKB/TrEMBL [21] using BioMart [22] (Fig. 1, panel 13).

### Functional enrichment, ontology-mediated refinement, and pathway analysis

We performed singular enrichment analyses (SEA, [23], Fig. 1, panel 14) separately for all six contrasts using the agriGO web service [24], which uses the Blast2GO [25] results for *B. oleracea* compiled by the Blast2GO Functional Annotation Repository (B2G-FAR, [26]) to establish a reference list (Fig. 1, panel 15) against which to assess term enrichment by way of a hypergeometric test corrected for multiple comparisons using the Benjamini–Yekutieli method [27]. To determine the overlap between our SEAs, we merged their results in a cross-comparison (SEACOMPARE, [24], Fig. 1, panel 17), which showed congruence in enriching numerous terms related to reproduction across all contrasts (Fig. 1, panel 18). For each of the SEA result sets, we pruned the enriched (FDR < 0.05) subgraph (Fig. 1, panel 19) by retaining only those terms that are reproductive developmental processes, i.e. that are subtended by the upper-level term *developmental process involved in reproduction* (GO:0,003,006, Fig. 1, panel 20) from the domain *biological process* of the Gene Ontology [5]. Within the pruned subgraph (Fig. 3), three out of the top-level terms are related to flower development or morphogenesis, one to seed maturation, and one (GO:0,010,228, Fig. 1, panel 21) is defined as:

*“The process involved in transforming a meristem that produces vegetative structures, such as leaves, into a meristem that produces reproductive structures, such as a flower or an inflorescence.”*

As this developmental process precedes those defined by the other top-level terms in the subgraph, we took (Fig. 1, panel 22) the ontology-mediated list of genes (Fig. 1, panel 23) annotated to these terms. We used this as the input for a pathway enrichment analysis (Fig. 1, panel 24) as implemented in g:Profiler [28]. This yielded an alternative view in the extent to which the genes enrich other GO terms, as well as any KEGG [6] pathways (Fig. 1, panel 25).

### Genome analysis of the Jersey kale

To gain more background insight into the genome of the giant woody Jersey kale as a potential model in general, and with an eye on differences with TO1000DH3 in flowering time loci in particular, we also sequenced the genome of a specimen of this cultivar. We followed the same protocols for DNA extraction, sequencing, genome assembly, and variant calling described for the pools in the section DNA extraction and sequencing data pre-processing. However, as there was no pooling of multiple individuals (i.e. no BSA), the coverage for this single individual was commensurately higher (approx. 100 × coverage). For this Jersey kale genome, we then used SnpEff [29] to assess the impact of SNPs within the loci previously identified as participating in the flowering time pathway of *Arabidopsis* [13]. The methods are described in greater detail in Additional file 6.

## Results

The F1 seeds from our crosses resulted in 42 distinct genotypes that we successfully established in tissue culture and transferred in soil. We found a 420-day time lag between the earliest and the latest flowering F1 genotypes, presumably owing to the heterozygosity of the Jersey kale parent: the first two F1 genotypes (genotype numbers 17,135, 17,136) started to flower 113 days after potting, while more than a year later, at day 533, the last F1 flowered (genotype number 17109). The first pool, early flowering EF, comprised 11 F1 genotypes that flowered between 113 and 135 days after potting. The second pool, flowering at intermediate age IF, included eight F1s that flowered from 154–164 days after potting. The third pool, late-flowering LF, represented 11 F1 genotypes that started to flower from 176–239 days after potting. The fourth pool, non-flowering (NF) at the time of DNA extraction (day 294), included 9 F1s that only flowered after DNA extraction, up to 533 days after potting. Phenotyping results are summarized in Fig. 2 and detailed in Additional file 1: Table S1.

Sequencing resulted in yields per pool between 54.3 × 10^9^ and 60.3 × 10^9^ bases, observed in 359.6 × 10^6^ through 399.4 × 10^6^ reads (which are 150 bp on HiSeq X). Given the size of the reference genome (approx. 488 Mbp [11]), this corresponds to a depth in the range of 111×–123 × per pool, or about 10 × per individual. We retained most of the estimated raw coverage in the assemblies, yielding an average mapped coverage of 99×–108×(more sequencing statistics are listed in Additional file 2: Table S2). Following variant calling and joint genotyping of the six pairwise comparisons of phenotype pools, our Gʹ sliding window analysis produced fairly consistent results across all comparisons. We found regions with windows of Gʹ > 2.5 on all chromosomes but C1. Still, regions that featured in the majority of pairwise comparisons were restricted to the q arm of C3 (spanning, for example, the locus of one of the CO copies), C6 (spanning one of the *FT* loci), C7 (spanning a *FUL* copy) and C8 (spanning a *PHYB* copy). A comparative view of these Gʹ regions is shown in Fig. 3. More detailed views of the regions are provided in the supplementary materials on Zenodo: 10.5281/zenodo.3402201 (the gprime.png files in the contrasts.zip archive), including the Gʹ null. At this stage of the analysis, the QTL regions intersected with the genomic coordinates of 14,257 genes, out of which 10,469 had non-synonymous SNPs.

The term enrichment analyses (SEA, [23]) yielded between 812 and 1409 significantly enriched terms (under FDR correction) for the six pool contrasts. Interestingly, the number of terms appears to covary somewhat with the magnitude of the trait differences, in that contrasts between the contiguous cohorts EF ↔ IF and LF ↔ NF enriched the lowest numbers of terms (1086 and 812, respectively) while those between the non-contiguous cohorts IF ↔ NF and EF ↔ NF returned the most terms (1409 and 1280). Nevertheless, the different analyses’ results overlapped extensively, as the total number of distinct terms returned overall was 1544. This was confirmed by SEACOMPARE ([24]), which also indicated extensive overlap across the six comparisons (shown in Additional file 3: Table S3).

**Fig. 4:**
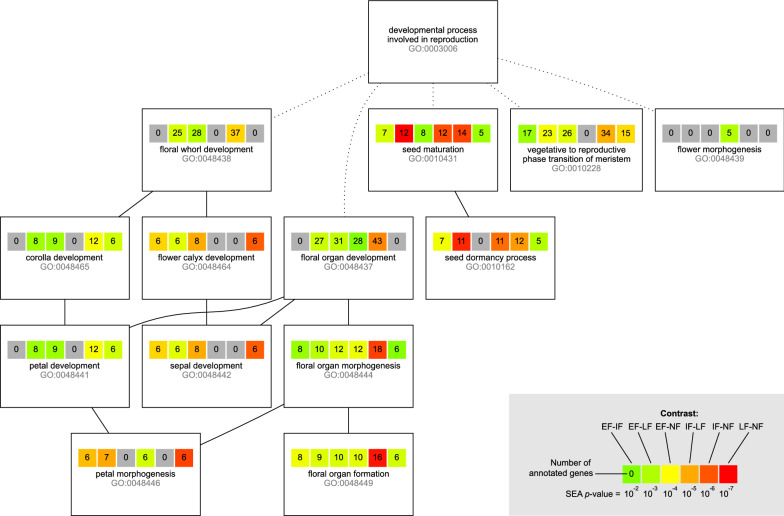
Genes annotated to GO subgraph terms. Each box represents a GO term subtended by GO:0003006. Within each box, the six pool contrasts for four pools (i.e. EF ↔ IF, EF ↔ LF, EF ↔ NF; IF ↔ LF, IF ↔ NF; and LF ↔ NF) are shown as squares. Each square shows the number of genes annotated with that term for that contrast. The color coding corresponds with the significance of the enrichment as an inverse heatmap, i.e. the smaller *p*, the hotter the tint

Each of the comparisons enriched a subgraph of the GO topology, which we pruned further to retain only those parts subtended by GO:0003006 (*developmental process involved in reproduction*). Across the comparisons, this resulted in a consensus subgraph that spanned 14 enriched terms, shown in Fig. 4. Among these 14 terms were five upper-level terms that have an implied ordering in time (e.g. seed maturation necessarily follows floral development) and specificity (e.g. the floral whorl is part of the floral organs) with respect to their contribution to the onset and further development of flowering:

1. GO:0010228—*Vegetative to reproductive phase transition of meristem*
2. GO:0048439—*Flower morphogenesis*
3. GO:0048437—*Floral organ development*
4. GO:0048438—*Floral whorl development*
5. GO:0010431—*Seed maturation*

**Fig. 5:**
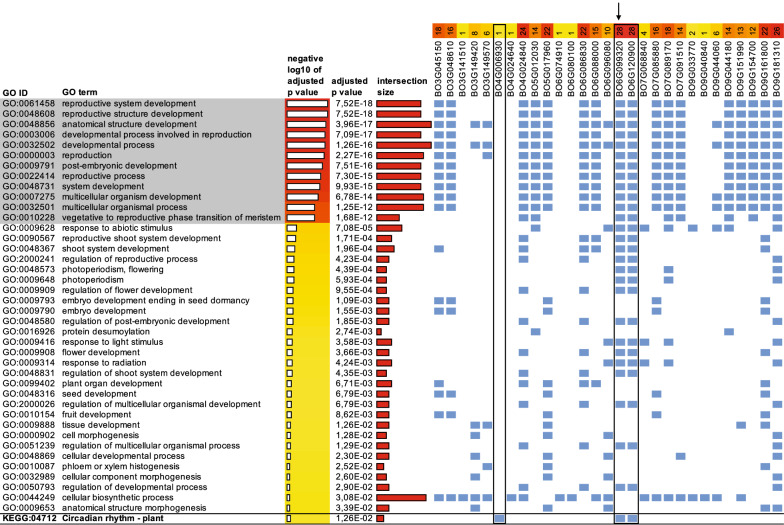
g: Profiler results for genes retained in the pruned GO subgraph. In this analysis, the genes (the columns labeled with BO gene IDs) annotated by GO:0010228 are used as input. In consequence, the GO terms above this term (both in this table and topologically in the GO graph, here shown with gray background) are heavily enriched. Below this are related terms that are also significantly enriched by the gene list. Blue cells indicate which genes contribute to the enrichment. In this analysis, the g:Profiler tool also returns enriched KEGG pathways. In this case this is only—but importantly—“KEGG:04712, Circadian rhythm–plant”add

Terms 2–5 can only start to play a role once the transition of meristem from vegetative to reproductive has commenced (and likewise, seed maturation can only happen in a fully developed flower). Hence, we then zoomed in on only those genes that are (transitively) annotated with GO:0010228 and used these as input for g:Profiler [28], whose results are shown in Fig. 5.

The g:Profiler results show enrichment both for terms above GO:0010228 and those below it. The terms shown with gray background in Fig. 5 are inevitably enriched because we restricted the input gene list to those whose annotations descend from GO:0010228—and therefore also from all upper terms ‘above’ it. More interesting are the terms below it, some of which are more specific and shed light on what, according to these annotation sets, triggers the phase transition: light and photoperiodism. However, this ontology-mediated step’s most salient result is the discovery of KEGG [6] pathway 04712 *Circadian rhythm–plant*, (adjusted *p* = 0.0126*, see Fig. 5) based on the presence of Bo4G006930 (*CIRCADIAN CLOCK ASSOCIATED 1*, *CCA1*), Bo6G099320 (*FLOWERING TIME*, *FT*) and Bo6G120900 (*TWIN SISTER OF FT*, *TSF*).

Genome sequencing of the Jersey kale yielded 1,092,319,676 forward and reverse reads (150 bp) (Additional file 5). Mapped against the TO1000DH3, this resulted in an assembly covering 87.6% of the reference genome with an average depth of 170.6× (see Additional files 5). Variant calling on this assembly produced a total of about 7.5 × 10^6^ raw variants of all types (i.e. including indels and polymorphisms longer than 1 bp). The homology-based SnpEff analysis, which assessed the impact of variants in *Arabidopsis* flowering time pathway, returned results of comparable magnitude as obtained in previous research in the *B. oleracea* cultivar ‘Kashirka’ [13]. For example, most gene copies were affected by, at least, non-synonymous SNPs, and much fewer of those by splicing variation or indels causing frameshifts. Similarly to ‘Kashirka’, one copy of *FT* and one of *FLC* had indels, while the CO copies had non-synonymous SNPs but no indels.

Among the high-impact SNPs (sensu SnpEff) there were two lost start codons. One in a *FUL* copy on q7 inside of inferred QTLs for EF–LF, EF–NF, IF–LF and IF–NF, and one in a *TFL1* copy on q2, outside of any inferred QTLs. There are two more copies of *FUL*, one on p2 and one on p9. Both copies have moderate-impact variants, in both cases comprised of splicing variation and non-synonymous SNPs. The copy on p2 lies within the QTLs inferred for the contrasts IF-NF and EF-NF.

Of *TFL1*, four copies reside, respectively, on q3, p4, q4, and q9. The copy on q3 has four moderate-impact variants, all of which are non-synonymous SNPs. This copy falls within inferred QTLs for IF–LF and EF–LF. The copy on p4 is unaffected by SNPs and outside any QTLs. The long arm copy on the same chromosome has a moderate-impact non-synonymous SNP and falls within the QTLs for IF–LF and EF–IF. The copy on q9 has a moderate-impact non-synonymous SNP and lies within the QTLs for IF–NF and EF–IF.

In addition, there were three genes affected by high-impact SNPs having gained stop codons: *GRP8* (p6), *AP1* (q2), and *VRN2* (q5). None of these lie inside inferred QTLs. Of *GRP8*, two more copies are known to reside, respectively, on p1 and p3. The latter copy has two moderate-impact SNPs, namely an in-frame deletion and a non-synonymous SNP, and lies within QTLs inferred for the contrasts IF–NF, IF–LF, and EF–NF.

Of *AP1* there are two more copies, which both reside on q6, within QTLs that were inferred for all contrasts but EF-IF. One copy is unaffected by moderate impact SNPs, while the other has eight non-synonymous SNPs. *VRN2* has one additional copy, on q8, which lies within the inferred QTLs for EF–NF and EF–IF. This copy has a moderate-impact non-synonymous SNP. Further, detailed results of the SnpEff analysis are available in Additional file 4: Table S4.

## Discussion

Our analysis was somewhat complicated by the proliferation of flowering time cohorts (four pools, where BSA studies typically consider only the two extremes of the trait value distribution) and the commensurate increase in pool contrasts to consider (*n*(*n*-1)/2 working out to six contrasts for four pools). Another complication was the right censoring in waiting time till flowering for pool NF (“non-flowering”): as three plants in this pool flowered after the point of DNA extraction while others never did, the pool is a mixture of very late flowering and non-flowering genotypes. Nevertheless, our results showed consistency across all comparisons in the discovered QTL regions and the GO terms the genes in these regions enriched.

An interesting result was that the extremes in the numbers of enriched GO terms loosely corresponded with the magnitude of the difference in trait values between pools in a comparison: greater differences in flowering time enriched more terms, smaller differences fewer. The same was true for the strength of the patterns detected. The greatest significance in the enrichment of GO:0010228 was observed when contrasting pools separated by intermediate flowering time cohorts. For example, for the contrast IF-NF, *p* < 10^–5^, as shown in the heatmap in Fig. 3. For our present purposes, the increase in the number of enriched terms with greater trait differences constituted a loss in precision: as pools are more different in flowering time, differences in the onset of contingent developmental processes (e.g. seed maturation) have a chance to manifest as well, clouding the picture and yielding more GO terms.

Our key finding was that the iterative pruning of the enriched GO subgraphs substantially reduced the number of candidate QTLs and genes in the result set. We first focused on the upper-level term GO:0003006 (*developmental process involved in reproduction*) and then zoomed in further on nodes subtended by its descendant GO:0010228 (*vegetative to reproductive phase transition of meristem*). This progression was discovered from the data and should therefore be transferrable to other systems without prior knowledge of the underlying genetics or annotations. As a result of this ontology-mediated approach, we reduced the number of candidate genes from 10,469 to a final set of 29 genes resulting from the g:Profiler analysis. Considering that these genes include those previously established as key in regulating flowering time (both in their being homologous to those in *Arabidopsis* and in their variation between *Brassica* cultivars [13]), we view our approach as a powerful complement to existing workflows in processing BSA results.

Our sequencing of the genome of a Jersey kale accession and subsequent homology-based analysis of SNP impact confirms and strengthens the rest of our findings. The pattern in the impact assessment is that copies of genes involved in flowering time regulation that are affected by high-impact SNPs (lost start or gained stop codons) lie outside of the inferred QTLs, and so gene inactivation through lost start or added stop codons does not modulate flowering time differences between our pools. Conversely, other copies of these same genes that have moderate-impact, non-synonymous SNPs do occur within the QTLs. Because inactivated gene copies lie outside of the QTLs while functionally divergent copies (with reference to TO1000DH3) lie within them we infer that the QTLs are indeed ‘where the action is’ in modulating flowering time through the additive effects of non-synonymous SNPs.

A potential weakness is that the ontology-mediated technique’s usefulness hinges on the quality of genome annotations and KEGG pathways: without the combination of good functional characterization (often homology-based) and a known background against which to perform the hypergeometric tests, gene set enrichment analyses cannot work. In practice, this means that such ontology-mediated techniques will be most applicable to well-studied model organisms or reasonably close relatives of *Arabidopsis*. Another potential weakness lies in the non-parametric Gʹ statistic that we used here. Greater power may be attained using a parametric approach such as GWAlpha [30]. However, we found the program in its current iteration to perform certain unsafe operations where existing files can be inadvertently overwritten without warning. We therefore merely note that this is a possible addition to the workflow in future applications if this issue is addressed.

Previous research in flowering time in *B. oleracea* cultivars was purely homology-based, using the pathway in *Arabidopsis* as the backbone on which to map participating gene copies and their variants [13], making different cultivars and species more easily comparable. Using these results, we established similar patterns of variation in the Jersey kale genome as previously have been found in the ‘Kashirka’ cultivar. However, with this approach, the importance of additional genes outside the homologous pathway is never discovered. In contrast, our approach also uncovered the role of Bo6G120900 (TWIN SISTER OF FT, TSF). As such, the ontology-mediated technique we present here is at least complementary, and especially useful in exploring less well-characterized pathways and discovering participating genes.

## Conclusions

We performed a bulk segregant analysis (BSA) across four cohorts of a cross between the Jersey kale and the *B. oleracea* model TO1000DH3 phenotyped on flowering time. The data we collected consisted of high throughput sequencing reads, which we analyzed using standard tools for identifying QTLs in pairwise BSA comparisons. This resulted in numerous regions throughout the genome, though concentrated at loci known from previous homology-based research in flowering time regulation and consistent across pairwise comparisons. To reduce the set of candidate loci to more manageable dimensions, we developed an ontology-mediated approach that limits the result set by focusing on genes annotated with terms contained within relevant subgraphs of the Gene Ontology. This reduced the resulting gene set from tens of thousands to dozens of candidate genes. A further enrichment test led to the pathway for circadian rhythm in plants. The genes that enriched this pathway are attested from previous research as being involved in regulating flowering time, and some of these genes were also identified as having functionally significant variation compared to *Arabidopsis*. As such, we validated and confirmed our ontology-mediated results through a more targeted, homology-based approach. However, the ontology-mediated approach produced additional genes of putative importance, showing that the approach aids in exploration and discovery. We view our method as potentially applicable to the study of other complex traits and therefore make our workflows available as open-source code and a reusable Docker container. This container is available from the ‘Docker hub’ and can consequently be deployed and applied to user data using the standard docker toolchain, for example as ‘docker run -it -v $DATA:/home/ubuntu/data naturalis/brassica-snps’, where the argument $DATA refers to the user data location. More instructions for this are available at https://hub.docker.com/r/naturalis/brassica-snps.

## Supplementary Information

**Additional file 1: Table S1** Flowering time per specimen, data for Figure 1.

**Additional file 2: Table S2** Raw yields of bulk sequencing

**Additional file 3: Table S3** Results of the SEACompare analysis.

**Additional file 4: Table S4** Results of the SnpEff analysis.

**Additional file 5: Table S5** Results of Jersey Kale genome sequencing.

**Additional file 6: **Methods.

## Data Availability

The sequencing datasets generated and/or analysed during the current study are available in the SRA repository, https://www.ncbi.nlm.nih.gov/bioproject/PRJNA564368. The supplementary datasets generated and/or analysed during the current study are available in the Zenodo repository, https://doi.org/10.5281/zenodo.3402201. The source code generated and/or analysed during the current study are available in the Zenodo repository, https://doi.org/10.5281/zenodo.5211374. The docker container generated and/or analysed during the current study are available in the Docker hub repository, https://hub.docker.com/r/naturalis/brassica-snps. The SnpEff procedures generated and/or analysed during the current study are available in the Zenodo repository, https://doi.org/10.5281/zenodo.5211461.
