# Supplementary material for: Refining bulk segregant analyses: ontology-mediated discovery of flowering time genes in Brassica oleracea
Source: Plant Methods. 2022 Jul 4;18:92. doi: 10.1186/s13007-022-00921-y (PMC9252076; doi:10.1186/s13007-022-00921-y)
Supplement: Supplementary file 2 — Additional file 2: Table S2 Raw yields of bulk sequencing [file 13007_2022_921_MOESM2_ESM.docx]

| Phenotype | Bulk size | Bases (Gbp) | Reads  (x10^6^) | GC(%) | Q20(%) | Q30(%) | Coverage a, b | Variants  (x10^6^) |
| --- | --- | --- | --- | --- | --- | --- | --- | --- |
| EF | 11 | 60.3 | 399.4 | 36.93 | 95.02 | 89.25 | 123, 108 | 40.2 |
| IF | 8 | 56.8 | 376.2 | 36.92 | 94.84 | 88.94 | 116, 103 | 43.8 |
| LF | 11 | 54.6 | 361.5 | 37.14 | 96.00 | 91.34 | 112, 100 | 42.9 |
| NF | 9 | 54.3 | 359.6 | 37.13 | 96.84 | 92.81 | 111, 99 | 42.2 |

Summary results of the sequencing of bulks of early (EF), intermediate (IF), late (LF) and “non” flowering (NF, actually not flowering at time of DNA extraction) phenotypes. Bulk size refers to the number of individuals pooled for that phenotype. Coverage is given as a) total read bases divided by reference genome size; and b) average mapped coverage.
