# Supplementary material for: Refining bulk segregant analyses: ontology-mediated discovery of flowering time genes in Brassica oleracea
Source: Plant Methods. 2022 Jul 4;18:92. doi: 10.1186/s13007-022-00921-y (PMC9252076; doi:10.1186/s13007-022-00921-y)
Supplement: Supplementary file 3 — Additional file 3: Table S3 Results of the SEACompare analysis. [file 13007_2022_921_MOESM3_ESM.html]

agriGO - SEA comparison


|  |  |  |  |  |  |  |  |  |  |  |  |  |  |  |  |  |  |  |  |  |  |
| --- | --- | --- | --- | --- | --- | --- | --- | --- | --- | --- | --- | --- | --- | --- | --- | --- | --- | --- | --- | --- | --- |
| **GO Information** | | | | **CM** | | | | | | **ID:577207982** | | **ID:383155352** | | **ID:283547849** | | **ID:847435652** | | **ID:322714491** | | **ID:933274933** | |
| **No** | **GO Term** | **Onto** | **Description** | **1** | **2** | **3** | **4** | **5** | **6** | **FDR** | **Num** | **FDR** | **Num** | **FDR** | **Num** | **FDR** | **Num** | **FDR** | **Num** | **FDR** | **Num** |
| 1 | GO:0006351 | P | transcription, DNA-dependent |  |  |  |  |  |  | 1.6e-26 | 133 | 2.2e-19 | 160 | 3.2e-37 | 244 | 8.2e-38 | 281 | 1.4e-39 | 278 | 8.9e-57 | 382 |
| 2 | GO:0032774 | P | RNA biosynthetic process |  |  |  |  |  |  | 1.6e-26 | 133 | 2.2e-19 | 160 | 3.2e-37 | 244 | 8.2e-38 | 281 | 1.4e-39 | 278 | 8.9e-57 | 382 |
| 3 | GO:0016070 | P | RNA metabolic process |  |  |  |  |  |  | 3.9e-24 | 169 | 5.5e-26 | 239 | 2.4e-41 | 339 | 4.5e-38 | 381 | 2.1e-44 | 389 | 3.3e-59 | 518 |
| 4 | GO:0005975 | P | carbohydrate metabolic process |  |  |  |  |  |  | 3.4e-13 | 92 | 2.1e-30 | 170 | 9.1e-35 | 213 | 4.8e-33 | 239 | 2.3e-44 | 261 | 1e-50 | 328 |
| 5 | GO:0006139 | P | nucleobase, nucleoside, nucleotide and nucleic acid metabolic process |  |  |  |  |  |  | 6.2e-12 | 218 | 8.1e-12 | 314 | 2e-24 | 460 | 5.8e-19 | 514 | 1.5e-22 | 515 | 5.7e-31 | 686 |
| 6 | GO:0045893 | P | positive regulation of transcription, DNA-dependent |  |  |  |  |  |  | 1e-10 | 16 | 3.2e-10 | 18 | --- | --- | --- | --- | --- | --- | --- | --- |
| 7 | GO:0006807 | P | nitrogen compound metabolic process |  |  |  |  |  |  | 1.3e-10 | 243 | 1.8e-15 | 381 | 9.5e-24 | 523 | 3e-17 | 582 | 1.7e-24 | 601 | 8.5e-33 | 796 |
| 8 | GO:0051254 | P | positive regulation of RNA metabolic process |  |  |  |  |  |  | 1.3e-10 | 16 | 8.1e-11 | 19 | --- | --- | --- | --- | --- | --- | --- | --- |
| 9 | GO:0033554 | P | cellular response to stress |  |  |  |  |  |  | 1.6e-10 | 53 | 1.8e-12 | 75 | 3.2e-19 | 107 | 1.7e-11 | 101 | 5.5e-14 | 105 | 5e-21 | 147 |
| 10 | GO:0044262 | P | cellular carbohydrate metabolic process |  |  |  |  |  |  | 2.1e-09 | 52 | 1.6e-24 | 103 | 5e-29 | 131 | 2.5e-30 | 151 | 5.3e-36 | 159 | 5.8e-41 | 199 |
| 11 | GO:0051252 | P | regulation of RNA metabolic process |  |  |  |  |  |  | 2.9e-09 | 89 | 2.4e-06 | 111 | 3e-14 | 172 | 6.3e-12 | 192 | 2.3e-12 | 187 | 1.2e-19 | 261 |
| 12 | GO:0006355 | P | regulation of transcription, DNA-dependent |  |  |  |  |  |  | 3.9e-09 | 88 | 4.1e-06 | 109 | 3e-14 | 171 | 8.5e-12 | 190 | 3.3e-12 | 185 | 2.5e-19 | 258 |
| 13 | GO:0044248 | P | cellular catabolic process |  |  |  |  |  |  | 2.7e-08 | 71 | 9.5e-10 | 102 | 4.5e-18 | 153 | 2.7e-13 | 161 | 3.1e-17 | 169 | 1.1e-21 | 221 |
| 14 | GO:0033036 | P | macromolecule localization |  |  |  |  |  |  | 4.1e-08 | 52 | 6e-15 | 88 | 1.8e-15 | 107 | 7.9e-11 | 109 | 1.1e-15 | 120 | 8.9e-22 | 163 |
| 15 | GO:0042743 | P | hydrogen peroxide metabolic process |  |  |  |  |  |  | 4.1e-08 | 14 | 5.7e-05 | 12 | 4.6e-11 | 23 | 1.5e-10 | 25 | 1.6e-08 | 21 | --- | --- |
| 16 | GO:0005976 | P | polysaccharide metabolic process |  |  |  |  |  |  | 5.4e-08 | 28 | 1.7e-26 | 67 | 1.3e-23 | 72 | 7.4e-24 | 81 | 2.6e-32 | 93 | 2.2e-36 | 115 |
| 17 | GO:0051716 | P | cellular response to stimulus |  |  |  |  |  |  | 8.7e-08 | 75 | 5.8e-11 | 115 | 1.2e-18 | 167 | 4.8e-08 | 152 | 2.7e-13 | 169 | 7.6e-17 | 221 |
| 18 | GO:0000272 | P | polysaccharide catabolic process |  |  |  |  |  |  | 1.3e-07 | 12 | --- | --- | --- | --- | --- | --- | --- | --- | --- | --- |
| 19 | GO:0006350 | P | transcription |  |  |  |  |  |  | 2.1e-07 | 133 | 0.0069 | 160 | 8.2e-08 | 244 | 1.4e-06 | 281 | 1.3e-07 | 278 | 2.5e-12 | 382 |
| 20 | GO:0006605 | P | protein targeting |  |  |  |  |  |  | 2.7e-07 | 27 | 5e-10 | 40 | 3e-13 | 54 | 1.6e-05 | 42 | 1.6e-09 | 51 | 4.1e-14 | 72 |
| 21 | GO:0051641 | P | cellular localization |  |  |  |  |  |  | 1.2e-06 | 55 | 2.1e-12 | 93 | 2.8e-11 | 109 | 3.2e-10 | 123 | 3.2e-12 | 126 | 7.2e-18 | 173 |
| 22 | GO:0000723 | P | telomere maintenance |  |  |  |  |  |  | 1.2e-06 | 10 | --- | --- | --- | --- | --- | --- | --- | --- | --- | --- |
| 23 | GO:0032200 | P | telomere organization |  |  |  |  |  |  | 1.2e-06 | 10 | --- | --- | --- | --- | --- | --- | --- | --- | --- | --- |
| 24 | GO:0009891 | P | positive regulation of biosynthetic process |  |  |  |  |  |  | 1.9e-06 | 18 | 2.9e-05 | 20 | 2.6e-07 | 28 | 2.4e-10 | 38 | 6e-09 | 34 | 6e-13 | 48 |
| 25 | GO:0008104 | P | protein localization |  |  |  |  |  |  | 1.9e-06 | 44 | 2.9e-13 | 78 | 7.3e-14 | 95 | 7.7e-10 | 97 | 2e-12 | 102 | 1.3e-18 | 142 |
| 26 | GO:0031328 | P | positive regulation of cellular biosynthetic process |  |  |  |  |  |  | 1.9e-06 | 18 | 2.9e-05 | 20 | 2.6e-07 | 28 | 2.4e-10 | 38 | 6e-09 | 34 | 6e-13 | 48 |
| 27 | GO:0048522 | P | positive regulation of cellular process |  |  |  |  |  |  | 2e-06 | 26 | 1.9e-05 | 31 | 2.4e-05 | 37 | 4.7e-06 | 45 | 2.8e-06 | 44 | 7e-11 | 66 |
| 28 | GO:0042592 | P | homeostatic process |  |  |  |  |  |  | 2.6e-06 | 30 | 7.6e-08 | 43 | 1.4e-06 | 48 | 7.7e-10 | 65 | 0.00048 | 45 | 7e-11 | 78 |
| 29 | GO:0031325 | P | positive regulation of cellular metabolic process |  |  |  |  |  |  | 4e-06 | 19 | 3.1e-06 | 24 | 1.9e-07 | 31 | 8.9e-09 | 38 | 7.2e-09 | 37 | 8.9e-13 | 52 |
| 30 | GO:0009893 | P | positive regulation of metabolic process |  |  |  |  |  |  | 4.4e-06 | 19 | 3.6e-06 | 24 | 2.3e-07 | 31 | 1e-08 | 38 | 9.1e-09 | 37 | 1.2e-12 | 52 |
| 31 | GO:0045941 | P | positive regulation of transcription |  |  |  |  |  |  | 4.4e-06 | 16 | 3.6e-05 | 18 | 4.9e-07 | 25 | 1.9e-09 | 33 | 6.2e-09 | 31 | 2.3e-11 | 41 |
| 32 | GO:0010628 | P | positive regulation of gene expression |  |  |  |  |  |  | 4.4e-06 | 16 | 3.6e-05 | 18 | 4.9e-07 | 25 | 1.9e-09 | 33 | 6.2e-09 | 31 | 2.3e-11 | 41 |
| 33 | GO:0044264 | P | cellular polysaccharide metabolic process |  |  |  |  |  |  | 5.7e-06 | 22 | 1.9e-19 | 52 | 4.4e-20 | 61 | 1.6e-20 | 69 | 1.7e-24 | 74 | 1.7e-28 | 93 |
| 34 | GO:0006996 | P | organelle organization |  |  |  |  |  |  | 6e-06 | 57 | 8.4e-17 | 112 | 1.4e-19 | 143 | 1.9e-18 | 161 | 7.4e-22 | 166 | 9.2e-26 | 212 |
| 35 | GO:0070727 | P | cellular macromolecule localization |  |  |  |  |  |  | 6.4e-06 | 38 | 1.9e-11 | 66 | 7.6e-12 | 80 | 1e-07 | 79 | 1.1e-10 | 86 | 5.2e-14 | 114 |
| 36 | GO:0065008 | P | regulation of biological quality |  |  |  |  |  |  | 8.4e-06 | 58 | 1.6e-07 | 87 | 5.6e-07 | 105 | 1.7e-05 | 116 | 0.00032 | 105 | 1.2e-08 | 155 |
| 37 | GO:0010557 | P | positive regulation of macromolecule biosynthetic process |  |  |  |  |  |  | 1.1e-05 | 16 | 9.4e-05 | 18 | 1.8e-06 | 25 | 1.1e-09 | 35 | 3.1e-08 | 31 | 5.9e-11 | 42 |
| 38 | GO:0045935 | P | positive regulation of nucleobase, nucleoside, nucleotide and nucleic acid metabolic process |  |  |  |  |  |  | 1.1e-05 | 16 | 3e-05 | 19 | 5.6e-07 | 26 | 9.3e-09 | 33 | 9.4e-09 | 32 | 5.9e-11 | 42 |
| 39 | GO:0044042 | P | glucan metabolic process |  |  |  |  |  |  | 1.1e-05 | 18 | 2.7e-10 | 31 | 9.8e-10 | 35 | 3e-06 | 32 | 5.6e-11 | 41 | 4.9e-13 | 52 |
| 40 | GO:0034613 | P | cellular protein localization |  |  |  |  |  |  | 1.1e-05 | 36 | 1.6e-11 | 64 | 3.9e-12 | 78 | 2.5e-08 | 78 | 2.1e-10 | 82 | 1.1e-14 | 112 |
| 41 | GO:0009058 | P | biosynthetic process |  |  |  |  |  |  | 1.1e-05 | 274 | 3.4e-05 | 407 | 2.4e-14 | 601 | 2.5e-08 | 667 | 9.7e-14 | 688 | 3.1e-17 | 900 |
| 42 | GO:0051649 | P | establishment of localization in cell |  |  |  |  |  |  | 1.1e-05 | 49 | 7.4e-12 | 87 | 3.6e-11 | 103 | 1.4e-09 | 114 | 1.6e-11 | 117 | 1.5e-17 | 163 |
| 43 | GO:0051173 | P | positive regulation of nitrogen compound metabolic process |  |  |  |  |  |  | 1.1e-05 | 16 | 3.6e-05 | 19 | 6.8e-07 | 26 | 1.2e-08 | 33 | 1.2e-08 | 32 | 7.9e-11 | 42 |
| 44 | GO:0006612 | P | protein targeting to membrane |  |  |  |  |  |  | 1.2e-05 | 11 | 1.5e-07 | 16 | 2.5e-07 | 18 | 0.0056 | 12 | 0.00032 | 14 | 9.7e-08 | 23 |
| 45 | GO:0006886 | P | intracellular protein transport |  |  |  |  |  |  | 1.3e-05 | 35 | 3e-11 | 62 | 2.3e-12 | 77 | 6e-08 | 75 | 2.5e-10 | 80 | 7.8e-15 | 110 |
| 46 | GO:0016052 | P | carbohydrate catabolic process |  |  |  |  |  |  | 1.9e-05 | 21 | 2.3e-14 | 44 | 8.7e-15 | 52 | 5.2e-16 | 61 | 4.7e-19 | 65 | 9.4e-21 | 79 |
| 47 | GO:0045184 | P | establishment of protein localization |  |  |  |  |  |  | 2.3e-05 | 39 | 1.8e-12 | 73 | 1.9e-13 | 90 | 4.2e-09 | 90 | 6.5e-12 | 96 | 7.2e-18 | 134 |
| 48 | GO:0015031 | P | protein transport |  |  |  |  |  |  | 2.3e-05 | 39 | 1.8e-12 | 73 | 1.9e-13 | 90 | 4.2e-09 | 90 | 6.5e-12 | 96 | 7.2e-18 | 134 |
| 49 | GO:0010604 | P | positive regulation of macromolecule metabolic process |  |  |  |  |  |  | 2.6e-05 | 16 | 8.9e-06 | 21 | 7.5e-07 | 27 | 6.4e-09 | 35 | 6e-09 | 34 | 2e-11 | 45 |
| 50 | GO:0044249 | P | cellular biosynthetic process |  |  |  |  |  |  | 3.1e-05 | 262 | 3.1e-05 | 394 | 1.1e-12 | 571 | 5.6e-08 | 642 | 2.6e-12 | 656 | 2.2e-16 | 865 |
| 51 | GO:0051179 | P | localization |  |  |  |  |  |  | 3.2e-05 | 122 | 1.2e-10 | 207 | 3.7e-08 | 246 | 1.4e-06 | 281 | 4.2e-08 | 281 | 1.6e-12 | 383 |
| 52 | GO:0080135 | P | regulation of cellular response to stress |  |  |  |  |  |  | 6e-05 | 8 | 8.1e-08 | 13 | --- | --- | 0.014 | 8 | 3.1e-05 | 12 | --- | --- |
| 53 | GO:0042542 | P | response to hydrogen peroxide |  |  |  |  |  |  | 7e-05 | 13 | 0.00079 | 14 | 1.8e-05 | 20 | 0.028 | 15 | 1.8e-06 | 24 | 7e-08 | 31 |
| 54 | GO:0000302 | P | response to reactive oxygen species |  |  |  |  |  |  | 8.8e-05 | 16 | 0.012 | 15 | 0.00058 | 22 | --- | --- | 0.00015 | 26 | 5.1e-06 | 35 |
| 55 | GO:0031348 | P | negative regulation of defense response |  |  |  |  |  |  | 0.0001 | 9 | 0.0012 | 9 | 0.00018 | 12 | --- | --- | --- | --- | 0.00018 | 15 |
| 56 | GO:0070301 | P | cellular response to hydrogen peroxide |  |  |  |  |  |  | 0.0001 | 7 | 0.0038 | 6 | 0.00079 | 8 | 0.00018 | 10 | --- | --- | --- | --- |
| 57 | GO:0034614 | P | cellular response to reactive oxygen species |  |  |  |  |  |  | 0.0001 | 7 | 0.00076 | 7 | 0.00079 | 8 | --- | --- | --- | --- | --- | --- |
| 58 | GO:0042744 | P | hydrogen peroxide catabolic process |  |  |  |  |  |  | 0.0001 | 7 | 0.0038 | 6 | 0.00079 | 8 | 0.00018 | 10 | --- | --- | --- | --- |
| 59 | GO:0006800 | P | oxygen and reactive oxygen species metabolic process |  |  |  |  |  |  | 0.00011 | 14 | 0.0043 | 14 | 9e-06 | 23 | 4.1e-06 | 27 | 0.00025 | 22 | 6.6e-10 | 39 |
| 60 | GO:0034599 | P | cellular response to oxidative stress |  |  |  |  |  |  | 0.00015 | 7 | 0.0011 | 7 | 0.0012 | 8 | 6.4e-05 | 11 | --- | --- | --- | --- |
| 61 | GO:0006753 | P | nucleoside phosphate metabolic process |  |  |  |  |  |  | 0.00019 | 23 | 0.016 | 24 | 1.4e-08 | 48 | 1.6e-07 | 52 | 1.1e-06 | 48 | 8.2e-10 | 67 |
| 62 | GO:0009117 | P | nucleotide metabolic process |  |  |  |  |  |  | 0.00019 | 23 | 0.016 | 24 | 1.4e-08 | 48 | 1.6e-07 | 52 | 1.1e-06 | 48 | 8.2e-10 | 67 |
| 63 | GO:0048446 | P | petal morphogenesis |  |  |  |  |  |  | 0.00024 | 6 | 0.0012 | 6 | 0.00096 | 7 | --- | --- | 0.0099 | 6 | --- | --- |
| 64 | GO:0048518 | P | positive regulation of biological process |  |  |  |  |  |  | 0.00031 | 28 | 0.0029 | 34 | 0.0088 | 40 | 0.018 | 46 | 0.0025 | 48 | 8.7e-06 | 70 |
| 65 | GO:0009886 | P | post-embryonic morphogenesis |  |  |  |  |  |  | 0.00033 | 10 | 9.6e-05 | 13 | 1.6e-09 | 23 | 1.1e-09 | 26 | 6e-09 | 24 | --- | --- |
| 66 | GO:0051276 | P | chromosome organization |  |  |  |  |  |  | 0.00033 | 25 | 1.8e-14 | 57 | 4.5e-13 | 64 | 8.8e-14 | 75 | 1e-15 | 77 | 1.3e-18 | 98 |
| 67 | GO:0070887 | P | cellular response to chemical stimulus |  |  |  |  |  |  | 0.00033 | 40 | 0.0002 | 56 | 1.7e-06 | 78 | 0.0087 | 73 | 4.9e-06 | 86 | 7e-08 | 114 |
| 68 | GO:0006662 | P | glycerol ether metabolic process |  |  |  |  |  |  | 0.00036 | 6 | --- | --- | 0.0015 | 7 | 0.024 | 6 | --- | --- | --- | --- |
| 69 | GO:0048464 | P | flower calyx development |  |  |  |  |  |  | 0.00036 | 6 | 0.0019 | 6 | 0.0072 | 6 | 0.0014 | 8 | --- | --- | --- | --- |
| 70 | GO:0018904 | P | organic ether metabolic process |  |  |  |  |  |  | 0.00036 | 6 | --- | --- | 0.0015 | 7 | 0.024 | 6 | --- | --- | --- | --- |
| 71 | GO:0065007 | P | biological regulation |  |  |  |  |  |  | 0.00036 | 221 | 0.00038 | 332 | 8.2e-08 | 465 | 0.0059 | 502 | 0.00032 | 500 | 3.3e-07 | 678 |
| 72 | GO:0048442 | P | sepal development |  |  |  |  |  |  | 0.00036 | 6 | 0.0019 | 6 | 0.0072 | 6 | 0.0014 | 8 | --- | --- | --- | --- |
| 73 | GO:0034637 | P | cellular carbohydrate biosynthetic process |  |  |  |  |  |  | 0.00038 | 22 | 4.6e-10 | 43 | 1.2e-09 | 50 | 3.5e-09 | 56 | 9.7e-14 | 65 | 3.5e-15 | 80 |
| 74 | GO:0032787 | P | monocarboxylic acid metabolic process |  |  |  |  |  |  | 0.0004 | 37 | 2.7e-11 | 73 | 2.1e-12 | 91 | 7.8e-07 | 86 | 8.3e-11 | 97 | 1.3e-11 | 120 |
| 75 | GO:0051234 | P | establishment of localization |  |  |  |  |  |  | 0.0005 | 112 | 9.3e-10 | 197 | 4.7e-07 | 232 | 7.6e-06 | 267 | 4.4e-07 | 266 | 2.2e-11 | 365 |
| 76 | GO:0043436 | P | oxoacid metabolic process |  |  |  |  |  |  | 0.0005 | 62 | 3.5e-19 | 141 | 1.4e-15 | 160 | 9e-09 | 158 | 4.6e-17 | 185 | 3.2e-17 | 226 |
| 77 | GO:0046907 | P | intracellular transport |  |  |  |  |  |  | 0.0005 | 40 | 1.2e-10 | 77 | 6.5e-10 | 91 | 2.4e-07 | 96 | 7.5e-10 | 102 | 6.2e-15 | 142 |
| 78 | GO:0019752 | P | carboxylic acid metabolic process |  |  |  |  |  |  | 0.0005 | 62 | 3.5e-19 | 141 | 1.4e-15 | 160 | 9e-09 | 158 | 4.6e-17 | 185 | 3.2e-17 | 226 |
| 79 | GO:0006082 | P | organic acid metabolic process |  |  |  |  |  |  | 0.00051 | 62 | 3.5e-19 | 141 | 1.4e-15 | 160 | 9.3e-09 | 158 | 4.8e-17 | 185 | 3.4e-17 | 226 |
| 80 | GO:0044265 | P | cellular macromolecule catabolic process |  |  |  |  |  |  | 0.00051 | 40 | 2.9e-06 | 64 | 3.9e-11 | 95 | 7.5e-12 | 113 | 1.7e-09 | 101 | 8.5e-11 | 128 |
| 81 | GO:0046394 | P | carboxylic acid biosynthetic process |  |  |  |  |  |  | 0.00056 | 37 | 3.2e-10 | 71 | 3e-10 | 86 | 7.7e-05 | 79 | 1.1e-10 | 98 | 5.5e-09 | 112 |
| 82 | GO:0016053 | P | organic acid biosynthetic process |  |  |  |  |  |  | 0.00056 | 37 | 3.2e-10 | 71 | 3e-10 | 86 | 7.7e-05 | 79 | 1.1e-10 | 98 | 5.5e-09 | 112 |
| 83 | GO:0006281 | P | DNA repair |  |  |  |  |  |  | 0.00064 | 24 | 4.1e-07 | 41 | 1.9e-11 | 60 | 1.2e-08 | 61 | 1.6e-06 | 53 | 1e-08 | 71 |
| 84 | GO:0031399 | P | regulation of protein modification process |  |  |  |  |  |  | 0.00077 | 6 | 2e-05 | 9 | 0.0036 | 7 | --- | --- | 0.008 | 7 | --- | --- |
| 85 | GO:0009987 | P | cellular process |  |  |  |  |  |  | 0.00082 | 535 | 3.8e-08 | 879 | 9.4e-14 | 1196 | 6e-08 | 1378 | 3.5e-14 | 1394 | 2.5e-19 | 1840 |
| 86 | GO:0006974 | P | response to DNA damage stimulus |  |  |  |  |  |  | 0.00094 | 24 | 3.4e-07 | 42 | 7.6e-12 | 62 | 7e-09 | 63 | 1.7e-06 | 54 | 1.7e-09 | 75 |
| 87 | GO:0009311 | P | oligosaccharide metabolic process |  |  |  |  |  |  | 0.00094 | 11 | 0.0021 | 13 | 1.1e-07 | 24 | 0.0046 | 17 | 1.3e-05 | 22 | 1.4e-06 | 28 |
| 88 | GO:0030243 | P | cellulose metabolic process |  |  |  |  |  |  | 0.00094 | 7 | 1.2e-05 | 11 | 2.8e-05 | 12 | 0.00085 | 11 | 2.8e-07 | 16 | --- | --- |
| 89 | GO:0055086 | P | nucleobase, nucleoside and nucleotide metabolic process |  |  |  |  |  |  | 0.00094 | 24 | 0.00053 | 33 | 4e-08 | 53 | 3.6e-07 | 58 | 3.5e-08 | 59 | 7.5e-11 | 79 |
| 90 | GO:0006810 | P | transport |  |  |  |  |  |  | 0.00094 | 110 | 2e-09 | 195 | 1.5e-06 | 228 | 4.9e-05 | 260 | 2.6e-06 | 260 | 3e-10 | 357 |
| 91 | GO:0042180 | P | cellular ketone metabolic process |  |  |  |  |  |  | 0.00094 | 62 | 1.6e-19 | 145 | 6.9e-16 | 164 | 4e-09 | 163 | 1.8e-17 | 190 | 1.2e-17 | 232 |
| 92 | GO:0044238 | P | primary metabolic process |  |  |  |  |  |  | 0.00098 | 423 | 9.3e-07 | 687 | 3.5e-10 | 926 | 1.4e-05 | 1061 | 1.6e-10 | 1079 | 4e-13 | 1413 |
| 93 | GO:0006090 | P | pyruvate metabolic process |  |  |  |  |  |  | 0.001 | 8 | 6.7e-06 | 13 | 1.5e-06 | 16 | 5.3e-06 | 17 | 5.6e-07 | 18 | 1.6e-09 | 25 |
| 94 | GO:0009220 | P | pyrimidine ribonucleotide biosynthetic process |  |  |  |  |  |  | 0.0012 | 5 | --- | --- | --- | --- | --- | --- | --- | --- | --- | --- |
| 95 | GO:0031347 | P | regulation of defense response |  |  |  |  |  |  | 0.0014 | 12 | 5.2e-06 | 20 | 8.8e-08 | 27 | 0.0069 | 19 | 9.2e-05 | 23 | 3.7e-07 | 33 |
| 96 | GO:0048585 | P | negative regulation of response to stimulus |  |  |  |  |  |  | 0.0024 | 13 | --- | --- | 0.023 | 17 | --- | --- | --- | --- | --- | --- |
| 97 | GO:0033037 | P | polysaccharide localization |  |  |  |  |  |  | 0.0026 | 7 | --- | --- | --- | --- | --- | --- | --- | --- | --- | --- |
| 98 | GO:0043414 | P | macromolecule methylation |  |  |  |  |  |  | 0.0026 | 13 | 4.5e-09 | 28 | 3.6e-08 | 31 | 3.3e-08 | 35 | 1.1e-09 | 37 | 3.8e-09 | 42 |
| 99 | GO:0008152 | P | metabolic process |  |  |  |  |  |  | 0.0029 | 485 | 9.4e-05 | 771 | 1.1e-08 | 1055 | 0.0028 | 1195 | 9.2e-09 | 1228 | 1.5e-10 | 1605 |
| 100 | GO:0043067 | P | regulation of programmed cell death |  |  |  |  |  |  | 0.0031 | 9 | 7.6e-05 | 14 | 0.00012 | 16 | --- | --- | 0.031 | 12 | 2.4e-05 | 22 |
| 101 | GO:0006730 | P | one-carbon metabolic process |  |  |  |  |  |  | 0.0033 | 15 | 1.2e-10 | 35 | 3.4e-09 | 38 | 2e-07 | 39 | 1.2e-10 | 45 | 6.6e-10 | 51 |
| 102 | GO:0000278 | P | mitotic cell cycle |  |  |  |  |  |  | 0.0034 | 10 | 3.7e-06 | 18 | 3.7e-06 | 21 | 8.7e-06 | 23 | 1.1e-05 | 22 | 1.1e-08 | 32 |
| 103 | GO:0044275 | P | cellular carbohydrate catabolic process |  |  |  |  |  |  | 0.0034 | 16 | 1.8e-06 | 29 | 2e-10 | 43 | 9.1e-12 | 52 | 1.1e-10 | 48 | 4.1e-13 | 62 |
| 104 | GO:0017038 | P | protein import |  |  |  |  |  |  | 0.0034 | 13 | 4.1e-05 | 21 | 5.2e-06 | 27 | 0.0022 | 24 | 0.00041 | 25 | 2.6e-06 | 36 |
| 105 | GO:0010228 | P | vegetative to reproductive phase transition of meristem |  |  |  |  |  |  | 0.0035 | 15 | 0.023 | 17 | 0.0055 | 23 | 0.01 | 26 | --- | --- | 0.00099 | 34 |
| 106 | GO:0006633 | P | fatty acid biosynthetic process |  |  |  |  |  |  | 0.0036 | 17 | 0.0021 | 23 | 8.1e-06 | 35 | 0.00011 | 37 | 7e-06 | 39 | 0.0002 | 42 |
| 107 | GO:0006073 | P | cellular glucan metabolic process |  |  |  |  |  |  | 0.0037 | 13 | 1.8e-06 | 24 | 7.2e-07 | 29 | 1.4e-05 | 30 | 2.2e-07 | 33 | 1e-08 | 42 |
| 108 | GO:0034976 | P | response to endoplasmic reticulum stress |  |  |  |  |  |  | 0.0037 | 7 | 0.0016 | 9 | 0.00024 | 12 | 0.015 | 10 | 0.00089 | 12 | 7.9e-05 | 16 |
| 109 | GO:0009697 | P | salicylic acid biosynthetic process |  |  |  |  |  |  | 0.0041 | 5 | 0.0028 | 6 | --- | --- | 0.033 | 6 | 0.0054 | 7 | --- | --- |
| 110 | GO:0009218 | P | pyrimidine ribonucleotide metabolic process |  |  |  |  |  |  | 0.0041 | 5 | --- | --- | 9.6e-05 | 9 | --- | --- | 0.0013 | 8 | --- | --- |
| 111 | GO:0015674 | P | di-, tri-valent inorganic cation transport |  |  |  |  |  |  | 0.0045 | 9 | 0.015 | 10 | 0.036 | 11 | --- | --- | --- | --- | 0.001 | 19 |
| 112 | GO:0006732 | P | coenzyme metabolic process |  |  |  |  |  |  | 0.0046 | 20 | 0.00042 | 30 | 2.7e-07 | 46 | 2.6e-06 | 50 | 2.1e-08 | 54 | 3.4e-09 | 67 |
| 113 | GO:0032259 | P | methylation |  |  |  |  |  |  | 0.0047 | 13 | 1.9e-08 | 28 | 1.5e-07 | 31 | 1.6e-07 | 35 | 6e-09 | 37 | 2.1e-08 | 42 |
| 114 | GO:0044237 | P | cellular metabolic process |  |  |  |  |  |  | 0.0048 | 404 | 7.1e-06 | 659 | 4.9e-11 | 909 | 1.6e-05 | 1031 | 1.7e-10 | 1050 | 6.3e-14 | 1383 |
| 115 | GO:0034645 | P | cellular macromolecule biosynthetic process |  |  |  |  |  |  | 0.0049 | 188 | --- | --- | 6.1e-06 | 399 | 0.00065 | 457 | 3.3e-05 | 454 | 1.5e-07 | 606 |
| 116 | GO:0009059 | P | macromolecule biosynthetic process |  |  |  |  |  |  | 0.0049 | 189 | --- | --- | 6.5e-06 | 401 | 0.00061 | 460 | 3e-05 | 457 | 1.3e-07 | 610 |
| 117 | GO:0010941 | P | regulation of cell death |  |  |  |  |  |  | 0.005 | 9 | 0.00017 | 14 | 0.00029 | 16 | --- | --- | --- | --- | 2.6e-05 | 23 |
| 118 | GO:0006259 | P | DNA metabolic process |  |  |  |  |  |  | 0.0051 | 33 | 4e-08 | 64 | 3.3e-12 | 90 | 2.8e-10 | 98 | 3.6e-09 | 91 | 2.1e-12 | 122 |
| 119 | GO:0033692 | P | cellular polysaccharide biosynthetic process |  |  |  |  |  |  | 0.0054 | 13 | 4.8e-10 | 31 | 4.6e-11 | 38 | 6e-11 | 43 | 1e-12 | 45 | 1.3e-15 | 58 |
| 120 | GO:0044247 | P | cellular polysaccharide catabolic process |  |  |  |  |  |  | 0.0054 | 6 | 1.1e-09 | 16 | --- | --- | --- | --- | --- | --- | --- | --- |
| 121 | GO:0005984 | P | disaccharide metabolic process |  |  |  |  |  |  | 0.0056 | 9 | 0.0022 | 12 | 7.5e-07 | 21 | 0.02 | 14 | 7.5e-05 | 19 | 1.2e-05 | 24 |
| 122 | GO:0006084 | P | acetyl-CoA metabolic process |  |  |  |  |  |  | 0.0058 | 8 | --- | --- | 0.0039 | 12 | --- | --- | 0.0048 | 13 | 0.00013 | 19 |
| 123 | GO:0000165 | P | MAPKKK cascade |  |  |  |  |  |  | 0.0074 | 5 | 0.0053 | 6 | 0.0012 | 8 | --- | --- | --- | --- | 0.001 | 10 |
| 124 | GO:0006081 | P | cellular aldehyde metabolic process |  |  |  |  |  |  | 0.0074 | 7 | 8.1e-11 | 20 | 1.2e-05 | 15 | 4e-05 | 16 | 8.3e-10 | 23 | 1.2e-09 | 26 |
| 125 | GO:0034641 | P | cellular nitrogen compound metabolic process |  |  |  |  |  |  | 0.0075 | 38 | 1.1e-16 | 97 | 2.5e-14 | 110 | 1.5e-09 | 111 | 7.5e-19 | 136 | 2.4e-20 | 168 |
| 126 | GO:0009259 | P | ribonucleotide metabolic process |  |  |  |  |  |  | 0.0075 | 13 | --- | --- | 0.0048 | 21 | 0.007 | 24 | --- | --- | 0.00083 | 31 |
| 127 | GO:0080134 | P | regulation of response to stress |  |  |  |  |  |  | 0.0075 | 12 | 2.7e-05 | 21 | 3.1e-06 | 27 | --- | --- | 0.00063 | 24 | 8e-06 | 34 |
| 128 | GO:0045088 | P | regulation of innate immune response |  |  |  |  |  |  | 0.0089 | 8 | 4.8e-05 | 14 | 5.9e-06 | 18 | --- | --- | 0.0012 | 15 | 4.6e-06 | 23 |
| 129 | GO:0048609 | P | reproductive process in a multicellular organism |  |  |  |  |  |  | 0.0089 | 8 | 3e-06 | 16 | 7.5e-05 | 16 | 0.006 | 14 | 0.00013 | 17 | 1.5e-06 | 24 |
| 130 | GO:0008610 | P | lipid biosynthetic process |  |  |  |  |  |  | 0.0089 | 34 | 2.2e-06 | 62 | 6.2e-07 | 78 | 1e-05 | 86 | 8.8e-08 | 91 | 5.7e-09 | 116 |
| 131 | GO:0000271 | P | polysaccharide biosynthetic process |  |  |  |  |  |  | 0.009 | 14 | 6.7e-09 | 32 | 4.1e-10 | 40 | 2.6e-10 | 46 | 5.4e-12 | 48 | 3.3e-15 | 63 |
| 132 | GO:0050789 | P | regulation of biological process |  |  |  |  |  |  | 0.0092 | 187 | 0.022 | 278 | 8.8e-06 | 401 | --- | --- | 0.0034 | 434 | 3.2e-05 | 586 |
| 133 | GO:0006006 | P | glucose metabolic process |  |  |  |  |  |  | 0.0095 | 12 | 0.0056 | 16 | 5.2e-06 | 27 | 5.6e-07 | 33 | 1e-05 | 29 | 7.8e-09 | 42 |
| 134 | GO:0006629 | P | lipid metabolic process |  |  |  |  |  |  | 0.0096 | 55 | 4.4e-07 | 101 | 9e-06 | 119 | 8.8e-05 | 135 | 1.3e-07 | 145 | 5.5e-09 | 187 |
| 135 | GO:0052545 | P | callose localization |  |  |  |  |  |  | 0.012 | 6 | --- | --- | --- | --- | --- | --- | --- | --- | --- | --- |
| 136 | GO:0045488 | P | pectin metabolic process |  |  |  |  |  |  | 0.012 | 6 | 6.8e-13 | 21 | --- | --- | --- | --- | --- | --- | --- | --- |
| 137 | GO:0006221 | P | pyrimidine nucleotide biosynthetic process |  |  |  |  |  |  | 0.012 | 5 | --- | --- | 2.4e-05 | 11 | 3.9e-05 | 12 | 0.0004 | 10 | --- | --- |
| 138 | GO:0009653 | P | anatomical structure morphogenesis |  |  |  |  |  |  | 0.012 | 44 | --- | --- | 0.0072 | 81 | --- | --- | 0.026 | 89 | 0.0004 | 126 |
| 139 | GO:0002682 | P | regulation of immune system process |  |  |  |  |  |  | 0.013 | 8 | 9.2e-05 | 14 | 1.2e-05 | 18 | --- | --- | 0.0021 | 15 | 1.1e-05 | 23 |
| 140 | GO:0032504 | P | multicellular organism reproduction |  |  |  |  |  |  | 0.013 | 8 | 3.5e-07 | 18 | 4.4e-05 | 17 | 0.0042 | 15 | 2.7e-05 | 19 | 1.3e-06 | 25 |
| 141 | GO:0050776 | P | regulation of immune response |  |  |  |  |  |  | 0.013 | 8 | 9.2e-05 | 14 | 1.2e-05 | 18 | --- | --- | 0.0021 | 15 | 1.1e-05 | 23 |
| 142 | GO:0016043 | P | cellular component organization |  |  |  |  |  |  | 0.013 | 71 | 4.8e-11 | 146 | 4.5e-13 | 189 | 1.7e-11 | 215 | 2.1e-15 | 224 | 1.2e-16 | 281 |
| 143 | GO:0009056 | P | catabolic process |  |  |  |  |  |  | 0.014 | 77 | 0.00055 | 123 | 6.8e-06 | 169 | 0.0063 | 178 | 2.7e-06 | 196 | 2.1e-06 | 247 |
| 144 | GO:0048465 | P | corolla development |  |  |  |  |  |  | 0.014 | 6 | --- | --- | 0.024 | 8 | 0.033 | 9 | --- | --- | 0.0051 | 12 |
| 145 | GO:0016051 | P | carbohydrate biosynthetic process |  |  |  |  |  |  | 0.014 | 24 | 4.1e-06 | 45 | 1.3e-05 | 53 | 2.1e-05 | 61 | 1.7e-08 | 69 | 1.3e-09 | 87 |
| 146 | GO:0048441 | P | petal development |  |  |  |  |  |  | 0.014 | 6 | --- | --- | 0.024 | 8 | 0.033 | 9 | --- | --- | 0.0051 | 12 |
| 147 | GO:0048449 | P | floral organ formation |  |  |  |  |  |  | 0.014 | 6 | 0.0049 | 8 | 0.0078 | 9 | 0.012 | 10 | 0.0068 | 10 | 5.3e-05 | 16 |
| 148 | GO:0000160 | P | two-component signal transduction system (phosphorelay) |  |  |  |  |  |  | 0.015 | 12 | --- | --- | 0.0068 | 20 | --- | --- | --- | --- | --- | --- |
| 149 | GO:0051789 | P | response to protein stimulus |  |  |  |  |  |  | 0.015 | 5 | 0.00061 | 8 | 0.045 | 6 | 0.0041 | 9 | 0.03 | 7 | 2.1e-05 | 14 |
| 150 | GO:0006887 | P | exocytosis |  |  |  |  |  |  | 0.015 | 7 | --- | --- | --- | --- | --- | --- | --- | --- | 0.02 | 13 |
| 151 | GO:0010310 | P | regulation of hydrogen peroxide metabolic process |  |  |  |  |  |  | 0.015 | 5 | 0.013 | 6 | 0.045 | 6 | --- | --- | --- | --- | 0.0011 | 11 |
| 152 | GO:0009250 | P | glucan biosynthetic process |  |  |  |  |  |  | 0.015 | 9 | 2.5e-05 | 17 | 6.3e-07 | 23 | 6e-06 | 24 | 7.3e-07 | 25 | 3.1e-08 | 32 |
| 153 | GO:0051246 | P | regulation of protein metabolic process |  |  |  |  |  |  | 0.015 | 9 | 0.00029 | 15 | 0.025 | 13 | --- | --- | 0.04 | 14 | 0.0038 | 20 |
| 154 | GO:0043269 | P | regulation of ion transport |  |  |  |  |  |  | 0.015 | 5 | 0.00011 | 9 | 0.00083 | 9 | 0.0041 | 9 | 0.03 | 7 | 0.0036 | 10 |
| 155 | GO:0033365 | P | protein localization in organelle |  |  |  |  |  |  | 0.016 | 11 | 0.00045 | 18 | 0.004 | 19 | 0.037 | 19 | 0.021 | 19 | 0.0028 | 26 |
| 156 | GO:0034050 | P | host programmed cell death induced by symbiont |  |  |  |  |  |  | 0.016 | 9 | 0.003 | 13 | 0.00076 | 17 | --- | --- | --- | --- | 0.0021 | 21 |
| 157 | GO:0009626 | P | plant-type hypersensitive response |  |  |  |  |  |  | 0.016 | 9 | 0.003 | 13 | 0.00076 | 17 | --- | --- | --- | --- | 0.0021 | 21 |
| 158 | GO:0009251 | P | glucan catabolic process |  |  |  |  |  |  | 0.018 | 5 | 0.0037 | 7 | 0.016 | 7 | --- | --- | 0.00089 | 10 | 0.0016 | 11 |
| 159 | GO:0010876 | P | lipid localization |  |  |  |  |  |  | 0.019 | 6 | --- | --- | 0.0038 | 10 | 0.0064 | 11 | 0.00034 | 13 | 0.00011 | 16 |
| 160 | GO:0006534 | P | cysteine metabolic process |  |  |  |  |  |  | 0.019 | 6 | 4.8e-06 | 13 | 0.00032 | 12 | 0.0022 | 12 | 9.3e-05 | 14 | 3.1e-06 | 19 |
| 161 | GO:0009863 | P | salicylic acid mediated signaling pathway |  |  |  |  |  |  | 0.019 | 8 | 0.019 | 10 | 0.001 | 15 | --- | --- | --- | --- | 0.0016 | 19 |
| 162 | GO:0051187 | P | cofactor catabolic process |  |  |  |  |  |  | 0.019 | 8 | --- | --- | --- | --- | --- | --- | --- | --- | 0.0016 | 19 |
| 163 | GO:0009862 | P | systemic acquired resistance, salicylic acid mediated signaling pathway |  |  |  |  |  |  | 0.022 | 6 | 0.031 | 7 | 0.0048 | 10 | --- | --- | --- | --- | 0.0015 | 14 |
| 164 | GO:0010162 | P | seed dormancy |  |  |  |  |  |  | 0.022 | 5 | 0.0048 | 7 | 9.1e-05 | 11 | --- | --- | 0.00032 | 11 | 0.00068 | 12 |
| 165 | GO:0022611 | P | dormancy process |  |  |  |  |  |  | 0.022 | 5 | 0.0048 | 7 | 9.1e-05 | 11 | --- | --- | 0.00032 | 11 | 0.00068 | 12 |
| 166 | GO:0051186 | P | cofactor metabolic process |  |  |  |  |  |  | 0.023 | 25 | 2.1e-05 | 46 | 9.7e-08 | 64 | 3.2e-05 | 65 | 8.2e-10 | 78 | 4e-10 | 95 |
| 167 | GO:0032268 | P | regulation of cellular protein metabolic process |  |  |  |  |  |  | 0.024 | 8 | 0.00029 | 14 | 0.025 | 12 | --- | --- | 0.016 | 14 | 0.0024 | 19 |
| 168 | GO:0045454 | P | cell redox homeostasis |  |  |  |  |  |  | 0.024 | 10 | 0.0032 | 15 | 0.017 | 16 | 0.015 | 19 | --- | --- | 0.00034 | 27 |
| 169 | GO:0006631 | P | fatty acid metabolic process |  |  |  |  |  |  | 0.025 | 20 | 3e-06 | 40 | 6.7e-08 | 53 | 2.1e-05 | 53 | 2.8e-07 | 57 | 1.8e-06 | 66 |
| 170 | GO:0010053 | P | root epidermal cell differentiation |  |  |  |  |  |  | 0.026 | 9 | --- | --- | 0.00063 | 18 | 0.0031 | 19 | 0.018 | 16 | 0.00043 | 24 |
| 171 | GO:0006220 | P | pyrimidine nucleotide metabolic process |  |  |  |  |  |  | 0.027 | 5 | --- | --- | 0.00013 | 11 | 0.00022 | 12 | 0.0017 | 10 | 2.4e-05 | 15 |
| 172 | GO:0007059 | P | chromosome segregation |  |  |  |  |  |  | 0.03 | 6 | 1.3e-05 | 13 | 5.1e-05 | 14 | 0.00014 | 15 | 0.00025 | 14 | 4.4e-09 | 25 |
| 173 | GO:0048869 | P | cellular developmental process |  |  |  |  |  |  | 0.03 | 36 | --- | --- | --- | --- | --- | --- | --- | --- | --- | --- |
| 174 | GO:0009696 | P | salicylic acid metabolic process |  |  |  |  |  |  | 0.032 | 5 | 0.0079 | 7 | 0.00019 | 11 | --- | --- | 0.026 | 8 | 0.0015 | 12 |
| 175 | GO:0010431 | P | seed maturation |  |  |  |  |  |  | 0.032 | 5 | 0.0079 | 7 | 4.2e-05 | 12 | 0.039 | 8 | 0.00017 | 12 | 0.00013 | 14 |
| 176 | GO:0030154 | P | cell differentiation |  |  |  |  |  |  | 0.033 | 27 | --- | --- | 0.018 | 49 | --- | --- | --- | --- | --- | --- |
| 177 | GO:0006098 | P | pentose-phosphate shunt |  |  |  |  |  |  | 0.034 | 6 | 0.0012 | 10 | 1.7e-05 | 15 | 4.3e-06 | 18 | 1.8e-06 | 18 | 3.6e-07 | 22 |
| 178 | GO:0006306 | P | DNA methylation |  |  |  |  |  |  | 0.035 | 7 | 0.00082 | 12 | 0.0078 | 12 | --- | --- | 0.00051 | 16 | 5.1e-05 | 21 |
| 179 | GO:0006305 | P | DNA alkylation |  |  |  |  |  |  | 0.035 | 7 | 0.00082 | 12 | 0.0078 | 12 | --- | --- | 0.00051 | 16 | 5.1e-05 | 21 |
| 180 | GO:0006644 | P | phospholipid metabolic process |  |  |  |  |  |  | 0.037 | 13 | 0.0099 | 19 | 3.7e-06 | 33 | 9e-05 | 34 | 5.6e-06 | 36 | 5.8e-09 | 51 |
| 181 | GO:0005996 | P | monosaccharide metabolic process |  |  |  |  |  |  | 0.037 | 16 | 2.2e-06 | 34 | 7.5e-10 | 49 | 1.5e-10 | 58 | 9.3e-10 | 54 | 8.5e-13 | 72 |
| 182 | GO:0006740 | P | NADPH regeneration |  |  |  |  |  |  | 0.038 | 6 | 0.0015 | 10 | 2.3e-05 | 15 | 6e-06 | 18 | 2.5e-06 | 18 | 5.4e-07 | 22 |
| 183 | GO:0010054 | P | trichoblast differentiation |  |  |  |  |  |  | 0.038 | 8 | --- | --- | 0.00045 | 17 | 0.002 | 18 | 0.014 | 15 | 0.00019 | 23 |
| 184 | GO:0052542 | P | callose deposition during defense response |  |  |  |  |  |  | 0.038 | 5 | --- | --- | --- | --- | --- | --- | --- | --- | --- | --- |
| 185 | GO:0019220 | P | regulation of phosphate metabolic process |  |  |  |  |  |  | 0.038 | 8 | --- | --- | 0.02 | 13 | --- | --- | --- | --- | 0.013 | 18 |
| 186 | GO:0051174 | P | regulation of phosphorus metabolic process |  |  |  |  |  |  | 0.038 | 8 | --- | --- | 0.02 | 13 | --- | --- | --- | --- | 0.013 | 18 |
| 187 | GO:0007243 | P | protein kinase cascade |  |  |  |  |  |  | 0.038 | 5 | 0.037 | 6 | 0.013 | 8 | --- | --- | --- | --- | 0.017 | 10 |
| 188 | GO:0010467 | P | gene expression |  |  |  |  |  |  | 0.038 | 192 | --- | --- | 0.0017 | 401 | --- | --- | 0.0021 | 465 | 0.00017 | 614 |
| 189 | GO:0019222 | P | regulation of metabolic process |  |  |  |  |  |  | 0.04 | 115 | --- | --- | --- | --- | --- | --- | --- | --- | 0.02 | 340 |
| 190 | GO:0006304 | P | DNA modification |  |  |  |  |  |  | 0.041 | 7 | 0.00034 | 13 | 0.011 | 12 | --- | --- | 0.00028 | 17 | 9e-05 | 21 |
| 191 | GO:0043413 | P | macromolecule glycosylation |  |  |  |  |  |  | 0.041 | 9 | 0.028 | 12 | --- | --- | --- | --- | 0.037 | 16 | 0.0028 | 23 |
| 192 | GO:0006486 | P | protein amino acid glycosylation |  |  |  |  |  |  | 0.041 | 9 | 0.028 | 12 | --- | --- | --- | --- | 0.037 | 16 | 0.0028 | 23 |
| 193 | GO:0070085 | P | glycosylation |  |  |  |  |  |  | 0.041 | 9 | 0.028 | 12 | --- | --- | --- | --- | 0.037 | 16 | 0.0028 | 23 |
| 194 | GO:0009101 | P | glycoprotein biosynthetic process |  |  |  |  |  |  | 0.041 | 9 | 0.011 | 13 | --- | --- | --- | --- | 0.037 | 16 | 0.0013 | 24 |
| 195 | GO:0005982 | P | starch metabolic process |  |  |  |  |  |  | 0.041 | 7 | 9.2e-05 | 14 | 1.2e-05 | 18 | 0.025 | 13 | 8.8e-05 | 18 | 1.1e-05 | 23 |
| 196 | GO:0009933 | P | meristem structural organization |  |  |  |  |  |  | 0.041 | 8 | 2.1e-05 | 17 | 5.9e-06 | 21 | 0.0023 | 18 | 4.6e-08 | 27 | 7.2e-09 | 33 |
| 197 | GO:0048583 | P | regulation of response to stimulus |  |  |  |  |  |  | 0.042 | 17 | 0.0069 | 26 | 0.0043 | 33 | --- | --- | --- | --- | 0.038 | 41 |
| 198 | GO:0051049 | P | regulation of transport |  |  |  |  |  |  | 0.042 | 5 | 0.00077 | 9 | 0.0014 | 10 | 0.007 | 10 | 0.04 | 8 | 0.0028 | 12 |
| 199 | GO:0007049 | P | cell cycle |  |  |  |  |  |  | 0.043 | 22 | 3.6e-06 | 45 | 0.00017 | 49 | 0.0048 | 51 | 5.6e-05 | 57 | 4e-09 | 85 |
| 200 | GO:0009743 | P | response to carbohydrate stimulus |  |  |  |  |  |  | 0.043 | 20 | --- | --- | --- | --- | --- | --- | --- | --- | --- | --- |
| 201 | GO:0009069 | P | serine family amino acid metabolic process |  |  |  |  |  |  | 0.043 | 8 | 0.00092 | 14 | 0.0017 | 16 | 0.031 | 15 | 6.5e-05 | 21 | 0.00012 | 24 |
| 202 | GO:0009734 | P | auxin mediated signaling pathway |  |  |  |  |  |  | 0.045 | 7 | 0.0043 | 11 | 0.0017 | 14 | 0.028 | 13 | 0.0026 | 15 | 0.00012 | 21 |
| 203 | GO:0019318 | P | hexose metabolic process |  |  |  |  |  |  | 0.046 | 13 | 0.00025 | 24 | 6.1e-08 | 38 | 1.6e-09 | 47 | 1.3e-07 | 41 | 1.9e-10 | 56 |
| 204 | GO:0019725 | P | cellular homeostasis |  |  |  |  |  |  | 0.046 | 16 | --- | --- | --- | --- | 0.032 | 33 | --- | --- | 0.008 | 42 |
| 205 | GO:0048444 | P | floral organ morphogenesis |  |  |  |  |  |  | 0.046 | 6 | 0.025 | 8 | 0.015 | 10 | 0.011 | 12 | 0.0061 | 12 | 0.00012 | 18 |
| 206 | GO:0006366 | P | transcription from RNA polymerase II promoter |  |  |  |  |  |  | 0.048 | 5 | --- | --- | 0.0063 | 9 | 6.6e-05 | 14 | 0.0053 | 10 | 0.0004 | 14 |
| 207 | GO:0070838 | P | divalent metal ion transport |  |  |  |  |  |  | 0.048 | 5 | 0.015 | 7 | --- | --- | --- | --- | --- | --- | 0.011 | 11 |
| 208 | GO:0009156 | P | ribonucleoside monophosphate biosynthetic process |  |  |  |  |  |  | 0.048 | 5 | --- | --- | --- | --- | --- | --- | --- | --- | --- | --- |
| 209 | GO:0003824 | F | catalytic activity |  |  |  |  |  |  | 2.1e-45 | 643 | 2.5e-66 | 1013 | 6.9e-86 | 1341 | 8.1e-104 | 1643 | 1e-103 | 1595 | 1.1e-132 | 2095 |
| 210 | GO:0046872 | F | metal ion binding |  |  |  |  |  |  | 2.1e-40 | 226 | 2.5e-66 | 369 | 2.4e-80 | 476 | 1.4e-88 | 565 | 6.7e-93 | 561 | 1e-108 | 715 |
| 211 | GO:0043167 | F | ion binding |  |  |  |  |  |  | 2.1e-38 | 227 | 3.6e-63 | 371 | 1.8e-75 | 477 | 2.2e-83 | 567 | 2.7e-87 | 562 | 1.5e-102 | 718 |
| 212 | GO:0043169 | F | cation binding |  |  |  |  |  |  | 2.1e-38 | 227 | 3.6e-63 | 371 | 1.8e-75 | 477 | 2.2e-83 | 567 | 2.7e-87 | 562 | 1.5e-102 | 718 |
| 213 | GO:0032559 | F | adenyl ribonucleotide binding |  |  |  |  |  |  | 2.4e-35 | 173 | 4.5e-62 | 291 | 9.3e-86 | 398 | 5.3e-108 | 499 | 5.6e-98 | 467 | 4.4e-123 | 612 |
| 214 | GO:0030554 | F | adenyl nucleotide binding |  |  |  |  |  |  | 1.1e-34 | 177 | 8.2e-63 | 302 | 3.9e-89 | 418 | 4e-111 | 521 | 2.1e-103 | 493 | 4.9e-128 | 642 |
| 215 | GO:0001883 | F | purine nucleoside binding |  |  |  |  |  |  | 1.1e-34 | 177 | 8.2e-63 | 302 | 3.9e-89 | 418 | 4e-111 | 521 | 2.1e-103 | 493 | 4.9e-128 | 642 |
| 216 | GO:0001882 | F | nucleoside binding |  |  |  |  |  |  | 1.1e-34 | 177 | 8.2e-63 | 302 | 3.7e-89 | 419 | 3.9e-111 | 522 | 2.1e-103 | 493 | 4.9e-128 | 642 |
| 217 | GO:0000166 | F | nucleotide binding |  |  |  |  |  |  | 1.1e-33 | 227 | 9.5e-61 | 383 | 9.1e-86 | 526 | 1.2e-100 | 639 | 1.2e-97 | 616 | 1.1e-119 | 799 |
| 218 | GO:0032555 | F | purine ribonucleotide binding |  |  |  |  |  |  | 8.9e-33 | 184 | 1.1e-61 | 318 | 9.4e-83 | 429 | 9.2e-100 | 527 | 2.4e-91 | 496 | 1.2e-115 | 652 |
| 219 | GO:0032553 | F | ribonucleotide binding |  |  |  |  |  |  | 8.9e-33 | 184 | 1.1e-61 | 318 | 9.4e-83 | 429 | 9.2e-100 | 527 | 2.4e-91 | 496 | 1.2e-115 | 652 |
| 220 | GO:0017076 | F | purine nucleotide binding |  |  |  |  |  |  | 3.7e-32 | 188 | 1.6e-62 | 329 | 3.6e-86 | 449 | 6.1e-103 | 549 | 1.2e-96 | 522 | 1.5e-120 | 682 |
| 221 | GO:0005524 | F | ATP binding |  |  |  |  |  |  | 4.4e-30 | 161 | 9.7e-53 | 271 | 1.4e-68 | 361 | 2.3e-88 | 456 | 1.7e-80 | 428 | 3.7e-104 | 568 |
| 222 | GO:0005488 | F | binding |  |  |  |  |  |  | 3.2e-29 | 657 | 1.5e-48 | 1063 | 8.6e-68 | 1429 | 1.3e-77 | 1734 | 1.8e-75 | 1671 | 5e-94 | 2185 |
| 223 | GO:0046914 | F | transition metal ion binding |  |  |  |  |  |  | 2.5e-20 | 154 | 2.5e-34 | 254 | 1e-38 | 322 | 6.6e-44 | 387 | 3.4e-47 | 386 | 2e-50 | 479 |
| 224 | GO:0004672 | F | protein kinase activity |  |  |  |  |  |  | 6.4e-19 | 108 | 5.2e-18 | 145 | 5.6e-20 | 183 | 2.7e-28 | 237 | 1.5e-30 | 237 | 2.9e-40 | 316 |
| 225 | GO:0016740 | F | transferase activity |  |  |  |  |  |  | 1.4e-18 | 244 | 1.1e-15 | 339 | 7.9e-19 | 443 | 1.1e-17 | 517 | 5.1e-25 | 537 | 5e-33 | 711 |
| 226 | GO:0016773 | F | phosphotransferase activity, alcohol group as acceptor |  |  |  |  |  |  | 3.7e-17 | 117 | 3.4e-16 | 159 | 5.8e-20 | 208 | 3.6e-27 | 265 | 3.2e-28 | 261 | 2.4e-38 | 351 |
| 227 | GO:0016787 | F | hydrolase activity |  |  |  |  |  |  | 3e-13 | 232 | 4.8e-26 | 393 | 2e-35 | 528 | 5.6e-53 | 684 | 3.9e-42 | 626 | 4.3e-55 | 828 |
| 228 | GO:0008270 | F | zinc ion binding |  |  |  |  |  |  | 1.2e-12 | 114 | 2.7e-18 | 179 | 2.9e-21 | 230 | 2.8e-26 | 284 | 4.1e-24 | 269 | 1.8e-28 | 346 |
| 229 | GO:0043565 | F | sequence-specific DNA binding |  |  |  |  |  |  | 8.4e-12 | 27 | 5.7e-15 | 39 | 1.7e-22 | 57 | 7e-33 | 79 | 3.6e-31 | 75 | --- | --- |
| 230 | GO:0005506 | F | iron ion binding |  |  |  |  |  |  | 1.7e-09 | 30 | 2.1e-19 | 56 | 2e-28 | 80 | 9.1e-28 | 88 | 5e-35 | 98 | 6.7e-37 | 117 |
| 231 | GO:0016788 | F | hydrolase activity, acting on ester bonds |  |  |  |  |  |  | 3e-09 | 98 | 1.1e-09 | 141 | 5.3e-18 | 207 | 2.4e-30 | 282 | 9.8e-20 | 240 | 2.6e-26 | 320 |
| 232 | GO:0016772 | F | transferase activity, transferring phosphorus-containing groups |  |  |  |  |  |  | 7e-09 | 134 | 3.4e-06 | 180 | 3.2e-08 | 241 | 4e-11 | 302 | 1.4e-12 | 300 | 2.8e-18 | 406 |
| 233 | GO:0016301 | F | kinase activity |  |  |  |  |  |  | 1.3e-08 | 120 | 2.2e-06 | 162 | 1.4e-07 | 212 | 3.4e-11 | 271 | 6.8e-12 | 266 | 5.4e-17 | 359 |
| 234 | GO:0020037 | F | heme binding |  |  |  |  |  |  | 1.2e-07 | 21 | 7.2e-16 | 40 | 3.6e-22 | 56 | 5.4e-26 | 68 | 1.5e-29 | 72 | 1.8e-34 | 91 |
| 235 | GO:0003678 | F | DNA helicase activity |  |  |  |  |  |  | 9.1e-07 | 10 | --- | --- | --- | --- | --- | --- | --- | --- | --- | --- |
| 236 | GO:0046906 | F | tetrapyrrole binding |  |  |  |  |  |  | 1.2e-06 | 23 | 6.7e-12 | 40 | 6.7e-20 | 61 | 1.1e-22 | 73 | 8.8e-25 | 75 | 1.6e-29 | 96 |
| 237 | GO:0016491 | F | oxidoreductase activity |  |  |  |  |  |  | 4e-06 | 101 | 1.4e-07 | 154 | 3.4e-11 | 211 | 7.8e-12 | 251 | 3.2e-14 | 254 | 7.4e-15 | 319 |
| 238 | GO:0008146 | F | sulfotransferase activity |  |  |  |  |  |  | 2.4e-05 | 9 | --- | --- | 0.00031 | 11 | 2.9e-07 | 17 | 0.0012 | 11 | 0.001 | 13 |
| 239 | GO:0016782 | F | transferase activity, transferring sulfur-containing groups |  |  |  |  |  |  | 2.5e-05 | 11 | --- | --- | 0.0001 | 15 | 2.2e-05 | 18 | 0.0018 | 14 | 0.0032 | 16 |
| 240 | GO:0016746 | F | transferase activity, transferring acyl groups |  |  |  |  |  |  | 2.8e-05 | 34 | --- | --- | 0.0014 | 51 | --- | --- | 0.00078 | 59 | 0.00012 | 77 |
| 241 | GO:0048037 | F | cofactor binding |  |  |  |  |  |  | 5.8e-05 | 28 | 2.7e-09 | 49 | 3.6e-14 | 70 | 1.1e-15 | 83 | 3.6e-16 | 82 | 1e-18 | 104 |
| 242 | GO:0003676 | F | nucleic acid binding |  |  |  |  |  |  | 9.3e-05 | 252 | 7.1e-05 | 384 | 5.4e-10 | 541 | 1.2e-12 | 667 | 2.9e-10 | 627 | 1.7e-14 | 835 |
| 243 | GO:0008168 | F | methyltransferase activity |  |  |  |  |  |  | 0.00013 | 29 | 8.5e-07 | 46 | 3.2e-06 | 54 | 5.5e-05 | 58 | 2e-05 | 58 | 1.7e-08 | 82 |
| 244 | GO:0016741 | F | transferase activity, transferring one-carbon groups |  |  |  |  |  |  | 0.00014 | 29 | 1e-06 | 46 | 1.9e-06 | 55 | 6.5e-05 | 58 | 1.3e-05 | 59 | 2.3e-08 | 82 |
| 245 | GO:0016278 | F | lysine N-methyltransferase activity |  |  |  |  |  |  | 0.00016 | 6 | 0.013 | 5 | 0.0077 | 6 | --- | --- | 0.018 | 6 | --- | --- |
| 246 | GO:0016279 | F | protein-lysine N-methyltransferase activity |  |  |  |  |  |  | 0.00016 | 6 | 0.013 | 5 | 0.0077 | 6 | --- | --- | 0.018 | 6 | --- | --- |
| 247 | GO:0050662 | F | coenzyme binding |  |  |  |  |  |  | 0.00017 | 23 | 3.2e-07 | 38 | 6.4e-10 | 52 | 2.7e-10 | 60 | 1.2e-09 | 57 | 5.6e-13 | 77 |
| 248 | GO:0070011 | F | peptidase activity, acting on L-amino acid peptides |  |  |  |  |  |  | 0.00036 | 46 | 9.8e-10 | 85 | 1.8e-09 | 103 | 3.6e-10 | 122 | 5.6e-13 | 128 | 9.8e-11 | 148 |
| 249 | GO:0004175 | F | endopeptidase activity |  |  |  |  |  |  | 0.0007 | 26 | 1.1e-06 | 44 | 0.0015 | 43 | 0.024 | 45 | 1.6e-05 | 56 | 6.1e-06 | 70 |
| 250 | GO:0016747 | F | transferase activity, transferring acyl groups other than amino-acyl groups |  |  |  |  |  |  | 0.00075 | 28 | --- | --- | --- | --- | --- | --- | --- | --- | 0.022 | 60 |
| 251 | GO:0003677 | F | DNA binding |  |  |  |  |  |  | 0.00096 | 157 | --- | --- | 7.3e-08 | 339 | 1.9e-08 | 408 | 9.6e-07 | 382 | 4.2e-10 | 514 |
| 252 | GO:0030246 | F | carbohydrate binding |  |  |  |  |  |  | 0.0013 | 19 | 8.5e-05 | 29 | 0.00019 | 34 | --- | --- | 9.7e-05 | 39 | 1.8e-06 | 53 |
| 253 | GO:0018024 | F | histone-lysine N-methyltransferase activity |  |  |  |  |  |  | 0.0016 | 5 | 0.013 | 5 | 0.037 | 5 | --- | --- | --- | --- | --- | --- |
| 254 | GO:0016866 | F | intramolecular transferase activity |  |  |  |  |  |  | 0.0016 | 11 | --- | --- | 0.00022 | 19 | --- | --- | --- | --- | 0.016 | 20 |
| 255 | GO:0005215 | F | transporter activity |  |  |  |  |  |  | 0.0018 | 89 | --- | --- | 0.0073 | 164 | --- | --- | --- | --- | 0.016 | 239 |
| 256 | GO:0008233 | F | peptidase activity |  |  |  |  |  |  | 0.0018 | 47 | 5.1e-08 | 86 | 9.2e-08 | 105 | 2.1e-08 | 125 | 2.1e-10 | 129 | 1.4e-08 | 151 |
| 257 | GO:0004553 | F | hydrolase activity, hydrolyzing O-glycosyl compounds |  |  |  |  |  |  | 0.0057 | 36 | 1.8e-06 | 65 | 0.00019 | 72 | 0.0085 | 76 | 8e-07 | 92 | 5.9e-07 | 114 |
| 258 | GO:0016758 | F | transferase activity, transferring hexosyl groups |  |  |  |  |  |  | 0.0074 | 28 | 1.4e-07 | 55 | 0.00072 | 54 | 0.00027 | 65 | 9.1e-06 | 69 | 4.3e-06 | 86 |
| 259 | GO:0051287 | F | NAD or NADH binding |  |  |  |  |  |  | 0.0074 | 7 | 0.027 | 8 | 0.0001 | 14 | 1.6e-05 | 17 | 3.9e-05 | 16 | 7e-07 | 22 |
| 260 | GO:0016798 | F | hydrolase activity, acting on glycosyl bonds |  |  |  |  |  |  | 0.016 | 37 | 5e-06 | 68 | 0.0016 | 73 | --- | --- | 1.2e-05 | 94 | 5.9e-06 | 118 |
| 261 | GO:0015035 | F | protein disulfide oxidoreductase activity |  |  |  |  |  |  | 0.016 | 8 | --- | --- | --- | --- | --- | --- | --- | --- | 0.014 | 17 |
| 262 | GO:0016616 | F | oxidoreductase activity, acting on the CH-OH group of donors, NAD or NADP as acceptor |  |  |  |  |  |  | 0.019 | 15 | 0.017 | 21 | 0.013 | 26 | 0.0026 | 33 | 0.0094 | 30 | 0.0015 | 40 |
| 263 | GO:0008170 | F | N-methyltransferase activity |  |  |  |  |  |  | 0.026 | 6 | --- | --- | --- | --- | --- | --- | --- | --- | 0.012 | 13 |
| 264 | GO:0042054 | F | histone methyltransferase activity |  |  |  |  |  |  | 0.029 | 5 | --- | --- | --- | --- | --- | --- | --- | --- | --- | --- |
| 265 | GO:0016667 | F | oxidoreductase activity, acting on sulfur group of donors |  |  |  |  |  |  | 0.034 | 12 | --- | --- | --- | --- | --- | --- | --- | --- | 0.0059 | 31 |
| 266 | GO:0016614 | F | oxidoreductase activity, acting on CH-OH group of donors |  |  |  |  |  |  | 0.034 | 17 | 0.0042 | 27 | 0.015 | 31 | 0.0034 | 39 | 0.0029 | 38 | 0.00025 | 51 |
| 267 | GO:0019842 | F | vitamin binding |  |  |  |  |  |  | 0.035 | 8 | --- | --- | 1.5e-06 | 22 | 9e-07 | 25 | 1.4e-07 | 26 | 2.3e-09 | 34 |
| 268 | GO:0008276 | F | protein methyltransferase activity |  |  |  |  |  |  | 0.043 | 6 | --- | --- | --- | --- | --- | --- | --- | --- | --- | --- |
| 269 | GO:0031224 | C | intrinsic to membrane |  |  |  |  |  |  | 0 | 450 | 0 | 646 | --- | --- | --- | --- | --- | --- | --- | --- |
| 270 | GO:0044425 | C | membrane part |  |  |  |  |  |  | 3.4e-263 | 466 | 0 | 682 | 0 | 958 | 0 | 1141 | 0 | 1100 | --- | --- |
| 271 | GO:0016020 | C | membrane |  |  |  |  |  |  | 5.4e-114 | 499 | 6.2e-155 | 759 | 1e-214 | 1035 | 5.1e-243 | 1240 | 3.1e-236 | 1196 | 4.7e-305 | 1583 |
| 272 | GO:0005623 | C | cell |  |  |  |  |  |  | 1e-21 | 792 | 2.6e-27 | 1235 | 3e-43 | 1678 | 1.9e-40 | 1994 | 1.8e-46 | 1958 | 1e-62 | 2588 |
| 273 | GO:0044464 | C | cell part |  |  |  |  |  |  | 5.3e-19 | 779 | 1.1e-25 | 1226 | 7.4e-40 | 1661 | 8.1e-37 | 1973 | 7.7e-44 | 1944 | 3.3e-57 | 2559 |
| 274 | GO:0055044 | C | symplast |  |  |  |  |  |  | 3.3e-09 | 13 | --- | --- | --- | --- | 8.8e-08 | 18 | --- | --- | --- | --- |
| 275 | GO:0009506 | C | plasmodesma |  |  |  |  |  |  | 3.3e-09 | 13 | --- | --- | --- | --- | 8.8e-08 | 18 | --- | --- | --- | --- |
| 276 | GO:0005911 | C | cell-cell junction |  |  |  |  |  |  | 7.2e-09 | 13 | --- | --- | 5.5e-11 | 21 | 2.2e-07 | 18 | --- | --- | --- | --- |
| 277 | GO:0030054 | C | cell junction |  |  |  |  |  |  | 1.5e-08 | 13 | 8.8e-14 | 22 | 1.5e-10 | 21 | 5.3e-07 | 18 | --- | --- | --- | --- |
| 278 | GO:0005634 | C | nucleus |  |  |  |  |  |  | 0.00035 | 149 | 0.013 | 212 | 0.00048 | 287 | 0.003 | 335 | 1.9e-05 | 345 | 8.9e-09 | 467 |
| 279 | GO:0009308 | P | amine metabolic process |  |  |  |  |  |  | --- | --- | 2.6e-11 | 85 | 1e-05 | 83 | 0.002 | 86 | 1e-09 | 110 | 1e-08 | 130 |
| 280 | GO:0044106 | P | cellular amine metabolic process |  |  |  |  |  |  | --- | --- | 3.1e-11 | 76 | 3.1e-07 | 79 | 0.00033 | 79 | 4.5e-10 | 99 | 2.8e-10 | 121 |
| 281 | GO:0006520 | P | cellular amino acid metabolic process |  |  |  |  |  |  | --- | --- | 5.4e-09 | 69 | 3.8e-06 | 74 | 0.0063 | 71 | 3.8e-08 | 91 | 1.2e-07 | 109 |
| 282 | GO:0009309 | P | amine biosynthetic process |  |  |  |  |  |  | --- | --- | 1.8e-08 | 46 | 0.00071 | 41 | --- | --- | 2.1e-06 | 55 | 0.00011 | 60 |
| 283 | GO:0022607 | P | cellular component assembly |  |  |  |  |  |  | --- | --- | 1.9e-08 | 50 | 4.8e-06 | 53 | 4.1e-07 | 65 | 1.6e-10 | 73 | 9.6e-10 | 85 |
| 284 | GO:0009664 | P | plant-type cell wall organization |  |  |  |  |  |  | --- | --- | 3.1e-08 | 26 | 0.0078 | 18 | 0.00033 | 25 | 0.00014 | 25 | 0.0096 | 24 |
| 285 | GO:0000097 | P | sulfur amino acid biosynthetic process |  |  |  |  |  |  | --- | --- | 3.2e-08 | 21 | 2.5e-05 | 19 | 0.0029 | 17 | 1.4e-08 | 27 | 5.7e-09 | 32 |
| 286 | GO:0044272 | P | sulfur compound biosynthetic process |  |  |  |  |  |  | --- | --- | 3.9e-08 | 31 | 0.00035 | 27 | 0.025 | 25 | 1.7e-08 | 41 | 1.4e-07 | 46 |
| 287 | GO:0006066 | P | alcohol metabolic process |  |  |  |  |  |  | --- | --- | 8.1e-08 | 49 | 1.7e-08 | 61 | 1.1e-09 | 74 | 7.2e-10 | 72 | 4.1e-14 | 99 |
| 288 | GO:0000096 | P | sulfur amino acid metabolic process |  |  |  |  |  |  | --- | --- | 8.1e-08 | 26 | 1e-05 | 26 | 0.0017 | 24 | 3.8e-08 | 34 | 1.8e-09 | 43 |
| 289 | GO:0008652 | P | cellular amino acid biosynthetic process |  |  |  |  |  |  | --- | --- | 1.1e-07 | 41 | 0.00055 | 38 | --- | --- | 1.7e-06 | 51 | 0.0001 | 55 |
| 290 | GO:0006310 | P | DNA recombination |  |  |  |  |  |  | --- | --- | 1.9e-07 | 21 | 3.5e-05 | 20 | 9.8e-06 | 24 | 1.2e-06 | 25 | 6e-08 | 32 |
| 291 | GO:0044085 | P | cellular component biogenesis |  |  |  |  |  |  | --- | --- | 1.9e-07 | 78 | 7.1e-05 | 85 | 4.8e-06 | 106 | 1.8e-10 | 120 | 5.7e-09 | 140 |
| 292 | GO:0022402 | P | cell cycle process |  |  |  |  |  |  | --- | --- | 5.8e-07 | 33 | 0.00029 | 32 | 0.0079 | 32 | 0.00011 | 37 | 4.2e-09 | 58 |
| 293 | GO:0000279 | P | M phase |  |  |  |  |  |  | --- | --- | 5.8e-07 | 23 | 0.0045 | 18 | 0.0044 | 21 | 0.00091 | 22 | 2.8e-06 | 33 |
| 294 | GO:0010383 | P | cell wall polysaccharide metabolic process |  |  |  |  |  |  | --- | --- | 5.8e-07 | 12 | 2.8e-07 | 14 | --- | --- | --- | --- | --- | --- |
| 295 | GO:0016568 | P | chromatin modification |  |  |  |  |  |  | --- | --- | 5.8e-07 | 26 | 2.2e-05 | 27 | 5.3e-05 | 30 | 4.9e-08 | 36 | 7.2e-08 | 42 |
| 296 | GO:0044036 | P | cell wall macromolecule metabolic process |  |  |  |  |  |  | --- | --- | 1e-06 | 18 | 0.001 | 15 | 5e-07 | 24 | 6.8e-07 | 23 | 4.6e-09 | 31 |
| 297 | GO:0022403 | P | cell cycle phase |  |  |  |  |  |  | --- | --- | 1.4e-06 | 25 | 0.016 | 19 | 0.02 | 22 | 0.0023 | 24 | 1e-05 | 36 |
| 298 | GO:0006519 | P | cellular amino acid and derivative metabolic process |  |  |  |  |  |  | --- | --- | 1.4e-06 | 85 | 0.00045 | 93 | 0.037 | 97 | 2.6e-05 | 113 | 2e-06 | 147 |
| 299 | GO:0034968 | P | histone lysine methylation |  |  |  |  |  |  | --- | --- | 2.2e-06 | 11 | 0.0095 | 7 | 0.00072 | 10 | 1.9e-05 | 12 | --- | --- |
| 300 | GO:0009240 | P | isopentenyl diphosphate biosynthetic process |  |  |  |  |  |  | --- | --- | 2.2e-06 | 12 | 0.00056 | 10 | 0.0032 | 10 | 6.1e-06 | 14 | 6.4e-06 | 16 |
| 301 | GO:0019288 | P | isopentenyl diphosphate biosynthetic process, mevalonate-independent pathway |  |  |  |  |  |  | --- | --- | 2.2e-06 | 11 | 0.00013 | 10 | 0.00072 | 10 | 4e-06 | 13 | --- | --- |
| 302 | GO:0046490 | P | isopentenyl diphosphate metabolic process |  |  |  |  |  |  | --- | --- | 2.2e-06 | 12 | 0.00056 | 10 | 0.0032 | 10 | 6.1e-06 | 14 | 6.4e-06 | 16 |
| 303 | GO:0019682 | P | glyceraldehyde-3-phosphate metabolic process |  |  |  |  |  |  | --- | --- | 2.2e-06 | 11 | 0.00013 | 10 | 0.00072 | 10 | 4e-06 | 13 | --- | --- |
| 304 | GO:0042546 | P | cell wall biogenesis |  |  |  |  |  |  | --- | --- | 3.3e-06 | 21 | 5.7e-05 | 22 | 4.2e-09 | 34 | 3.4e-06 | 27 | 1.4e-07 | 35 |
| 305 | GO:0044271 | P | cellular nitrogen compound biosynthetic process |  |  |  |  |  |  | --- | --- | 3.3e-06 | 57 | 0.0023 | 58 | --- | --- | 1.5e-05 | 76 | 0.00069 | 85 |
| 306 | GO:0045892 | P | negative regulation of transcription, DNA-dependent |  |  |  |  |  |  | --- | --- | 3.7e-06 | 16 | 5.4e-07 | 20 | 3.5e-06 | 21 | 9.8e-08 | 23 | 1.6e-08 | 28 |
| 307 | GO:0051253 | P | negative regulation of RNA metabolic process |  |  |  |  |  |  | --- | --- | 3.7e-06 | 16 | 5.4e-07 | 20 | 3.5e-06 | 21 | 9.8e-08 | 23 | 1.6e-08 | 28 |
| 308 | GO:0006790 | P | sulfur metabolic process |  |  |  |  |  |  | --- | --- | 4.1e-06 | 39 | 0.0039 | 37 | 0.037 | 39 | 6.5e-06 | 52 | 2.3e-07 | 68 |
| 309 | GO:0006325 | P | chromatin organization |  |  |  |  |  |  | --- | --- | 4.1e-06 | 34 | 0.0004 | 35 | 0.0013 | 39 | 1.3e-05 | 44 | 2.6e-05 | 52 |
| 310 | GO:0009070 | P | serine family amino acid biosynthetic process |  |  |  |  |  |  | --- | --- | 4.8e-06 | 14 | 0.0011 | 12 | 0.007 | 12 | 3.4e-05 | 16 | 6.4e-06 | 20 |
| 311 | GO:0019344 | P | cysteine biosynthetic process |  |  |  |  |  |  | --- | --- | 4.8e-06 | 13 | 0.0011 | 11 | 0.0064 | 11 | 9.3e-05 | 14 | 1e-05 | 18 |
| 312 | GO:0044255 | P | cellular lipid metabolic process |  |  |  |  |  |  | --- | --- | 4.9e-06 | 77 | 7.4e-07 | 99 | 5.7e-05 | 107 | 2.6e-07 | 114 | 1.1e-08 | 147 |
| 313 | GO:0016571 | P | histone methylation |  |  |  |  |  |  | --- | --- | 6.3e-06 | 14 | 0.013 | 10 | 0.0001 | 16 | 4.8e-05 | 16 | 9.1e-06 | 20 |
| 314 | GO:0046185 | P | aldehyde catabolic process |  |  |  |  |  |  | --- | --- | 1.2e-05 | 9 | 0.044 | 5 | --- | --- | 0.00028 | 9 | --- | --- |
| 315 | GO:0016569 | P | covalent chromatin modification |  |  |  |  |  |  | --- | --- | 1.3e-05 | 20 | 0.0031 | 18 | 0.00021 | 24 | 1.2e-05 | 26 | 3.6e-06 | 32 |
| 316 | GO:0070646 | P | protein modification by small protein removal |  |  |  |  |  |  | --- | --- | 1.3e-05 | 12 | 3.4e-09 | 19 | --- | --- | 3.1e-10 | 22 | --- | --- |
| 317 | GO:0016570 | P | histone modification |  |  |  |  |  |  | --- | --- | 1.9e-05 | 19 | 0.044 | 14 | 0.0032 | 20 | 9.2e-05 | 23 | 4.4e-05 | 28 |
| 318 | GO:0006479 | P | protein amino acid methylation |  |  |  |  |  |  | --- | --- | 1.9e-05 | 14 | 0.0011 | 13 | 8.6e-06 | 19 | 1.3e-05 | 18 | 3.7e-06 | 22 |
| 319 | GO:0008213 | P | protein amino acid alkylation |  |  |  |  |  |  | --- | --- | 1.9e-05 | 14 | 0.0011 | 13 | 8.6e-06 | 19 | 1.3e-05 | 18 | 3.7e-06 | 22 |
| 320 | GO:0019252 | P | starch biosynthetic process |  |  |  |  |  |  | --- | --- | 2.5e-05 | 11 | 6.1e-07 | 15 | 0.0055 | 10 | 3.4e-06 | 15 | 1.2e-06 | 18 |
| 321 | GO:0010014 | P | meristem initiation |  |  |  |  |  |  | --- | --- | 3.2e-05 | 9 | --- | --- | --- | --- | --- | --- | --- | --- |
| 322 | GO:0007126 | P | meiosis |  |  |  |  |  |  | --- | --- | 3.7e-05 | 15 | --- | --- | --- | --- | 0.02 | 13 | 0.0004 | 20 |
| 323 | GO:0051327 | P | M phase of meiotic cell cycle |  |  |  |  |  |  | --- | --- | 3.7e-05 | 15 | --- | --- | --- | --- | 0.02 | 13 | 0.0004 | 20 |
| 324 | GO:0048532 | P | anatomical structure arrangement |  |  |  |  |  |  | --- | --- | 4.3e-05 | 17 | 1.3e-05 | 21 | 0.0044 | 18 | 1.4e-07 | 27 | 2.6e-08 | 33 |
| 325 | GO:0070592 | P | cell wall polysaccharide biosynthetic process |  |  |  |  |  |  | --- | --- | 4.7e-05 | 8 | --- | --- | --- | --- | --- | --- | --- | --- |
| 326 | GO:0045492 | P | xylan biosynthetic process |  |  |  |  |  |  | --- | --- | 4.7e-05 | 8 | --- | --- | --- | --- | --- | --- | --- | --- |
| 327 | GO:0065003 | P | macromolecular complex assembly |  |  |  |  |  |  | --- | --- | 4.7e-05 | 35 | 0.0019 | 37 | 0.0027 | 43 | 2e-07 | 55 | 3.6e-06 | 62 |
| 328 | GO:0000226 | P | microtubule cytoskeleton organization |  |  |  |  |  |  | --- | --- | 4.9e-05 | 16 | --- | --- | --- | --- | 0.012 | 15 | 0.0022 | 20 |
| 329 | GO:0006396 | P | RNA processing |  |  |  |  |  |  | --- | --- | 6.1e-05 | 58 | 3.4e-06 | 77 | 0.0013 | 78 | 7.2e-09 | 97 | 7.5e-08 | 114 |
| 330 | GO:0043933 | P | macromolecular complex subunit organization |  |  |  |  |  |  | --- | --- | 7.6e-05 | 37 | 0.0061 | 38 | 0.0022 | 47 | 9.8e-07 | 57 | 1.1e-05 | 65 |
| 331 | GO:0019243 | P | methylglyoxal catabolic process to D-lactate |  |  |  |  |  |  | --- | --- | 7.7e-05 | 8 | 0.044 | 5 | --- | --- | 0.0013 | 8 | 0.0015 | 9 |
| 332 | GO:0006089 | P | lactate metabolic process |  |  |  |  |  |  | --- | --- | 7.7e-05 | 8 | 0.044 | 5 | --- | --- | 0.0013 | 8 | 0.0015 | 9 |
| 333 | GO:0009438 | P | methylglyoxal metabolic process |  |  |  |  |  |  | --- | --- | 7.7e-05 | 8 | 0.044 | 5 | --- | --- | 0.0013 | 8 | 0.0015 | 9 |
| 334 | GO:0051596 | P | methylglyoxal catabolic process |  |  |  |  |  |  | --- | --- | 7.7e-05 | 8 | 0.044 | 5 | --- | --- | 0.0013 | 8 | 0.0015 | 9 |
| 335 | GO:0006308 | P | DNA catabolic process |  |  |  |  |  |  | --- | --- | 0.00012 | 8 | 0.015 | 6 | 0.046 | 6 | 0.002 | 8 | --- | --- |
| 336 | GO:0045491 | P | xylan metabolic process |  |  |  |  |  |  | --- | --- | 0.00012 | 8 | --- | --- | --- | --- | --- | --- | --- | --- |
| 337 | GO:0002252 | P | immune effector process |  |  |  |  |  |  | --- | --- | 0.00016 | 12 | 0.0017 | 12 | 0.028 | 11 | 4.9e-06 | 18 | 1.2e-05 | 20 |
| 338 | GO:0006399 | P | tRNA metabolic process |  |  |  |  |  |  | --- | --- | 0.00019 | 26 | 0.00027 | 31 | 0.023 | 29 | 9.2e-05 | 36 | 0.0027 | 38 |
| 339 | GO:0044038 | P | cell wall macromolecule biosynthetic process |  |  |  |  |  |  | --- | --- | 0.0002 | 8 | 9e-06 | 11 | --- | --- | --- | --- | --- | --- |
| 340 | GO:0070589 | P | cellular component macromolecule biosynthetic process |  |  |  |  |  |  | --- | --- | 0.0002 | 8 | 9e-06 | 11 | --- | --- | --- | --- | --- | --- |
| 341 | GO:0009451 | P | RNA modification |  |  |  |  |  |  | --- | --- | 0.00021 | 25 | 0.016 | 24 | --- | --- | --- | --- | --- | --- |
| 342 | GO:0042278 | P | purine nucleoside metabolic process |  |  |  |  |  |  | --- | --- | 0.00023 | 10 | 0.0063 | 9 | --- | --- | 0.0016 | 11 | 0.0038 | 12 |
| 343 | GO:0046128 | P | purine ribonucleoside metabolic process |  |  |  |  |  |  | --- | --- | 0.00023 | 10 | 0.0063 | 9 | --- | --- | 0.0016 | 11 | 0.0038 | 12 |
| 344 | GO:0035196 | P | production of miRNAs involved in gene silencing by miRNA |  |  |  |  |  |  | --- | --- | 0.00023 | 9 | 0.0004 | 10 | 0.00015 | 12 | 8.6e-07 | 15 | --- | --- |
| 345 | GO:0034622 | P | cellular macromolecular complex assembly |  |  |  |  |  |  | --- | --- | 0.00023 | 32 | --- | --- | --- | --- | 0.00074 | 42 | 0.0039 | 48 |
| 346 | GO:0022618 | P | ribonucleoprotein complex assembly |  |  |  |  |  |  | --- | --- | 0.00023 | 9 | --- | --- | --- | --- | 0.0013 | 10 | 0.00068 | 12 |
| 347 | GO:0006302 | P | double-strand break repair |  |  |  |  |  |  | --- | --- | 0.00025 | 11 | 0.021 | 9 | --- | --- | 0.0025 | 12 | 0.0003 | 16 |
| 348 | GO:0007010 | P | cytoskeleton organization |  |  |  |  |  |  | --- | --- | 0.00027 | 26 | 0.0065 | 27 | 0.0032 | 33 | 0.0013 | 33 | 2.6e-05 | 46 |
| 349 | GO:0009627 | P | systemic acquired resistance |  |  |  |  |  |  | --- | --- | 0.00029 | 15 | 0.00023 | 18 | --- | --- | 0.04 | 14 | 4.7e-05 | 25 |
| 350 | GO:0034660 | P | ncRNA metabolic process |  |  |  |  |  |  | --- | --- | 0.0003 | 37 | 0.0004 | 45 | 0.047 | 43 | 7.2e-06 | 57 | 0.011 | 55 |
| 351 | GO:0010413 | P | glucuronoxylan metabolic process |  |  |  |  |  |  | --- | --- | 0.00031 | 7 | --- | --- | --- | --- | --- | --- | --- | --- |
| 352 | GO:0034621 | P | cellular macromolecular complex subunit organization |  |  |  |  |  |  | --- | --- | 0.00031 | 34 | --- | --- | --- | --- | 0.0028 | 43 | 0.011 | 50 |
| 353 | GO:0016071 | P | mRNA metabolic process |  |  |  |  |  |  | --- | --- | 0.00037 | 25 | 0.046 | 23 | 0.00085 | 34 | 0.0048 | 30 | 2.4e-06 | 48 |
| 354 | GO:0048507 | P | meristem development |  |  |  |  |  |  | --- | --- | 0.00038 | 27 | 0.0027 | 30 | --- | --- | 0.0013 | 35 | 0.00094 | 43 |
| 355 | GO:0030422 | P | production of siRNA involved in RNA interference |  |  |  |  |  |  | --- | --- | 0.00038 | 10 | 0.00086 | 11 | 0.0017 | 12 | 4.2e-06 | 16 | 7.1e-06 | 18 |
| 356 | GO:0000911 | P | cytokinesis by cell plate formation |  |  |  |  |  |  | --- | --- | 0.00038 | 10 | --- | --- | --- | --- | --- | --- | 0.0024 | 13 |
| 357 | GO:0051052 | P | regulation of DNA metabolic process |  |  |  |  |  |  | --- | --- | 0.00047 | 12 | --- | --- | --- | --- | 0.016 | 12 | 0.0013 | 17 |
| 358 | GO:0033044 | P | regulation of chromosome organization |  |  |  |  |  |  | --- | --- | 0.0005 | 7 | 0.0005 | 8 | 0.0005 | 9 | 0.00028 | 9 | --- | --- |
| 359 | GO:0051321 | P | meiotic cell cycle |  |  |  |  |  |  | --- | --- | 0.00058 | 16 | --- | --- | --- | --- | --- | --- | 0.0061 | 22 |
| 360 | GO:0051128 | P | regulation of cellular component organization |  |  |  |  |  |  | --- | --- | 0.00073 | 13 | 0.00035 | 16 | 0.00049 | 18 | 0.0017 | 16 | 4.8e-07 | 27 |
| 361 | GO:0006835 | P | dicarboxylic acid transport |  |  |  |  |  |  | --- | --- | 0.00076 | 6 | --- | --- | --- | --- | --- | --- | --- | --- |
| 362 | GO:0051028 | P | mRNA transport |  |  |  |  |  |  | --- | --- | 0.00076 | 6 | 0.015 | 5 | 0.041 | 5 | 0.0062 | 6 | --- | --- |
| 363 | GO:0009855 | P | determination of bilateral symmetry |  |  |  |  |  |  | --- | --- | 0.00076 | 6 | --- | --- | --- | --- | --- | --- | --- | --- |
| 364 | GO:0006406 | P | mRNA export from nucleus |  |  |  |  |  |  | --- | --- | 0.00076 | 6 | --- | --- | --- | --- | 0.029 | 5 | 0.02 | 6 |
| 365 | GO:0015743 | P | malate transport |  |  |  |  |  |  | --- | --- | 0.00076 | 6 | --- | --- | --- | --- | --- | --- | --- | --- |
| 366 | GO:0015740 | P | C4-dicarboxylate transport |  |  |  |  |  |  | --- | --- | 0.00076 | 6 | --- | --- | --- | --- | --- | --- | --- | --- |
| 367 | GO:0010073 | P | meristem maintenance |  |  |  |  |  |  | --- | --- | 0.00076 | 16 | 0.00083 | 19 | 0.02 | 18 | 0.002 | 20 | 0.0019 | 24 |
| 368 | GO:0016246 | P | RNA interference |  |  |  |  |  |  | --- | --- | 0.00077 | 11 | 0.00021 | 14 | 0.00058 | 15 | 6.8e-06 | 18 | 6e-06 | 21 |
| 369 | GO:0033205 | P | cytokinesis during cell cycle |  |  |  |  |  |  | --- | --- | 0.0008 | 10 | --- | --- | --- | --- | --- | --- | 0.002 | 14 |
| 370 | GO:0006275 | P | regulation of DNA replication |  |  |  |  |  |  | --- | --- | 0.00082 | 8 | 0.016 | 7 | --- | --- | 0.00089 | 10 | 3.4e-05 | 14 |
| 371 | GO:0010498 | P | proteasomal protein catabolic process |  |  |  |  |  |  | --- | --- | 0.00082 | 8 | 2.5e-06 | 13 | 5.2e-06 | 14 | 0.011 | 8 | 3.4e-05 | 14 |
| 372 | GO:0043331 | P | response to dsRNA |  |  |  |  |  |  | --- | --- | 0.001 | 10 | 0.0023 | 11 | 0.0046 | 12 | 1.8e-05 | 16 | 3.2e-05 | 18 |
| 373 | GO:0031050 | P | dsRNA fragmentation |  |  |  |  |  |  | --- | --- | 0.001 | 10 | 0.0023 | 11 | 0.0046 | 12 | 1.8e-05 | 16 | 3.2e-05 | 18 |
| 374 | GO:0070918 | P | production of small RNA involved in gene silencing by RNA |  |  |  |  |  |  | --- | --- | 0.001 | 10 | 0.0023 | 11 | 0.0046 | 12 | 1.8e-05 | 16 | 3.2e-05 | 18 |
| 375 | GO:0010388 | P | cullin deneddylation |  |  |  |  |  |  | --- | --- | 0.0011 | 7 | 0.0051 | 7 | 0.0003 | 10 | 0.00074 | 9 | --- | --- |
| 376 | GO:0010267 | P | production of ta-siRNAs involved in RNA interference |  |  |  |  |  |  | --- | --- | 0.0011 | 8 | 0.0062 | 8 | 0.0073 | 9 | 1.7e-05 | 13 | 1.4e-05 | 15 |
| 377 | GO:0001510 | P | RNA methylation |  |  |  |  |  |  | --- | --- | 0.0011 | 8 | 0.0062 | 8 | 0.0023 | 10 | 0.0044 | 9 | --- | --- |
| 378 | GO:0033043 | P | regulation of organelle organization |  |  |  |  |  |  | --- | --- | 0.0011 | 11 | 0.0011 | 13 | 0.00094 | 15 | 0.0014 | 14 | 1.1e-05 | 21 |
| 379 | GO:0007127 | P | meiosis I |  |  |  |  |  |  | --- | --- | 0.0012 | 10 | --- | --- | --- | --- | --- | --- | 0.0088 | 13 |
| 380 | GO:0006461 | P | protein complex assembly |  |  |  |  |  |  | --- | --- | 0.0012 | 23 | 0.00028 | 30 | 0.00049 | 34 | 1.3e-06 | 40 | 1e-05 | 45 |
| 381 | GO:0048523 | P | negative regulation of cellular process |  |  |  |  |  |  | --- | --- | 0.0012 | 33 | 0.0033 | 39 | --- | --- | 0.0095 | 42 | 0.0038 | 54 |
| 382 | GO:0070271 | P | protein complex biogenesis |  |  |  |  |  |  | --- | --- | 0.0012 | 23 | 0.00028 | 30 | 0.00049 | 34 | 1.3e-06 | 40 | 1e-05 | 45 |
| 383 | GO:0051168 | P | nuclear export |  |  |  |  |  |  | --- | --- | 0.0015 | 7 | --- | --- | --- | --- | --- | --- | --- | --- |
| 384 | GO:0007129 | P | synapsis |  |  |  |  |  |  | --- | --- | 0.0015 | 7 | --- | --- | --- | --- | --- | --- | --- | --- |
| 385 | GO:0070192 | P | chromosome organization involved in meiosis |  |  |  |  |  |  | --- | --- | 0.0015 | 7 | --- | --- | --- | --- | --- | --- | --- | --- |
| 386 | GO:0043161 | P | proteasomal ubiquitin-dependent protein catabolic process |  |  |  |  |  |  | --- | --- | 0.0015 | 7 | 0.00039 | 9 | 0.002 | 9 | --- | --- | 0.018 | 8 |
| 387 | GO:0035266 | P | meristem growth |  |  |  |  |  |  | --- | --- | 0.0015 | 10 | 0.0036 | 11 | 0.007 | 12 | 0.0013 | 13 | 0.00019 | 17 |
| 388 | GO:0043648 | P | dicarboxylic acid metabolic process |  |  |  |  |  |  | --- | --- | 0.0015 | 17 | 0.011 | 18 | --- | --- | 0.0059 | 21 | --- | --- |
| 389 | GO:0006733 | P | oxidoreduction coenzyme metabolic process |  |  |  |  |  |  | --- | --- | 0.0016 | 14 | 5.3e-05 | 20 | 4.4e-05 | 23 | 2e-06 | 25 | 2.7e-06 | 29 |
| 390 | GO:0000910 | P | cytokinesis |  |  |  |  |  |  | --- | --- | 0.0016 | 12 | --- | --- | --- | --- | --- | --- | 0.029 | 15 |
| 391 | GO:0048519 | P | negative regulation of biological process |  |  |  |  |  |  | --- | --- | 0.0018 | 51 | 0.004 | 62 | --- | --- | 0.049 | 64 | 0.013 | 85 |
| 392 | GO:0009086 | P | methionine biosynthetic process |  |  |  |  |  |  | --- | --- | 0.0019 | 8 | 0.01 | 8 | --- | --- | 0.00017 | 12 | 0.00044 | 13 |
| 393 | GO:0000338 | P | protein deneddylation |  |  |  |  |  |  | --- | --- | 0.0021 | 7 | 0.0095 | 7 | 0.00072 | 10 | 0.0016 | 9 | 4.8e-05 | 13 |
| 394 | GO:0043603 | P | cellular amide metabolic process |  |  |  |  |  |  | --- | --- | 0.0022 | 12 | 0.00011 | 17 | 0.00049 | 18 | 2.3e-05 | 20 | 1.2e-05 | 24 |
| 395 | GO:0034504 | P | protein localization in nucleus |  |  |  |  |  |  | --- | --- | 0.0022 | 12 | 0.046 | 11 | --- | --- | --- | --- | --- | --- |
| 396 | GO:0048638 | P | regulation of developmental growth |  |  |  |  |  |  | --- | --- | 0.0023 | 11 | 0.0023 | 13 | 0.015 | 13 | 0.0032 | 14 | 3.8e-05 | 21 |
| 397 | GO:0007131 | P | reciprocal meiotic recombination |  |  |  |  |  |  | --- | --- | 0.0025 | 8 | --- | --- | --- | --- | --- | --- | 0.0062 | 11 |
| 398 | GO:0001522 | P | pseudouridine synthesis |  |  |  |  |  |  | --- | --- | 0.0025 | 8 | --- | --- | --- | --- | --- | --- | 0.046 | 9 |
| 399 | GO:0050658 | P | RNA transport |  |  |  |  |  |  | --- | --- | 0.0028 | 6 | 0.044 | 5 | --- | --- | 0.022 | 6 | 0.02 | 7 |
| 400 | GO:0050657 | P | nucleic acid transport |  |  |  |  |  |  | --- | --- | 0.0028 | 6 | 0.044 | 5 | --- | --- | 0.022 | 6 | 0.02 | 7 |
| 401 | GO:0051817 | P | modification of morphology or physiology of other organism during symbiotic interaction |  |  |  |  |  |  | --- | --- | 0.0028 | 6 | 0.01 | 6 | 0.033 | 6 | --- | --- | --- | --- |
| 402 | GO:0044003 | P | modification by symbiont of host morphology or physiology |  |  |  |  |  |  | --- | --- | 0.0028 | 6 | 0.01 | 6 | 0.033 | 6 | --- | --- | --- | --- |
| 403 | GO:0051236 | P | establishment of RNA localization |  |  |  |  |  |  | --- | --- | 0.0028 | 6 | 0.044 | 5 | --- | --- | 0.022 | 6 | 0.02 | 7 |
| 404 | GO:0006739 | P | NADP metabolic process |  |  |  |  |  |  | --- | --- | 0.0028 | 10 | 1.4e-05 | 16 | 1.6e-05 | 18 | 6.8e-06 | 18 | 1.8e-06 | 22 |
| 405 | GO:0006405 | P | RNA export from nucleus |  |  |  |  |  |  | --- | --- | 0.0028 | 6 | --- | --- | --- | --- | --- | --- | --- | --- |
| 406 | GO:0006403 | P | RNA localization |  |  |  |  |  |  | --- | --- | 0.0028 | 6 | 0.044 | 5 | --- | --- | 0.022 | 6 | 0.02 | 7 |
| 407 | GO:0010264 | P | myo-inositol hexakisphosphate biosynthetic process |  |  |  |  |  |  | --- | --- | 0.0028 | 5 | --- | --- | --- | --- | --- | --- | --- | --- |
| 408 | GO:0016556 | P | mRNA modification |  |  |  |  |  |  | --- | --- | 0.0028 | 5 | --- | --- | --- | --- | 0.018 | 5 | --- | --- |
| 409 | GO:0010215 | P | cellulose microfibril organization |  |  |  |  |  |  | --- | --- | 0.0028 | 5 | 0.0093 | 5 | --- | --- | 0.018 | 5 | 0.046 | 5 |
| 410 | GO:0016572 | P | histone phosphorylation |  |  |  |  |  |  | --- | --- | 0.0028 | 5 | --- | --- | --- | --- | --- | --- | --- | --- |
| 411 | GO:0032958 | P | inositol phosphate biosynthetic process |  |  |  |  |  |  | --- | --- | 0.0028 | 5 | --- | --- | --- | --- | --- | --- | --- | --- |
| 412 | GO:0033517 | P | myo-inositol hexakisphosphate metabolic process |  |  |  |  |  |  | --- | --- | 0.0028 | 5 | --- | --- | --- | --- | --- | --- | --- | --- |
| 413 | GO:0051169 | P | nuclear transport |  |  |  |  |  |  | --- | --- | 0.0029 | 15 | 0.0031 | 18 | --- | --- | 0.0013 | 21 | 0.003 | 24 |
| 414 | GO:0006913 | P | nucleocytoplasmic transport |  |  |  |  |  |  | --- | --- | 0.0029 | 15 | 0.0031 | 18 | --- | --- | 0.0013 | 21 | 0.003 | 24 |
| 415 | GO:0008299 | P | isoprenoid biosynthetic process |  |  |  |  |  |  | --- | --- | 0.0029 | 22 | 0.019 | 24 | 0.014 | 29 | 0.0033 | 30 | 0.00038 | 40 |
| 416 | GO:0010564 | P | regulation of cell cycle process |  |  |  |  |  |  | --- | --- | 0.003 | 9 | --- | --- | --- | --- | 0.047 | 9 | 0.014 | 12 |
| 417 | GO:0016192 | P | vesicle-mediated transport |  |  |  |  |  |  | --- | --- | 0.0032 | 35 | 0.0024 | 44 | 0.00049 | 55 | 0.0013 | 51 | 1.4e-07 | 79 |
| 418 | GO:0010629 | P | negative regulation of gene expression |  |  |  |  |  |  | --- | --- | 0.0032 | 28 | 0.014 | 32 | --- | --- | 0.0096 | 37 | 0.02 | 44 |
| 419 | GO:0006555 | P | methionine metabolic process |  |  |  |  |  |  | --- | --- | 0.0035 | 13 | 0.032 | 13 | --- | --- | 0.0043 | 17 | 0.0011 | 22 |
| 420 | GO:0051607 | P | defense response to virus |  |  |  |  |  |  | --- | --- | 0.0037 | 9 | 0.021 | 9 | --- | --- | 6.8e-05 | 15 | 0.0001 | 17 |
| 421 | GO:0006606 | P | protein import into nucleus |  |  |  |  |  |  | --- | --- | 0.0037 | 11 | 0.027 | 11 | --- | --- | --- | --- | --- | --- |
| 422 | GO:0045132 | P | meiotic chromosome segregation |  |  |  |  |  |  | --- | --- | 0.0038 | 6 | --- | --- | 0.013 | 7 | --- | --- | --- | --- |
| 423 | GO:0009799 | P | specification of symmetry |  |  |  |  |  |  | --- | --- | 0.0038 | 6 | --- | --- | --- | --- | --- | --- | --- | --- |
| 424 | GO:0051170 | P | nuclear import |  |  |  |  |  |  | --- | --- | 0.0043 | 11 | 0.031 | 11 | --- | --- | --- | --- | --- | --- |
| 425 | GO:0010075 | P | regulation of meristem growth |  |  |  |  |  |  | --- | --- | 0.0044 | 9 | 0.0088 | 10 | 0.0056 | 12 | 0.0031 | 12 | 0.00041 | 16 |
| 426 | GO:0032879 | P | regulation of localization |  |  |  |  |  |  | --- | --- | 0.0044 | 9 | 0.00088 | 12 | 0.016 | 11 | 0.026 | 10 | 0.0088 | 13 |
| 427 | GO:0009119 | P | ribonucleoside metabolic process |  |  |  |  |  |  | --- | --- | 0.0045 | 10 | --- | --- | --- | --- | 0.034 | 11 | 0.016 | 14 |
| 428 | GO:0043069 | P | negative regulation of programmed cell death |  |  |  |  |  |  | --- | --- | 0.0048 | 7 | 0.021 | 7 | --- | --- | --- | --- | 0.021 | 9 |
| 429 | GO:0007346 | P | regulation of mitotic cell cycle |  |  |  |  |  |  | --- | --- | 0.0048 | 7 | --- | --- | --- | --- | --- | --- | 0.021 | 9 |
| 430 | GO:0043085 | P | positive regulation of catalytic activity |  |  |  |  |  |  | --- | --- | 0.0049 | 11 | --- | --- | --- | --- | --- | --- | 0.029 | 15 |
| 431 | GO:0015992 | P | proton transport |  |  |  |  |  |  | --- | --- | 0.005 | 13 | 0.00054 | 18 | 0.028 | 16 | 0.0068 | 17 | 0.0019 | 22 |
| 432 | GO:0006818 | P | hydrogen transport |  |  |  |  |  |  | --- | --- | 0.005 | 13 | 0.00054 | 18 | 0.028 | 16 | 0.0068 | 17 | 0.0019 | 22 |
| 433 | GO:0051604 | P | protein maturation |  |  |  |  |  |  | --- | --- | 0.0053 | 9 | --- | --- | --- | --- | --- | --- | --- | --- |
| 434 | GO:0006342 | P | chromatin silencing |  |  |  |  |  |  | --- | --- | 0.0053 | 9 | 0.00034 | 13 | 0.00082 | 14 | 0.00012 | 15 | 6.4e-06 | 20 |
| 435 | GO:0016481 | P | negative regulation of transcription |  |  |  |  |  |  | --- | --- | 0.0056 | 16 | 0.0036 | 20 | 0.019 | 21 | 0.0021 | 23 | 0.0016 | 28 |
| 436 | GO:0007017 | P | microtubule-based process |  |  |  |  |  |  | --- | --- | 0.0056 | 19 | 0.046 | 20 | 0.00081 | 30 | 0.00032 | 30 | 1.7e-05 | 40 |
| 437 | GO:0044093 | P | positive regulation of molecular function |  |  |  |  |  |  | --- | --- | 0.0057 | 11 | --- | --- | --- | --- | --- | --- | 0.034 | 15 |
| 438 | GO:0043623 | P | cellular protein complex assembly |  |  |  |  |  |  | --- | --- | 0.0057 | 20 | --- | --- | --- | --- | 0.0079 | 27 | 0.022 | 31 |
| 439 | GO:0045934 | P | negative regulation of nucleobase, nucleoside, nucleotide and nucleic acid metabolic process |  |  |  |  |  |  | --- | --- | 0.006 | 17 | 0.01 | 20 | 0.027 | 22 | 0.0068 | 23 | 0.0063 | 28 |
| 440 | GO:0051172 | P | negative regulation of nitrogen compound metabolic process |  |  |  |  |  |  | --- | --- | 0.006 | 17 | 0.01 | 20 | 0.027 | 22 | 0.0068 | 23 | 0.0063 | 28 |
| 441 | GO:0010605 | P | negative regulation of macromolecule metabolic process |  |  |  |  |  |  | --- | --- | 0.0065 | 29 | 0.049 | 32 | --- | --- | 0.041 | 37 | --- | --- |
| 442 | GO:0009062 | P | fatty acid catabolic process |  |  |  |  |  |  | --- | --- | 0.0066 | 11 | 0.0076 | 13 | --- | --- | 0.027 | 13 | 0.00063 | 20 |
| 443 | GO:0042991 | P | transcription factor import into nucleus |  |  |  |  |  |  | --- | --- | 0.0069 | 5 | --- | --- | --- | --- | --- | --- | --- | --- |
| 444 | GO:0007030 | P | Golgi organization |  |  |  |  |  |  | --- | --- | 0.0069 | 5 | --- | --- | --- | --- | --- | --- | 0.03 | 6 |
| 445 | GO:0007051 | P | spindle organization |  |  |  |  |  |  | --- | --- | 0.0069 | 5 | --- | --- | --- | --- | 0.042 | 5 | --- | --- |
| 446 | GO:0007276 | P | gamete generation |  |  |  |  |  |  | --- | --- | 0.0073 | 8 | --- | --- | --- | --- | --- | --- | --- | --- |
| 447 | GO:0070647 | P | protein modification by small protein conjugation or removal |  |  |  |  |  |  | --- | --- | 0.0077 | 23 | 0.017 | 27 | 0.0032 | 35 | 0.00064 | 36 | 0.00015 | 46 |
| 448 | GO:0016458 | P | gene silencing |  |  |  |  |  |  | --- | --- | 0.0079 | 21 | 0.013 | 25 | --- | --- | 0.0081 | 29 | 0.0062 | 36 |
| 449 | GO:0006635 | P | fatty acid beta-oxidation |  |  |  |  |  |  | --- | --- | 0.0084 | 10 | 0.0078 | 12 | --- | --- | --- | --- | 0.0026 | 17 |
| 450 | GO:0030258 | P | lipid modification |  |  |  |  |  |  | --- | --- | 0.0085 | 12 | 2.8e-05 | 20 | 0.0032 | 18 | 0.0015 | 18 | 8.8e-06 | 27 |
| 451 | GO:0019637 | P | organophosphate metabolic process |  |  |  |  |  |  | --- | --- | 0.0093 | 20 | 5.2e-06 | 34 | 6.3e-05 | 36 | 3.9e-06 | 38 | 2.9e-09 | 54 |
| 452 | GO:0000725 | P | recombinational repair |  |  |  |  |  |  | --- | --- | 0.0097 | 6 | 0.0024 | 8 | --- | --- | 0.0004 | 10 | 0.00019 | 12 |
| 453 | GO:0043647 | P | inositol phosphate metabolic process |  |  |  |  |  |  | --- | --- | 0.0097 | 6 | --- | --- | --- | --- | 0.0061 | 8 | 0.0082 | 9 |
| 454 | GO:0000724 | P | double-strand break repair via homologous recombination |  |  |  |  |  |  | --- | --- | 0.0097 | 6 | 0.0024 | 8 | --- | --- | 0.0004 | 10 | 0.00019 | 12 |
| 455 | GO:0007015 | P | actin filament organization |  |  |  |  |  |  | --- | --- | 0.0098 | 10 | 0.0091 | 12 | 0.0086 | 14 | 0.0047 | 14 | 0.0005 | 19 |
| 456 | GO:0060548 | P | negative regulation of cell death |  |  |  |  |  |  | --- | --- | 0.0099 | 7 | 0.042 | 7 | --- | --- | --- | --- | 0.017 | 10 |
| 457 | GO:0015931 | P | nucleobase, nucleoside, nucleotide and nucleic acid transport |  |  |  |  |  |  | --- | --- | 0.0099 | 11 | --- | --- | --- | --- | --- | --- | --- | --- |
| 458 | GO:0052018 | P | modulation by symbiont of RNA levels in host |  |  |  |  |  |  | --- | --- | 0.01 | 5 | 0.0072 | 6 | 0.024 | 6 | --- | --- | --- | --- |
| 459 | GO:0052249 | P | modulation of RNA levels in other organism during symbiotic interaction |  |  |  |  |  |  | --- | --- | 0.01 | 5 | 0.0072 | 6 | 0.024 | 6 | --- | --- | --- | --- |
| 460 | GO:0009616 | P | virus induced gene silencing |  |  |  |  |  |  | --- | --- | 0.01 | 5 | 0.0072 | 6 | 0.024 | 6 | --- | --- | --- | --- |
| 461 | GO:0009867 | P | jasmonic acid mediated signaling pathway |  |  |  |  |  |  | --- | --- | 0.011 | 11 | 0.014 | 13 | --- | --- | --- | --- | 0.008 | 18 |
| 462 | GO:0046496 | P | nicotinamide nucleotide metabolic process |  |  |  |  |  |  | --- | --- | 0.011 | 10 | 0.00015 | 16 | 0.00019 | 18 | 8.8e-05 | 18 | 3.2e-05 | 22 |
| 463 | GO:0006769 | P | nicotinamide metabolic process |  |  |  |  |  |  | --- | --- | 0.011 | 10 | 0.00015 | 16 | 0.00019 | 18 | 8.8e-05 | 18 | 3.2e-05 | 22 |
| 464 | GO:0042545 | P | cell wall modification |  |  |  |  |  |  | --- | --- | 0.012 | 19 | --- | --- | --- | --- | 0.00096 | 30 | --- | --- |
| 465 | GO:0051701 | P | interaction with host |  |  |  |  |  |  | --- | --- | 0.013 | 6 | 0.045 | 6 | --- | --- | 0.00061 | 10 | 8e-05 | 13 |
| 466 | GO:0009116 | P | nucleoside metabolic process |  |  |  |  |  |  | --- | --- | 0.013 | 11 | --- | --- | --- | --- | --- | --- | 0.02 | 17 |
| 467 | GO:0055085 | P | transmembrane transport |  |  |  |  |  |  | --- | --- | 0.014 | 30 | --- | --- | --- | --- | 0.0025 | 46 | 0.013 | 53 |
| 468 | GO:0009072 | P | aromatic amino acid family metabolic process |  |  |  |  |  |  | --- | --- | 0.014 | 13 | 0.00032 | 20 | --- | --- | 0.00034 | 22 | 0.0019 | 24 |
| 469 | GO:0016558 | P | protein import into peroxisome matrix |  |  |  |  |  |  | --- | --- | 0.014 | 5 | 0.0005 | 8 | 0.0087 | 7 | 0.022 | 6 | --- | --- |
| 470 | GO:0006354 | P | RNA elongation |  |  |  |  |  |  | --- | --- | 0.014 | 5 | 0.044 | 5 | 0.0087 | 7 | 0.022 | 6 | 0.02 | 7 |
| 471 | GO:0006021 | P | inositol biosynthetic process |  |  |  |  |  |  | --- | --- | 0.014 | 5 | --- | --- | --- | --- | 0.022 | 6 | 0.02 | 7 |
| 472 | GO:0046483 | P | heterocycle metabolic process |  |  |  |  |  |  | --- | --- | 0.015 | 48 | 0.013 | 61 | --- | --- | 0.00078 | 77 | 3.4e-05 | 103 |
| 473 | GO:0006260 | P | DNA replication |  |  |  |  |  |  | --- | --- | 0.016 | 18 | 1.3e-05 | 31 | 0.00025 | 32 | 0.00022 | 31 | 6.3e-06 | 42 |
| 474 | GO:0008654 | P | phospholipid biosynthetic process |  |  |  |  |  |  | --- | --- | 0.016 | 15 | 0.00086 | 22 | 0.0029 | 24 | 0.0013 | 24 | 9.1e-06 | 35 |
| 475 | GO:0009892 | P | negative regulation of metabolic process |  |  |  |  |  |  | --- | --- | 0.016 | 29 | --- | --- | --- | --- | --- | --- | --- | --- |
| 476 | GO:0034470 | P | ncRNA processing |  |  |  |  |  |  | --- | --- | 0.017 | 23 | 0.038 | 27 | --- | --- | 0.00028 | 39 | --- | --- |
| 477 | GO:0010558 | P | negative regulation of macromolecule biosynthetic process |  |  |  |  |  |  | --- | --- | 0.017 | 16 | 0.012 | 20 | --- | --- | 0.0084 | 23 | 0.0081 | 28 |
| 478 | GO:0045814 | P | negative regulation of gene expression, epigenetic |  |  |  |  |  |  | --- | --- | 0.017 | 9 | 0.0016 | 13 | 0.0042 | 14 | 0.00078 | 15 | 6.2e-05 | 20 |
| 479 | GO:0009640 | P | photomorphogenesis |  |  |  |  |  |  | --- | --- | 0.019 | 13 | 0.0012 | 19 | --- | --- | 0.0031 | 20 | 0.0062 | 23 |
| 480 | GO:0019395 | P | fatty acid oxidation |  |  |  |  |  |  | --- | --- | 0.019 | 10 | 0.019 | 12 | --- | --- | --- | --- | 0.0087 | 17 |
| 481 | GO:0034440 | P | lipid oxidation |  |  |  |  |  |  | --- | --- | 0.019 | 10 | 0.019 | 12 | --- | --- | --- | --- | 0.0087 | 17 |
| 482 | GO:0009067 | P | aspartate family amino acid biosynthetic process |  |  |  |  |  |  | --- | --- | 0.019 | 10 | --- | --- | --- | --- | 0.0044 | 15 | 0.019 | 16 |
| 483 | GO:0009066 | P | aspartate family amino acid metabolic process |  |  |  |  |  |  | --- | --- | 0.019 | 15 | --- | --- | --- | --- | 0.0071 | 22 | 0.02 | 25 |
| 484 | GO:0010207 | P | photosystem II assembly |  |  |  |  |  |  | --- | --- | 0.019 | 5 | 0.015 | 6 | 0.013 | 7 | 9.5e-05 | 10 | 0.0024 | 9 |
| 485 | GO:0046173 | P | polyol biosynthetic process |  |  |  |  |  |  | --- | --- | 0.019 | 5 | --- | --- | --- | --- | 0.031 | 6 | 0.029 | 7 |
| 486 | GO:0031047 | P | gene silencing by RNA |  |  |  |  |  |  | --- | --- | 0.02 | 17 | 0.0045 | 23 | 0.028 | 24 | 0.0038 | 26 | 0.00074 | 34 |
| 487 | GO:0015979 | P | photosynthesis |  |  |  |  |  |  | --- | --- | 0.02 | 22 | 0.013 | 28 | --- | --- | --- | --- | --- | --- |
| 488 | GO:0015849 | P | organic acid transport |  |  |  |  |  |  | --- | --- | 0.02 | 14 | --- | --- | --- | --- | --- | --- | --- | --- |
| 489 | GO:0046942 | P | carboxylic acid transport |  |  |  |  |  |  | --- | --- | 0.02 | 14 | --- | --- | --- | --- | --- | --- | --- | --- |
| 490 | GO:0019362 | P | pyridine nucleotide metabolic process |  |  |  |  |  |  | --- | --- | 0.022 | 10 | 0.00043 | 16 | 0.00059 | 18 | 0.00028 | 18 | 0.00012 | 22 |
| 491 | GO:0010027 | P | thylakoid membrane organization |  |  |  |  |  |  | --- | --- | 0.022 | 7 | 0.00086 | 11 | 0.005 | 11 | 6.8e-05 | 14 | 0.00025 | 15 |
| 492 | GO:0009668 | P | plastid membrane organization |  |  |  |  |  |  | --- | --- | 0.022 | 7 | 0.00086 | 11 | 0.005 | 11 | 6.8e-05 | 14 | 0.00025 | 15 |
| 493 | GO:0009100 | P | glycoprotein metabolic process |  |  |  |  |  |  | --- | --- | 0.023 | 13 | --- | --- | --- | --- | 0.04 | 17 | 0.004 | 24 |
| 494 | GO:0009890 | P | negative regulation of biosynthetic process |  |  |  |  |  |  | --- | --- | 0.023 | 16 | 0.017 | 20 | 0.046 | 22 | 0.0064 | 24 | 0.007 | 29 |
| 495 | GO:0031327 | P | negative regulation of cellular biosynthetic process |  |  |  |  |  |  | --- | --- | 0.023 | 16 | 0.017 | 20 | 0.046 | 22 | 0.0064 | 24 | 0.007 | 29 |
| 496 | GO:0046486 | P | glycerolipid metabolic process |  |  |  |  |  |  | --- | --- | 0.023 | 12 | 6.1e-06 | 23 | 2e-05 | 25 | 7.6e-06 | 25 | 1e-09 | 38 |
| 497 | GO:0006725 | P | cellular aromatic compound metabolic process |  |  |  |  |  |  | --- | --- | 0.024 | 42 | 0.0018 | 59 | --- | --- | 0.0068 | 64 | 0.0015 | 84 |
| 498 | GO:0018130 | P | heterocycle biosynthetic process |  |  |  |  |  |  | --- | --- | 0.024 | 18 | --- | --- | --- | --- | 0.0019 | 29 | 0.02 | 31 |
| 499 | GO:0042440 | P | pigment metabolic process |  |  |  |  |  |  | --- | --- | 0.025 | 19 | --- | --- | --- | --- | --- | --- | 0.0022 | 37 |
| 500 | GO:0045489 | P | pectin biosynthetic process |  |  |  |  |  |  | --- | --- | 0.025 | 6 | --- | --- | --- | --- | --- | --- | --- | --- |
| 501 | GO:0006418 | P | tRNA aminoacylation for protein translation |  |  |  |  |  |  | --- | --- | 0.026 | 14 | 0.027 | 17 | 0.028 | 20 | --- | --- | --- | --- |
| 502 | GO:0043038 | P | amino acid activation |  |  |  |  |  |  | --- | --- | 0.026 | 14 | 0.027 | 17 | 0.028 | 20 | --- | --- | --- | --- |
| 503 | GO:0043039 | P | tRNA aminoacylation |  |  |  |  |  |  | --- | --- | 0.026 | 14 | 0.027 | 17 | 0.028 | 20 | --- | --- | --- | --- |
| 504 | GO:0006720 | P | isoprenoid metabolic process |  |  |  |  |  |  | --- | --- | 0.028 | 22 | --- | --- | --- | --- | 0.029 | 31 | 0.0078 | 41 |
| 505 | GO:0046417 | P | chorismate metabolic process |  |  |  |  |  |  | --- | --- | 0.028 | 11 | 0.016 | 14 | --- | --- | --- | --- | --- | --- |
| 506 | GO:0009073 | P | aromatic amino acid family biosynthetic process |  |  |  |  |  |  | --- | --- | 0.028 | 11 | 0.016 | 14 | --- | --- | --- | --- | --- | --- |
| 507 | GO:0016042 | P | lipid catabolic process |  |  |  |  |  |  | --- | --- | 0.028 | 14 | 0.03 | 17 | --- | --- | 0.018 | 20 | 0.0028 | 27 |
| 508 | GO:0007005 | P | mitochondrion organization |  |  |  |  |  |  | --- | --- | 0.028 | 9 | --- | --- | 0.0086 | 14 | 0.0017 | 15 | 0.0032 | 17 |
| 509 | GO:0048509 | P | regulation of meristem development |  |  |  |  |  |  | --- | --- | 0.03 | 13 | --- | --- | --- | --- | --- | --- | 0.044 | 21 |
| 510 | GO:0019438 | P | aromatic compound biosynthetic process |  |  |  |  |  |  | --- | --- | 0.03 | 28 | 0.0068 | 38 | --- | --- | --- | --- | 0.04 | 49 |
| 511 | GO:0006952 | P | defense response |  |  |  |  |  |  | --- | --- | 0.03 | 70 | --- | --- | --- | --- | --- | --- | --- | --- |
| 512 | GO:0006020 | P | inositol metabolic process |  |  |  |  |  |  | --- | --- | 0.031 | 6 | --- | --- | --- | --- | 0.026 | 8 | 0.013 | 10 |
| 513 | GO:0031163 | P | metallo-sulfur cluster assembly |  |  |  |  |  |  | --- | --- | 0.031 | 7 | 0.0014 | 11 | --- | --- | 0.0015 | 12 | 0.011 | 12 |
| 514 | GO:0016226 | P | iron-sulfur cluster assembly |  |  |  |  |  |  | --- | --- | 0.031 | 7 | 0.0014 | 11 | --- | --- | 0.0015 | 12 | 0.011 | 12 |
| 515 | GO:0008033 | P | tRNA processing |  |  |  |  |  |  | --- | --- | 0.031 | 11 | --- | --- | --- | --- | 0.031 | 15 | --- | --- |
| 516 | GO:0009832 | P | plant-type cell wall biogenesis |  |  |  |  |  |  | --- | --- | 0.031 | 11 | --- | --- | --- | --- | --- | --- | --- | --- |
| 517 | GO:0006625 | P | protein targeting to peroxisome |  |  |  |  |  |  | --- | --- | 0.032 | 5 | 8e-05 | 10 | 0.007 | 8 | 0.0043 | 8 | --- | --- |
| 518 | GO:0043574 | P | peroxisomal transport |  |  |  |  |  |  | --- | --- | 0.032 | 5 | 8e-05 | 10 | 0.007 | 8 | 0.0043 | 8 | --- | --- |
| 519 | GO:0015706 | P | nitrate transport |  |  |  |  |  |  | --- | --- | 0.032 | 5 | 0.027 | 6 | --- | --- | --- | --- | 0.0057 | 9 |
| 520 | GO:0009888 | P | tissue development |  |  |  |  |  |  | --- | --- | 0.033 | 38 | 0.0038 | 53 | --- | --- | 0.034 | 55 | 0.0057 | 74 |
| 521 | GO:0006487 | P | protein amino acid N-linked glycosylation |  |  |  |  |  |  | --- | --- | 0.037 | 6 | --- | --- | --- | --- | --- | --- | 0.017 | 10 |
| 522 | GO:0006261 | P | DNA-dependent DNA replication |  |  |  |  |  |  | --- | --- | 0.038 | 11 | 0.0016 | 17 | --- | --- | 0.00058 | 20 | 7.3e-05 | 26 |
| 523 | GO:0051301 | P | cell division |  |  |  |  |  |  | --- | --- | 0.039 | 15 | --- | --- | --- | --- | --- | --- | --- | --- |
| 524 | GO:0045017 | P | glycerolipid biosynthetic process |  |  |  |  |  |  | --- | --- | 0.041 | 9 | 0.0057 | 13 | 0.0059 | 15 | 0.0079 | 14 | 7.1e-06 | 24 |
| 525 | GO:0007031 | P | peroxisome organization |  |  |  |  |  |  | --- | --- | 0.043 | 8 | 0.00042 | 14 | --- | --- | 0.013 | 12 | 0.001 | 17 |
| 526 | GO:0046165 | P | alcohol biosynthetic process |  |  |  |  |  |  | --- | --- | 0.047 | 9 | --- | --- | --- | --- | 0.0094 | 14 | 0.003 | 18 |
| 527 | GO:0040029 | P | regulation of gene expression, epigenetic |  |  |  |  |  |  | --- | --- | 0.048 | 23 | --- | --- | --- | --- | 0.026 | 34 | --- | --- |
| 528 | GO:0016705 | F | oxidoreductase activity, acting on paired donors, with incorporation or reduction of molecular oxygen |  |  |  |  |  |  | --- | --- | 1e-07 | 40 | 1.3e-13 | 61 | 5.8e-15 | 72 | 9.5e-20 | 80 | 7e-17 | 88 |
| 529 | GO:0004190 | F | aspartic-type endopeptidase activity |  |  |  |  |  |  | --- | --- | 2.2e-06 | 24 | --- | --- | --- | --- | 6e-05 | 28 | 7.5e-06 | 36 |
| 530 | GO:0070001 | F | aspartic-type peptidase activity |  |  |  |  |  |  | --- | --- | 2.2e-06 | 24 | --- | --- | --- | --- | 6e-05 | 28 | 7.5e-06 | 36 |
| 531 | GO:0004497 | F | monooxygenase activity |  |  |  |  |  |  | --- | --- | 1.1e-05 | 27 | 3e-12 | 46 | 6.5e-11 | 49 | 3.6e-16 | 58 | 9.7e-14 | 63 |
| 532 | GO:0004386 | F | helicase activity |  |  |  |  |  |  | --- | --- | 0.00059 | 29 | 0.0014 | 34 | 5.1e-07 | 50 | 0.0017 | 38 | 4.8e-07 | 59 |
| 533 | GO:0050661 | F | NADP or NADPH binding |  |  |  |  |  |  | --- | --- | 0.0006 | 9 | 3.7e-07 | 15 | 3.3e-08 | 18 | 1.2e-05 | 14 | 2.4e-07 | 19 |
| 534 | GO:0016417 | F | S-acyltransferase activity |  |  |  |  |  |  | --- | --- | 0.00061 | 8 | --- | --- | --- | --- | 0.0094 | 8 | 0.0012 | 11 |
| 535 | GO:0016757 | F | transferase activity, transferring glycosyl groups |  |  |  |  |  |  | --- | --- | 0.00098 | 61 | --- | --- | --- | --- | 0.032 | 78 | 0.022 | 100 |
| 536 | GO:0008375 | F | acetylglucosaminyltransferase activity |  |  |  |  |  |  | --- | --- | 0.0011 | 10 | 0.00016 | 13 | 0.00031 | 14 | 5.6e-05 | 15 | 0.00033 | 16 |
| 537 | GO:0008234 | F | cysteine-type peptidase activity |  |  |  |  |  |  | --- | --- | 0.0021 | 27 | 2e-05 | 39 | 8.4e-09 | 54 | 1.5e-08 | 52 | 9.2e-06 | 54 |
| 538 | GO:0051536 | F | iron-sulfur cluster binding |  |  |  |  |  |  | --- | --- | 0.0022 | 11 | 0.0058 | 12 | --- | --- | 0.00027 | 16 | 0.0019 | 17 |
| 539 | GO:0051540 | F | metal cluster binding |  |  |  |  |  |  | --- | --- | 0.0022 | 11 | 0.0058 | 12 | --- | --- | 0.00027 | 16 | 0.0019 | 17 |
| 540 | GO:0008810 | F | cellulase activity |  |  |  |  |  |  | --- | --- | 0.0034 | 5 | 0.01 | 5 | --- | --- | --- | --- | --- | --- |
| 541 | GO:0016645 | F | oxidoreductase activity, acting on the CH-NH group of donors |  |  |  |  |  |  | --- | --- | 0.0036 | 9 | --- | --- | --- | --- | 0.0066 | 11 | 0.019 | 12 |
| 542 | GO:0008194 | F | UDP-glycosyltransferase activity |  |  |  |  |  |  | --- | --- | 0.0041 | 30 | 0.035 | 33 | 0.008 | 42 | 0.0035 | 42 | 0.038 | 47 |
| 543 | GO:0001871 | F | pattern binding |  |  |  |  |  |  | --- | --- | 0.01 | 7 | 0.038 | 7 | --- | --- | --- | --- | --- | --- |
| 544 | GO:0030247 | F | polysaccharide binding |  |  |  |  |  |  | --- | --- | 0.01 | 7 | 0.038 | 7 | --- | --- | --- | --- | --- | --- |
| 545 | GO:0016835 | F | carbon-oxygen lyase activity |  |  |  |  |  |  | --- | --- | 0.014 | 23 | 0.0041 | 30 | 0.00025 | 39 | 0.0019 | 35 | 0.011 | 40 |
| 546 | GO:0016838 | F | carbon-oxygen lyase activity, acting on phosphates |  |  |  |  |  |  | --- | --- | 0.016 | 6 | 5.4e-06 | 12 | 7.4e-06 | 13 | 1e-06 | 14 | 8.3e-05 | 13 |
| 547 | GO:0009982 | F | pseudouridine synthase activity |  |  |  |  |  |  | --- | --- | 0.019 | 7 | --- | --- | --- | --- | --- | --- | --- | --- |
| 548 | GO:0004523 | F | ribonuclease H activity |  |  |  |  |  |  | --- | --- | 0.021 | 6 | --- | --- | 0.018 | 8 | --- | --- | --- | --- |
| 549 | GO:0016875 | F | ligase activity, forming carbon-oxygen bonds |  |  |  |  |  |  | --- | --- | 0.023 | 14 | 0.021 | 17 | 0.018 | 20 | --- | --- | --- | --- |
| 550 | GO:0016876 | F | ligase activity, forming aminoacyl-tRNA and related compounds |  |  |  |  |  |  | --- | --- | 0.023 | 14 | 0.021 | 17 | 0.018 | 20 | --- | --- | --- | --- |
| 551 | GO:0004812 | F | aminoacyl-tRNA ligase activity |  |  |  |  |  |  | --- | --- | 0.023 | 14 | 0.021 | 17 | 0.018 | 20 | --- | --- | --- | --- |
| 552 | GO:0016853 | F | isomerase activity |  |  |  |  |  |  | --- | --- | 0.027 | 31 | 0.022 | 39 | --- | --- | --- | --- | 0.009 | 58 |
| 553 | GO:0048038 | F | quinone binding |  |  |  |  |  |  | --- | --- | 0.032 | 5 | --- | --- | --- | --- | --- | --- | --- | --- |
| 554 | GO:0031406 | F | carboxylic acid binding |  |  |  |  |  |  | --- | --- | 0.032 | 11 | --- | --- | --- | --- | 0.0048 | 17 | --- | --- |
| 555 | GO:0016646 | F | oxidoreductase activity, acting on the CH-NH group of donors, NAD or NADP as acceptor |  |  |  |  |  |  | --- | --- | 0.042 | 5 | --- | --- | --- | --- | --- | --- | --- | --- |
| 556 | GO:0005576 | C | extracellular region |  |  |  |  |  |  | --- | --- | 1.9e-08 | 52 | 1.1e-06 | 58 | 5.9e-05 | 61 | 5.1e-10 | 75 | 8.9e-09 | 87 |
| 557 | GO:0000790 | C | nuclear chromatin |  |  |  |  |  |  | --- | --- | 0.0094 | 6 | --- | --- | --- | --- | 0.018 | 7 | 0.048 | 7 |
| 558 | GO:0016585 | C | chromatin remodeling complex |  |  |  |  |  |  | --- | --- | 0.034 | 7 | --- | --- | --- | --- | --- | --- | --- | --- |
| 559 | GO:0000228 | C | nuclear chromosome |  |  |  |  |  |  | --- | --- | 0.048 | 9 | 0.013 | 12 | --- | --- | 0.017 | 13 | 0.026 | 15 |
| 560 | GO:0006650 | P | glycerophospholipid metabolic process |  |  |  |  |  |  | --- | --- | --- | --- | 2.8e-06 | 22 | 2.2e-05 | 23 | 8e-05 | 21 | 1.4e-08 | 33 |
| 561 | GO:0044260 | P | cellular macromolecule metabolic process |  |  |  |  |  |  | --- | --- | --- | --- | 3.4e-05 | 646 | 0.012 | 741 | 0.00085 | 732 | 0.0001 | 960 |
| 562 | GO:0046395 | P | carboxylic acid catabolic process |  |  |  |  |  |  | --- | --- | --- | --- | 0.00024 | 25 | --- | --- | 0.00096 | 26 | 0.00026 | 33 |
| 563 | GO:0016054 | P | organic acid catabolic process |  |  |  |  |  |  | --- | --- | --- | --- | 0.00024 | 25 | --- | --- | 0.00096 | 26 | 0.00026 | 33 |
| 564 | GO:0030384 | P | phosphoinositide metabolic process |  |  |  |  |  |  | --- | --- | --- | --- | 0.00029 | 16 | 0.003 | 16 | 0.0094 | 14 | 7.2e-05 | 22 |
| 565 | GO:0048469 | P | cell maturation |  |  |  |  |  |  | --- | --- | --- | --- | 0.00035 | 16 | 0.0035 | 16 | 0.011 | 14 | 0.00025 | 21 |
| 566 | GO:0048765 | P | root hair cell differentiation |  |  |  |  |  |  | --- | --- | --- | --- | 0.00035 | 16 | 0.0035 | 16 | 0.011 | 14 | 0.00025 | 21 |
| 567 | GO:0048764 | P | trichoblast maturation |  |  |  |  |  |  | --- | --- | --- | --- | 0.00035 | 16 | 0.0035 | 16 | 0.011 | 14 | 0.00025 | 21 |
| 568 | GO:0050794 | P | regulation of cellular process |  |  |  |  |  |  | --- | --- | --- | --- | 0.00072 | 352 | --- | --- | 0.041 | 384 | 0.0011 | 521 |
| 569 | GO:0046164 | P | alcohol catabolic process |  |  |  |  |  |  | --- | --- | --- | --- | 0.00097 | 22 | 4.8e-05 | 29 | 0.0014 | 24 | 5.6e-05 | 33 |
| 570 | GO:0006007 | P | glucose catabolic process |  |  |  |  |  |  | --- | --- | --- | --- | 0.0011 | 21 | 0.00011 | 27 | 0.0031 | 22 | 0.00022 | 30 |
| 571 | GO:0019320 | P | hexose catabolic process |  |  |  |  |  |  | --- | --- | --- | --- | 0.0012 | 21 | 0.00012 | 27 | 0.0035 | 22 | 0.00026 | 30 |
| 572 | GO:0046365 | P | monosaccharide catabolic process |  |  |  |  |  |  | --- | --- | --- | --- | 0.0012 | 21 | 0.00012 | 27 | 0.0035 | 22 | 0.00026 | 30 |
| 573 | GO:0007264 | P | small GTPase mediated signal transduction |  |  |  |  |  |  | --- | --- | --- | --- | 0.0012 | 15 | --- | --- | 0.0008 | 17 | 0.00031 | 21 |
| 574 | GO:0019439 | P | aromatic compound catabolic process |  |  |  |  |  |  | --- | --- | --- | --- | 0.0015 | 7 | --- | --- | 0.0035 | 7 | 0.013 | 7 |
| 575 | GO:0008037 | P | cell recognition |  |  |  |  |  |  | --- | --- | --- | --- | 0.0022 | 12 | 1.6e-05 | 18 | 0.00091 | 14 | 5.2e-07 | 23 |
| 576 | GO:0048544 | P | recognition of pollen |  |  |  |  |  |  | --- | --- | --- | --- | 0.0022 | 12 | 1.6e-05 | 18 | 0.00091 | 14 | 5.2e-07 | 23 |
| 577 | GO:0021700 | P | developmental maturation |  |  |  |  |  |  | --- | --- | --- | --- | 0.0023 | 16 | 0.019 | 16 | --- | --- | 0.0025 | 21 |
| 578 | GO:0031048 | P | chromatin silencing by small RNA |  |  |  |  |  |  | --- | --- | --- | --- | 0.0024 | 7 | 0.033 | 6 | 0.0013 | 8 | --- | --- |
| 579 | GO:0009820 | P | alkaloid metabolic process |  |  |  |  |  |  | --- | --- | --- | --- | 0.0027 | 16 | 0.0044 | 18 | 0.0021 | 18 | 0.0013 | 22 |
| 580 | GO:0031407 | P | oxylipin metabolic process |  |  |  |  |  |  | --- | --- | --- | --- | 0.0028 | 13 | 0.018 | 13 | 0.0039 | 14 | 0.0063 | 16 |
| 581 | GO:0006289 | P | nucleotide-excision repair |  |  |  |  |  |  | --- | --- | --- | --- | 0.0034 | 8 | 0.0041 | 9 | --- | --- | --- | --- |
| 582 | GO:0000394 | P | RNA splicing, via endonucleolytic cleavage and ligation |  |  |  |  |  |  | --- | --- | --- | --- | 0.0034 | 8 | --- | --- | 0.00014 | 11 | 0.012 | 9 |
| 583 | GO:0043543 | P | protein amino acid acylation |  |  |  |  |  |  | --- | --- | --- | --- | 0.0036 | 7 | --- | --- | 0.008 | 7 | 0.00064 | 10 |
| 584 | GO:0043170 | P | macromolecule metabolic process |  |  |  |  |  |  | --- | --- | --- | --- | 0.0043 | 677 | --- | --- | 0.041 | 771 | 0.013 | 1013 |
| 585 | GO:0033865 | P | nucleoside bisphosphate metabolic process |  |  |  |  |  |  | --- | --- | --- | --- | 0.0048 | 6 | 0.016 | 6 | --- | --- | --- | --- |
| 586 | GO:0051567 | P | histone H3-K9 methylation |  |  |  |  |  |  | --- | --- | --- | --- | 0.0048 | 6 | --- | --- | --- | --- | --- | --- |
| 587 | GO:0015936 | P | coenzyme A metabolic process |  |  |  |  |  |  | --- | --- | --- | --- | 0.0048 | 6 | 0.016 | 6 | --- | --- | --- | --- |
| 588 | GO:0046474 | P | glycerophospholipid biosynthetic process |  |  |  |  |  |  | --- | --- | --- | --- | 0.0057 | 12 | 0.013 | 13 | --- | --- | 0.00023 | 19 |
| 589 | GO:0000904 | P | cell morphogenesis involved in differentiation |  |  |  |  |  |  | --- | --- | --- | --- | 0.0062 | 16 | --- | --- | 0.026 | 16 | 0.031 | 19 |
| 590 | GO:0051640 | P | organelle localization |  |  |  |  |  |  | --- | --- | --- | --- | 0.0063 | 9 | 0.009 | 10 | 0.0053 | 10 | 0.011 | 11 |
| 591 | GO:0031408 | P | oxylipin biosynthetic process |  |  |  |  |  |  | --- | --- | --- | --- | 0.0065 | 11 | 0.0049 | 13 | 0.02 | 11 | 0.0087 | 14 |
| 592 | GO:0015672 | P | monovalent inorganic cation transport |  |  |  |  |  |  | --- | --- | --- | --- | 0.0072 | 23 | --- | --- | --- | --- | 0.027 | 29 |
| 593 | GO:0031365 | P | N-terminal protein amino acid modification |  |  |  |  |  |  | --- | --- | --- | --- | 0.0072 | 6 | --- | --- | 0.0035 | 7 | --- | --- |
| 594 | GO:0006400 | P | tRNA modification |  |  |  |  |  |  | --- | --- | --- | --- | 0.0072 | 6 | --- | --- | 0.016 | 6 | --- | --- |
| 595 | GO:0009875 | P | pollen-pistil interaction |  |  |  |  |  |  | --- | --- | --- | --- | 0.0078 | 12 | 0.00011 | 18 | 0.0039 | 14 | 6.3e-06 | 23 |
| 596 | GO:0016129 | P | phytosteroid biosynthetic process |  |  |  |  |  |  | --- | --- | --- | --- | 0.0078 | 8 | --- | --- | --- | --- | --- | --- |
| 597 | GO:0016132 | P | brassinosteroid biosynthetic process |  |  |  |  |  |  | --- | --- | --- | --- | 0.0078 | 8 | --- | --- | --- | --- | --- | --- |
| 598 | GO:0048438 | P | floral whorl development |  |  |  |  |  |  | --- | --- | --- | --- | 0.0087 | 25 | 0.021 | 28 | --- | --- | 0.002 | 37 |
| 599 | GO:0006270 | P | DNA replication initiation |  |  |  |  |  |  | --- | --- | --- | --- | 0.0095 | 7 | 0.033 | 7 | 0.0016 | 9 | 4.8e-05 | 13 |
| 600 | GO:0009791 | P | post-embryonic development |  |  |  |  |  |  | --- | --- | --- | --- | 0.01 | 87 | 0.046 | 99 | --- | --- | 0.00026 | 138 |
| 601 | GO:0048437 | P | floral organ development |  |  |  |  |  |  | --- | --- | --- | --- | 0.011 | 27 | 0.019 | 31 | 0.044 | 28 | 0.00046 | 43 |
| 602 | GO:0048569 | P | post-embryonic organ development |  |  |  |  |  |  | --- | --- | --- | --- | 0.013 | 27 | 0.022 | 31 | --- | --- | 0.00059 | 43 |
| 603 | GO:0048193 | P | Golgi vesicle transport |  |  |  |  |  |  | --- | --- | --- | --- | 0.014 | 16 | 0.025 | 18 | 0.029 | 17 | 4.2e-05 | 29 |
| 604 | GO:0015991 | P | ATP hydrolysis coupled proton transport |  |  |  |  |  |  | --- | --- | --- | --- | 0.015 | 5 | --- | --- | 0.0062 | 6 | --- | --- |
| 605 | GO:0015988 | P | energy coupled proton transport, against electrochemical gradient |  |  |  |  |  |  | --- | --- | --- | --- | 0.015 | 5 | --- | --- | 0.0062 | 6 | --- | --- |
| 606 | GO:0009644 | P | response to high light intensity |  |  |  |  |  |  | --- | --- | --- | --- | 0.016 | 14 | --- | --- | --- | --- | --- | --- |
| 607 | GO:0045010 | P | actin nucleation |  |  |  |  |  |  | --- | --- | --- | --- | 0.016 | 8 | 0.022 | 9 | 0.04 | 8 | 0.0082 | 11 |
| 608 | GO:0051656 | P | establishment of organelle localization |  |  |  |  |  |  | --- | --- | --- | --- | 0.016 | 7 | 0.0055 | 9 | 0.0032 | 9 | 0.0051 | 10 |
| 609 | GO:0046488 | P | phosphatidylinositol metabolic process |  |  |  |  |  |  | --- | --- | --- | --- | 0.016 | 7 | --- | --- | --- | --- | 0.016 | 9 |
| 610 | GO:0002237 | P | response to molecule of bacterial origin |  |  |  |  |  |  | --- | --- | --- | --- | 0.016 | 7 | --- | --- | --- | --- | 0.0051 | 10 |
| 611 | GO:0040008 | P | regulation of growth |  |  |  |  |  |  | --- | --- | --- | --- | 0.02 | 15 | --- | --- | --- | --- | 0.0028 | 23 |
| 612 | GO:0051667 | P | establishment of plastid localization |  |  |  |  |  |  | --- | --- | --- | --- | 0.02 | 6 | 0.005 | 8 | 0.003 | 8 | 0.013 | 8 |
| 613 | GO:0051644 | P | plastid localization |  |  |  |  |  |  | --- | --- | --- | --- | 0.02 | 6 | 0.005 | 8 | 0.003 | 8 | 0.013 | 8 |
| 614 | GO:0009902 | P | chloroplast relocation |  |  |  |  |  |  | --- | --- | --- | --- | 0.02 | 6 | 0.005 | 8 | 0.003 | 8 | 0.013 | 8 |
| 615 | GO:0010182 | P | sugar mediated signaling pathway |  |  |  |  |  |  | --- | --- | --- | --- | 0.021 | 10 | --- | --- | --- | --- | --- | --- |
| 616 | GO:0009756 | P | carbohydrate mediated signaling |  |  |  |  |  |  | --- | --- | --- | --- | 0.021 | 10 | --- | --- | --- | --- | --- | --- |
| 617 | GO:0009260 | P | ribonucleotide biosynthetic process |  |  |  |  |  |  | --- | --- | --- | --- | 0.021 | 18 | 0.013 | 22 | --- | --- | 0.0049 | 27 |
| 618 | GO:0009165 | P | nucleotide biosynthetic process |  |  |  |  |  |  | --- | --- | --- | --- | 0.024 | 22 | --- | --- | --- | --- | 0.0062 | 33 |
| 619 | GO:0007165 | P | signal transduction |  |  |  |  |  |  | --- | --- | --- | --- | 0.024 | 135 | --- | --- | --- | --- | --- | --- |
| 620 | GO:0016128 | P | phytosteroid metabolic process |  |  |  |  |  |  | --- | --- | --- | --- | 0.024 | 8 | --- | --- | --- | --- | --- | --- |
| 621 | GO:0016131 | P | brassinosteroid metabolic process |  |  |  |  |  |  | --- | --- | --- | --- | 0.024 | 8 | --- | --- | --- | --- | --- | --- |
| 622 | GO:0010015 | P | root morphogenesis |  |  |  |  |  |  | --- | --- | --- | --- | 0.024 | 21 | 0.041 | 24 | --- | --- | 0.015 | 30 |
| 623 | GO:0009063 | P | cellular amino acid catabolic process |  |  |  |  |  |  | --- | --- | --- | --- | 0.025 | 12 | --- | --- | 0.036 | 13 | --- | --- |
| 624 | GO:0009694 | P | jasmonic acid metabolic process |  |  |  |  |  |  | --- | --- | --- | --- | 0.025 | 10 | --- | --- | 0.011 | 12 | --- | --- |
| 625 | GO:0051188 | P | cofactor biosynthetic process |  |  |  |  |  |  | --- | --- | --- | --- | 0.027 | 30 | --- | --- | 0.048 | 33 | --- | --- |
| 626 | GO:0006752 | P | group transfer coenzyme metabolic process |  |  |  |  |  |  | --- | --- | --- | --- | 0.028 | 12 | --- | --- | --- | --- | --- | --- |
| 627 | GO:0007242 | P | intracellular signaling cascade |  |  |  |  |  |  | --- | --- | --- | --- | 0.029 | 79 | --- | --- | --- | --- | --- | --- |
| 628 | GO:0048767 | P | root hair elongation |  |  |  |  |  |  | --- | --- | --- | --- | 0.03 | 9 | --- | --- | --- | --- | --- | --- |
| 629 | GO:0009310 | P | amine catabolic process |  |  |  |  |  |  | --- | --- | --- | --- | 0.032 | 12 | --- | --- | 0.021 | 14 | 0.035 | 16 |
| 630 | GO:0042219 | P | cellular amino acid derivative catabolic process |  |  |  |  |  |  | --- | --- | --- | --- | 0.035 | 6 | --- | --- | 0.0061 | 8 | 0.0082 | 9 |
| 631 | GO:0048513 | P | organ development |  |  |  |  |  |  | --- | --- | --- | --- | 0.036 | 96 | --- | --- | --- | --- | --- | --- |
| 632 | GO:0048731 | P | system development |  |  |  |  |  |  | --- | --- | --- | --- | 0.037 | 96 | --- | --- | --- | --- | --- | --- |
| 633 | GO:0048468 | P | cell development |  |  |  |  |  |  | --- | --- | --- | --- | 0.039 | 30 | --- | --- | --- | --- | --- | --- |
| 634 | GO:0046700 | P | heterocycle catabolic process |  |  |  |  |  |  | --- | --- | --- | --- | 0.045 | 10 | --- | --- | --- | --- | 0.027 | 14 |
| 635 | GO:0016044 | P | cellular membrane organization |  |  |  |  |  |  | --- | --- | --- | --- | 0.046 | 20 | --- | --- | --- | --- | --- | --- |
| 636 | GO:0009642 | P | response to light intensity |  |  |  |  |  |  | --- | --- | --- | --- | 0.047 | 17 | --- | --- | --- | --- | --- | --- |
| 637 | GO:0010374 | P | stomatal complex development |  |  |  |  |  |  | --- | --- | --- | --- | 0.049 | 9 | --- | --- | --- | --- | 0.0087 | 14 |
| 638 | GO:0043566 | F | structure-specific DNA binding |  |  |  |  |  |  | --- | --- | --- | --- | 5e-07 | 23 | 0.00032 | 20 | 0.00019 | 20 | 0.00016 | 24 |
| 639 | GO:0000287 | F | magnesium ion binding |  |  |  |  |  |  | --- | --- | --- | --- | 1.9e-06 | 24 | 1.9e-06 | 27 | 1e-05 | 25 | 3.2e-07 | 33 |
| 640 | GO:0003690 | F | double-stranded DNA binding |  |  |  |  |  |  | --- | --- | --- | --- | 9.2e-06 | 19 | 0.0092 | 15 | 0.00024 | 18 | 0.00033 | 21 |
| 641 | GO:0030170 | F | pyridoxal phosphate binding |  |  |  |  |  |  | --- | --- | --- | --- | 3.5e-05 | 17 | 3.5e-05 | 19 | 2.2e-05 | 19 | 1e-06 | 25 |
| 642 | GO:0070279 | F | vitamin B6 binding |  |  |  |  |  |  | --- | --- | --- | --- | 3.5e-05 | 17 | 3.5e-05 | 19 | 2.2e-05 | 19 | 1e-06 | 25 |
| 643 | GO:0004650 | F | polygalacturonase activity |  |  |  |  |  |  | --- | --- | --- | --- | 5.4e-05 | 23 | --- | --- | 0.0077 | 20 | 0.038 | 22 |
| 644 | GO:0016817 | F | hydrolase activity, acting on acid anhydrides |  |  |  |  |  |  | --- | --- | --- | --- | 0.00014 | 112 | 9e-07 | 144 | 0.026 | 113 | 2.4e-05 | 166 |
| 645 | GO:0017111 | F | nucleoside-triphosphatase activity |  |  |  |  |  |  | --- | --- | --- | --- | 0.00014 | 107 | 3.1e-07 | 140 | 0.0091 | 111 | 2.9e-05 | 158 |
| 646 | GO:0016462 | F | pyrophosphatase activity |  |  |  |  |  |  | --- | --- | --- | --- | 0.00035 | 109 | 2.3e-06 | 141 | 0.027 | 112 | 0.00014 | 160 |
| 647 | GO:0016818 | F | hydrolase activity, acting on acid anhydrides, in phosphorus-containing anhydrides |  |  |  |  |  |  | --- | --- | --- | --- | 0.00043 | 109 | 2e-06 | 142 | 0.032 | 112 | 9.3e-05 | 162 |
| 648 | GO:0004222 | F | metalloendopeptidase activity |  |  |  |  |  |  | --- | --- | --- | --- | 0.0014 | 15 | --- | --- | 0.044 | 13 | --- | --- |
| 649 | GO:0050660 | F | FAD binding |  |  |  |  |  |  | --- | --- | --- | --- | 0.0014 | 20 | 0.0078 | 21 | 0.00011 | 25 | 0.00025 | 29 |
| 650 | GO:0051537 | F | 2 iron, 2 sulfur cluster binding |  |  |  |  |  |  | --- | --- | --- | --- | 0.003 | 6 | --- | --- | --- | --- | --- | --- |
| 651 | GO:0003924 | F | GTPase activity |  |  |  |  |  |  | --- | --- | --- | --- | 0.0035 | 18 | 0.032 | 18 | --- | --- | --- | --- |
| 652 | GO:0005509 | F | calcium ion binding |  |  |  |  |  |  | --- | --- | --- | --- | 0.0051 | 36 | --- | --- | 0.024 | 38 | --- | --- |
| 653 | GO:0008237 | F | metallopeptidase activity |  |  |  |  |  |  | --- | --- | --- | --- | 0.013 | 19 | --- | --- | --- | --- | --- | --- |
| 654 | GO:0008173 | F | RNA methyltransferase activity |  |  |  |  |  |  | --- | --- | --- | --- | 0.016 | 9 | --- | --- | --- | --- | --- | --- |
| 655 | GO:0016409 | F | palmitoyltransferase activity |  |  |  |  |  |  | --- | --- | --- | --- | 0.016 | 5 | 0.045 | 5 | --- | --- | --- | --- |
| 656 | GO:0003684 | F | damaged DNA binding |  |  |  |  |  |  | --- | --- | --- | --- | 0.021 | 10 | --- | --- | --- | --- | --- | --- |
| 657 | GO:0043021 | F | ribonucleoprotein binding |  |  |  |  |  |  | --- | --- | --- | --- | 0.025 | 5 | --- | --- | --- | --- | --- | --- |
| 658 | GO:0004499 | F | flavin-containing monooxygenase activity |  |  |  |  |  |  | --- | --- | --- | --- | 0.038 | 7 | --- | --- | --- | --- | --- | --- |
| 659 | GO:0004372 | F | glycine hydroxymethyltransferase activity |  |  |  |  |  |  | --- | --- | --- | --- | 0.048 | 7 | --- | --- | --- | --- | --- | --- |
| 660 | GO:0044421 | C | extracellular region part |  |  |  |  |  |  | --- | --- | --- | --- | 0.015 | 10 | 0.014 | 11 | 0.043 | 10 | --- | --- |
| 661 | GO:0005874 | C | microtubule |  |  |  |  |  |  | --- | --- | --- | --- | 0.018 | 12 | 0.0004 | 17 | 0.0043 | 15 | 0.0012 | 19 |
| 662 | GO:0005615 | C | extracellular space |  |  |  |  |  |  | --- | --- | --- | --- | 0.018 | 6 | 0.01 | 7 | 0.044 | 6 | 0.042 | 7 |
| 663 | GO:0044454 | C | nuclear chromosome part |  |  |  |  |  |  | --- | --- | --- | --- | 0.018 | 10 | --- | --- | 0.023 | 11 | 0.046 | 12 |
| 664 | GO:0044431 | C | Golgi apparatus part |  |  |  |  |  |  | --- | --- | --- | --- | 0.023 | 23 | --- | --- | 0.017 | 27 | 0.0012 | 37 |
| 665 | GO:0005802 | C | trans-Golgi network |  |  |  |  |  |  | --- | --- | --- | --- | 0.032 | 8 | 0.01 | 10 | --- | --- | 0.0024 | 13 |
| 666 | GO:0018193 | P | peptidyl-amino acid modification |  |  |  |  |  |  | --- | --- | --- | --- | --- | --- | 0.0035 | 14 | --- | --- | --- | --- |
| 667 | GO:0007018 | P | microtubule-based movement |  |  |  |  |  |  | --- | --- | --- | --- | --- | --- | 0.0042 | 16 | --- | --- | 0.022 | 16 |
| 668 | GO:0030244 | P | cellulose biosynthetic process |  |  |  |  |  |  | --- | --- | --- | --- | --- | --- | 0.0055 | 9 | 0.011 | 8 | 0.016 | 9 |
| 669 | GO:0042157 | P | lipoprotein metabolic process |  |  |  |  |  |  | --- | --- | --- | --- | --- | --- | 0.011 | 12 | --- | --- | 0.04 | 12 |
| 670 | GO:0009168 | P | purine ribonucleoside monophosphate biosynthetic process |  |  |  |  |  |  | --- | --- | --- | --- | --- | --- | 0.025 | 7 | --- | --- | 0.018 | 8 |
| 671 | GO:0009167 | P | purine ribonucleoside monophosphate metabolic process |  |  |  |  |  |  | --- | --- | --- | --- | --- | --- | 0.025 | 7 | --- | --- | 0.018 | 8 |
| 672 | GO:0009126 | P | purine nucleoside monophosphate metabolic process |  |  |  |  |  |  | --- | --- | --- | --- | --- | --- | 0.025 | 7 | --- | --- | 0.018 | 8 |
| 673 | GO:0009127 | P | purine nucleoside monophosphate biosynthetic process |  |  |  |  |  |  | --- | --- | --- | --- | --- | --- | 0.025 | 7 | --- | --- | 0.018 | 8 |
| 674 | GO:0006497 | P | protein amino acid lipidation |  |  |  |  |  |  | --- | --- | --- | --- | --- | --- | 0.028 | 11 | --- | --- | --- | --- |
| 675 | GO:0042158 | P | lipoprotein biosynthetic process |  |  |  |  |  |  | --- | --- | --- | --- | --- | --- | 0.028 | 11 | --- | --- | --- | --- |
| 676 | GO:0051130 | P | positive regulation of cellular component organization |  |  |  |  |  |  | --- | --- | --- | --- | --- | --- | 0.033 | 7 | --- | --- | 0.025 | 8 |
| 677 | GO:0006094 | P | gluconeogenesis |  |  |  |  |  |  | --- | --- | --- | --- | --- | --- | 0.041 | 5 | --- | --- | --- | --- |
| 678 | GO:0006984 | P | ER-nuclear signaling pathway |  |  |  |  |  |  | --- | --- | --- | --- | --- | --- | 0.046 | 6 | --- | --- | 0.0024 | 9 |
| 679 | GO:0016668 | F | oxidoreductase activity, acting on sulfur group of donors, NAD or NADP as acceptor |  |  |  |  |  |  | --- | --- | --- | --- | --- | --- | 0.005 | 9 | 0.00082 | 10 | 0.00049 | 12 |
| 680 | GO:0008417 | F | fucosyltransferase activity |  |  |  |  |  |  | --- | --- | --- | --- | --- | --- | 0.018 | 8 | --- | --- | --- | --- |
| 681 | GO:0004003 | F | ATP-dependent DNA helicase activity |  |  |  |  |  |  | --- | --- | --- | --- | --- | --- | 0.023 | 6 | --- | --- | --- | --- |
| 682 | GO:0044451 | C | nucleoplasm part |  |  |  |  |  |  | --- | --- | --- | --- | --- | --- | 0.029 | 33 | --- | --- | 0.042 | 39 |
| 683 | GO:0005654 | C | nucleoplasm |  |  |  |  |  |  | --- | --- | --- | --- | --- | --- | 0.046 | 34 | --- | --- | 0.026 | 43 |
| 684 | GO:0044403 | P | symbiosis, encompassing mutualism through parasitism |  |  |  |  |  |  | --- | --- | --- | --- | --- | --- | --- | --- | 0.0021 | 11 | 0.00057 | 14 |
| 685 | GO:0044419 | P | interspecies interaction between organisms |  |  |  |  |  |  | --- | --- | --- | --- | --- | --- | --- | --- | 0.0021 | 11 | 0.00057 | 14 |
| 686 | GO:0016072 | P | rRNA metabolic process |  |  |  |  |  |  | --- | --- | --- | --- | --- | --- | --- | --- | 0.0038 | 26 | --- | --- |
| 687 | GO:0009658 | P | chloroplast organization |  |  |  |  |  |  | --- | --- | --- | --- | --- | --- | --- | --- | 0.004 | 20 | 0.03 | 21 |
| 688 | GO:0006576 | P | cellular biogenic amine metabolic process |  |  |  |  |  |  | --- | --- | --- | --- | --- | --- | --- | --- | 0.0045 | 18 | 0.0031 | 22 |
| 689 | GO:0006364 | P | rRNA processing |  |  |  |  |  |  | --- | --- | --- | --- | --- | --- | --- | --- | 0.0075 | 25 | --- | --- |
| 690 | GO:0006446 | P | regulation of translational initiation |  |  |  |  |  |  | --- | --- | --- | --- | --- | --- | --- | --- | 0.016 | 6 | 0.046 | 6 |
| 691 | GO:0006338 | P | chromatin remodeling |  |  |  |  |  |  | --- | --- | --- | --- | --- | --- | --- | --- | 0.017 | 9 | --- | --- |
| 692 | GO:0009944 | P | polarity specification of adaxial/abaxial axis |  |  |  |  |  |  | --- | --- | --- | --- | --- | --- | --- | --- | 0.017 | 9 | --- | --- |
| 693 | GO:0042436 | P | indole derivative catabolic process |  |  |  |  |  |  | --- | --- | --- | --- | --- | --- | --- | --- | 0.018 | 5 | 0.046 | 5 |
| 694 | GO:0048439 | P | flower morphogenesis |  |  |  |  |  |  | --- | --- | --- | --- | --- | --- | --- | --- | 0.018 | 5 | --- | --- |
| 695 | GO:0009657 | P | plastid organization |  |  |  |  |  |  | --- | --- | --- | --- | --- | --- | --- | --- | 0.018 | 25 | --- | --- |
| 696 | GO:0009615 | P | response to virus |  |  |  |  |  |  | --- | --- | --- | --- | --- | --- | --- | --- | 0.021 | 15 | 0.042 | 17 |
| 697 | GO:0065001 | P | specification of axis polarity |  |  |  |  |  |  | --- | --- | --- | --- | --- | --- | --- | --- | 0.021 | 9 | --- | --- |
| 698 | GO:0006568 | P | tryptophan metabolic process |  |  |  |  |  |  | --- | --- | --- | --- | --- | --- | --- | --- | 0.031 | 10 | --- | --- |
| 699 | GO:0006586 | P | indolalkylamine metabolic process |  |  |  |  |  |  | --- | --- | --- | --- | --- | --- | --- | --- | 0.031 | 10 | --- | --- |
| 700 | GO:0019375 | P | galactolipid biosynthetic process |  |  |  |  |  |  | --- | --- | --- | --- | --- | --- | --- | --- | 0.031 | 6 | --- | --- |
| 701 | GO:0019374 | P | galactolipid metabolic process |  |  |  |  |  |  | --- | --- | --- | --- | --- | --- | --- | --- | 0.031 | 6 | --- | --- |
| 702 | GO:0009943 | P | adaxial/abaxial axis specification |  |  |  |  |  |  | --- | --- | --- | --- | --- | --- | --- | --- | 0.032 | 9 | --- | --- |
| 703 | GO:0006401 | P | RNA catabolic process |  |  |  |  |  |  | --- | --- | --- | --- | --- | --- | --- | --- | 0.038 | 7 | --- | --- |
| 704 | GO:0006626 | P | protein targeting to mitochondrion |  |  |  |  |  |  | --- | --- | --- | --- | --- | --- | --- | --- | 0.04 | 8 | 0.022 | 10 |
| 705 | GO:0070585 | P | protein localization in mitochondrion |  |  |  |  |  |  | --- | --- | --- | --- | --- | --- | --- | --- | 0.04 | 8 | 0.022 | 10 |
| 706 | GO:0010050 | P | vegetative phase change |  |  |  |  |  |  | --- | --- | --- | --- | --- | --- | --- | --- | 0.042 | 6 | 0.013 | 8 |
| 707 | GO:0006402 | P | mRNA catabolic process |  |  |  |  |  |  | --- | --- | --- | --- | --- | --- | --- | --- | 0.042 | 6 | --- | --- |
| 708 | GO:0008380 | P | RNA splicing |  |  |  |  |  |  | --- | --- | --- | --- | --- | --- | --- | --- | 0.042 | 23 | 0.0028 | 33 |
| 709 | GO:0006013 | P | mannose metabolic process |  |  |  |  |  |  | --- | --- | --- | --- | --- | --- | --- | --- | 0.042 | 5 | --- | --- |
| 710 | GO:0022613 | P | ribonucleoprotein complex biogenesis |  |  |  |  |  |  | --- | --- | --- | --- | --- | --- | --- | --- | 0.047 | 41 | --- | --- |
| 711 | GO:0031324 | P | negative regulation of cellular metabolic process |  |  |  |  |  |  | --- | --- | --- | --- | --- | --- | --- | --- | 0.049 | 24 | --- | --- |
| 712 | GO:0016829 | F | lyase activity |  |  |  |  |  |  | --- | --- | --- | --- | --- | --- | --- | --- | 0.017 | 66 | --- | --- |
| 713 | GO:0005852 | C | eukaryotic translation initiation factor 3 complex |  |  |  |  |  |  | --- | --- | --- | --- | --- | --- | --- | --- | 0.044 | 7 | 0.046 | 8 |
| 714 | GO:0006397 | P | mRNA processing |  |  |  |  |  |  | --- | --- | --- | --- | --- | --- | --- | --- | --- | --- | 0.00057 | 36 |
| 715 | GO:0000398 | P | nuclear mRNA splicing, via spliceosome |  |  |  |  |  |  | --- | --- | --- | --- | --- | --- | --- | --- | --- | --- | 0.0044 | 22 |
| 716 | GO:0000375 | P | RNA splicing, via transesterification reactions |  |  |  |  |  |  | --- | --- | --- | --- | --- | --- | --- | --- | --- | --- | 0.0094 | 22 |
| 717 | GO:0000377 | P | RNA splicing, via transesterification reactions with bulged adenosine as nucleophile |  |  |  |  |  |  | --- | --- | --- | --- | --- | --- | --- | --- | --- | --- | 0.0094 | 22 |
| 718 | GO:0006099 | P | tricarboxylic acid cycle |  |  |  |  |  |  | --- | --- | --- | --- | --- | --- | --- | --- | --- | --- | 0.011 | 11 |
| 719 | GO:0046356 | P | acetyl-CoA catabolic process |  |  |  |  |  |  | --- | --- | --- | --- | --- | --- | --- | --- | --- | --- | 0.011 | 11 |
| 720 | GO:0009887 | P | organ morphogenesis |  |  |  |  |  |  | --- | --- | --- | --- | --- | --- | --- | --- | --- | --- | 0.016 | 52 |
| 721 | GO:0048645 | P | organ formation |  |  |  |  |  |  | --- | --- | --- | --- | --- | --- | --- | --- | --- | --- | 0.016 | 16 |
| 722 | GO:0010103 | P | stomatal complex morphogenesis |  |  |  |  |  |  | --- | --- | --- | --- | --- | --- | --- | --- | --- | --- | 0.017 | 10 |
| 723 | GO:0009060 | P | aerobic respiration |  |  |  |  |  |  | --- | --- | --- | --- | --- | --- | --- | --- | --- | --- | 0.018 | 12 |
| 724 | GO:0030968 | P | endoplasmic reticulum unfolded protein response |  |  |  |  |  |  | --- | --- | --- | --- | --- | --- | --- | --- | --- | --- | 0.02 | 7 |
| 725 | GO:0006826 | P | iron ion transport |  |  |  |  |  |  | --- | --- | --- | --- | --- | --- | --- | --- | --- | --- | 0.021 | 9 |
| 726 | GO:0010016 | P | shoot morphogenesis |  |  |  |  |  |  | --- | --- | --- | --- | --- | --- | --- | --- | --- | --- | 0.021 | 44 |
| 727 | GO:0000087 | P | M phase of mitotic cell cycle |  |  |  |  |  |  | --- | --- | --- | --- | --- | --- | --- | --- | --- | --- | 0.022 | 11 |
| 728 | GO:0080010 | P | regulation of oxygen and reactive oxygen species metabolic process |  |  |  |  |  |  | --- | --- | --- | --- | --- | --- | --- | --- | --- | --- | 0.022 | 11 |
| 729 | GO:0007067 | P | mitosis |  |  |  |  |  |  | --- | --- | --- | --- | --- | --- | --- | --- | --- | --- | 0.022 | 11 |
| 730 | GO:0015698 | P | inorganic anion transport |  |  |  |  |  |  | --- | --- | --- | --- | --- | --- | --- | --- | --- | --- | 0.022 | 14 |
| 731 | GO:0015996 | P | chlorophyll catabolic process |  |  |  |  |  |  | --- | --- | --- | --- | --- | --- | --- | --- | --- | --- | 0.025 | 8 |
| 732 | GO:0046149 | P | pigment catabolic process |  |  |  |  |  |  | --- | --- | --- | --- | --- | --- | --- | --- | --- | --- | 0.025 | 8 |
| 733 | GO:0007034 | P | vacuolar transport |  |  |  |  |  |  | --- | --- | --- | --- | --- | --- | --- | --- | --- | --- | 0.032 | 12 |
| 734 | GO:0009913 | P | epidermal cell differentiation |  |  |  |  |  |  | --- | --- | --- | --- | --- | --- | --- | --- | --- | --- | 0.033 | 35 |
| 735 | GO:0046148 | P | pigment biosynthetic process |  |  |  |  |  |  | --- | --- | --- | --- | --- | --- | --- | --- | --- | --- | 0.035 | 28 |
| 736 | GO:0008544 | P | epidermis development |  |  |  |  |  |  | --- | --- | --- | --- | --- | --- | --- | --- | --- | --- | 0.039 | 35 |
| 737 | GO:0007398 | P | ectoderm development |  |  |  |  |  |  | --- | --- | --- | --- | --- | --- | --- | --- | --- | --- | 0.039 | 35 |
| 738 | GO:0006986 | P | response to unfolded protein |  |  |  |  |  |  | --- | --- | --- | --- | --- | --- | --- | --- | --- | --- | 0.041 | 7 |
| 739 | GO:0034620 | P | cellular response to unfolded protein |  |  |  |  |  |  | --- | --- | --- | --- | --- | --- | --- | --- | --- | --- | 0.041 | 7 |
| 740 | GO:0009109 | P | coenzyme catabolic process |  |  |  |  |  |  | --- | --- | --- | --- | --- | --- | --- | --- | --- | --- | 0.043 | 11 |
| 741 | GO:0060255 | P | regulation of macromolecule metabolic process |  |  |  |  |  |  | --- | --- | --- | --- | --- | --- | --- | --- | --- | --- | 0.044 | 314 |
| 742 | GO:0032507 | P | maintenance of protein location in cell |  |  |  |  |  |  | --- | --- | --- | --- | --- | --- | --- | --- | --- | --- | 0.045 | 8 |
| 743 | GO:0008283 | P | cell proliferation |  |  |  |  |  |  | --- | --- | --- | --- | --- | --- | --- | --- | --- | --- | 0.046 | 19 |
| 744 | GO:0009595 | P | detection of biotic stimulus |  |  |  |  |  |  | --- | --- | --- | --- | --- | --- | --- | --- | --- | --- | 0.046 | 5 |
| 745 | GO:0016444 | P | somatic cell DNA recombination |  |  |  |  |  |  | --- | --- | --- | --- | --- | --- | --- | --- | --- | --- | 0.046 | 5 |
| 746 | GO:0033013 | P | tetrapyrrole metabolic process |  |  |  |  |  |  | --- | --- | --- | --- | --- | --- | --- | --- | --- | --- | 0.048 | 24 |
| 747 | GO:0005789 | C | endoplasmic reticulum membrane |  |  |  |  |  |  | --- | --- | --- | --- | --- | --- | --- | --- | --- | --- | 0.034 | 21 |
| 748 | GO:0000139 | C | Golgi membrane |  |  |  |  |  |  | --- | --- | --- | --- | --- | --- | --- | --- | --- | --- | 0.039 | 18 |
| 749 | GO:0042175 | C | nuclear envelope-endoplasmic reticulum network |  |  |  |  |  |  | --- | --- | --- | --- | --- | --- | --- | --- | --- | --- | 0.042 | 21 |
| 750 | GO:0016469 | C | proton-transporting two-sector ATPase complex |  |  |  |  |  |  | --- | --- | --- | --- | --- | --- | --- | --- | --- | --- | 0.046 | 12 |

Capture

Drag to outliner or Upload

Close

Ok, done!

PrivateGroup

SaveCancel

+Share to a new group

- +Share to a new group

PostCancel

PostCancel
