# Supplementary material for: Refining bulk segregant analyses: ontology-mediated discovery of flowering time genes in Brassica oleracea
Source: Plant Methods. 2022 Jul 4;18:92. doi: 10.1186/s13007-022-00921-y (PMC9252076; doi:10.1186/s13007-022-00921-y)
Supplement: Supplementary file 6 — Additional file 6: Methods. [file 13007_2022_921_MOESM6_ESM.pdf]

SUPPLEMENTARY MATERIALS

Supplements referenced in main manuscript and included in this archive

`st1_histogram_flowering_time_Brassica_F1.xlsx`
Supplementary Table 1: flowering time per specimen, data for Figure 1

`st2_bulk_sequencing_yields.docx`
Supplementary Table 2: Raw yields of bulk sequencing

`st3_seacompare.html`
Supplementary Table 3: Results of the SEACompare analysis

`st4_snpeff.xlsx`
Supplementary Table 4: Results of the SnpEff analysis

`st5_seqstats_genome.xlsx`
Supplementary Table 5: Results of Jersey Kale genome sequencing

Supplementary analysis code and data

To allow for verification of the analyses we performed and reuse of the workflow presented here, we have bundled all scripts, library code, and 3rd party software in a Docker image, which is available from the Docker hub as `naturalis/brassica-snps`. The scripts that are referenced in the following sections are contained within that image and are also available from the project's git repository (note that this repo also contains experimental code not relevant for the present analyses).

The data files that these scripts operate on should be organized in a directory structure whose root folder is referred to as `${DATA}`. In the subsequent sections we detail where the supplementary data archives are deposited and how they should be stored in this structure.

SUPPLEMENTARY METHODS

Genome assembly

The processed reference genome (sans scaffolds; chromosomes only) is available from the main data submission of this study, deposited at Zenodo as `10.5281/zenodo.3402201`. The processed FASTA file, and the annotation (in GFF3 format), are contained within the `reference.zip` archive, and should be stored locally as:

`${DATA}/reference/Brassica_oleracea.v2.1.dna.toplevel.chromosomes.fa`
Reference genome as FASTA, restricted to only the chromosomes, no scaffolds

`${DATA}/reference/Brassica_oleracea.v2.1.39.chr.gff3.gz`
Reference genome annotations as GFF3

Reference mapping was automated with `bwa.sh`. Input files are deposited at the SRA as project PRJNA564368, containing the BioSamples group-1-EF, group-2-IF, group-3-LF, group-4 (which is the Jersey Kale genome) and group-5-NF, hereafter referred to as `${SAMPLE}`. These should be placed in a local folder structure as follows:

`${DATA}/BSA/${SAMPLE}/${SAMPLE}_R1.fastq.gz`
Paired end 1, FASTQ

`${DATA}/BSA/${SAMPLE}/${SAMPLE}_R2.fastq.gz`
Paired end 2, FASTQ

SNP calling was automated with `snp.sh`. The resulting alignments (sorted BAM files) and variants (compressed VCF files) are available from Zenodo under the following locations:

- **EF**: 10.5281/zenodo.3401590
- **IF**: 10.5281/zenodo.3401703
- **LF**: 10.5281/zenodo.3401731
- **NF**: 10.5281/zenodo.3402013
- **Genome**: 10.5281/zenodo.5211308

In each case, the files in these Zenodo submissions should be organized locally as follows:

`${DATA}/BSA/${SAMPLE}/${SAMPLE}_pe.sorted.bam`
Genome alignment, BAM

`${DATA}/BSA/${SAMPLE}/${SAMPLE}_pe.sorted.bam.RG.vcf.gz`
Variant call file, VCF

Bulk genotyping and QTL region analysis

The steps described here operate in parallel on all contrasts. The files produced are made available as the archive `contrasts.zip` in the main data supplement. In it, there are subfolders for the contrasts, named `EF-IF`, `EF-LF`, `EF-NF`, `IF-LF`, `IF-NF` and `LF-NF`. The files inside these subfolders (i.e. `${DATA}/contrasts/${CONTRAST}/*`) all follow the same naming scheme, which is described further in the following sections.

Joint genotyping - An important issue that needs to be taken care of is that the BAM data (and subsequently the VCF data) that go into the joint genotyping are properly annotated by "read group" (`@RG`), which is the field in SAM/BAM files that is used for identifiers of samples. This so that when `CombineGVCFs` merges the VCF files for the two bulks this is done in such a way that the variants can be traced back to the bulk in which they were observed once `GenotypeGVCFs` does its thing. This is why the extra `-R` argument with a read group ID was passed into `BWA-MEM` when doing the assembly. With that properly taken care of, the GATK `CombineGVCFs/GenotypeGVCFs` workflow was then automated using `genotype.pl`. The files produced by these steps are named:

`${DATA}/contrasts/${CONTRAST}/combined-snps-${CONTRAST}.vcf.gz`
Output from VCF merge

`${DATA}/contrasts/${CONTRAST}/joint-genotypes-${CONTRAST}.vcf.gz`
Results from joint genotyping

QTL analysis - To get the data into R we needed to transform the GVCF files that GATK produces into tab-separated tables, which we did using `qtlseqr.sh`. The actual calculations performed in R are shown in the script `QTLseqr.R`. The files produced by these steps are named:

`${DATA}/contrasts/${CONTRAST}/SNPs_from_GATK-${CONTRAST}.table`
SNPs as TSV

`${DATA}/contrasts/${CONTRAST}/SNPs-gprime2.5-${CONTRAST}.csv`
SNPs after filtering

`${DATA}/contrasts/${CONTRAST}/gprime.png`
Visualization of QTL regions

`${DATA}/contrasts/${CONTRAST}/QTL-regions-${CONTRAST}.csv`
QTL region coordinates

Database construction - To be able to reconcile the observed SNPs with gene coordinates, we created a relational database with the SQLite schema `snps.sql`, into which we imported data tables for chromosomes (names, centromere locations), genomic features (i.e. locations of genes, 3'/5' UTRs, exons, CDSs, from the TO1000 reference genome annotation), linkage maps (markers, fwd/rev primer sequences, locations in cM) and SNPs (locations, ref/alt allele, numerous summary statistics including G' values).

From the database schema we then generated an object-relational API (the Perl classes within the `lib` folder structure) using `make_dbix_api.sh` to provide programmable access to the integrated, indexed data such that downstream analysis scripts have simplified, quicker query access. The following files went into populating the database (see `sqlite.zip`):

`${DATA}/sqlite/chromosomes.tsv`
Created manually from GFF3

`${DATA}/sqlite/features.tsv`
Created from GFF3 using `make_features.pl`

`${DATA}/sqlite/qtl_regions.tsv`

Created from `*/QTL-regions-${CONTRAST}.csv` using `make_qtl_regions.pl`

`${DATA}/sqlite/snps.tsv`

Created from `*/SNPs-gprime2.5-${CONTRAST}.csv` using `make_snps.pl`

`${DATA}/sqlite/linkages.tsv`

Explained in the Linkages section (Note - the linkages are not directly relevant to the present paper, but we might need them later for genotyping in other experiments.)

Finding QTL genes with nonsynonymous substitutions - For each contrast, we then queried which genes intersect with the inferred QTL regions using `genes_in_qtl_regions.pl`, creating the list `genes.txt` with 14257 *B. oleracea* gene IDs sorted by the number of contrasts in which they appear in a QTL region. For each of these genes, we then calculated using `snps_in_cds.pl` whether they contain non-synonymous SNPs in their coding regions, which we recorded in `snps.tsv`. Then, for each contrast, we extracted the gene identifiers that contain nonsynonymous SNPs and translated these to UniProtKB/TrEMBL identifiers using `biomart.pl`, resulting in files named as:

`${DATA}/contrasts/${CONTRAST}/uniprot.txt`

UniProtKB/TrEMBL IDs

Functional enrichment, refinement, and pathway analysis

Enrichment analyses - The individual analyses were run on the agriGO web service 1.0, which has since gone out of service. However, the v2.0 implements the same core methods, just for more species and with some more additional data exploration. We saved the following output for each contrast:

`${DATA}/contrasts/${CONTRAST}/enriched.png`

Subgraph of enriched GO terms and ancestors

`${DATA}/contrasts/${CONTRAST}/enriched.tsv`

Tabel with GO terms, SEA statistics and UniProt IDs

We then forwarded the results of the SEAs to the cross comparison provided by SEACOMPARE. The result of this analysis (`st3_seacompare.html`) identifies the contrasts only by their internal job ID, which corresponds with the contrasts like so:

- **EF-IF**: 383155352
- **EF-LF**: 283547849
- **EF-NF**: 847435652
- **IF-LF**: 322714491
- **IF-NF**: 933274933
- **LF-NF**: 577207982

To assess whether terms returned by AgriGO are children of GO:0003006, we traversed the GO using the data release of 2018-09-13 (downloaded from

http://snapshot.geneontology.org/ontology/go-basic.obo in OBO format version 1.2). We did this using `go_filter.pl`. This resulted in the following file for each contrast:

`${DATA}/contrasts/${CONTRAST}/enriched_GO_0003006.tsv`
Pruned version of */`enriched.tsv`

The pruned files from the previous step became the input files for generating Figure 3 using the `go_treebuilder.pl` script and GraphViz's `dot` program.

Figure 2 was generated using Circos configured using a folder structure maintained on the project's git repository. The coordinates for chromosomes and centromeres (i.e. the karyotype track) reflect the values as per the public release version of the TO1000DH3 reference genome, i.e. v2.1.39 of EnsemblPlants. The coordinates of flowering time gene copies were kindly provided by Sarah-Veronica Schießl-Weidenweber. The heat map tracks for the G' values are in windows of size 10^6 and were generated using `circos_make_gprime.pl` and the SQLite database.

Figure 4 is the prettified result of a g:Profiler pathway analysis using the reduced gene set contained in the `*/enriched_GO_0003006.tsv` files. To create a publication-ready vector graphic we used the spreadsheet output of the web tool and processed this with `gprofiler_pp.pl`

SnpEff analysis of variant impacts

The confirmatory analysis using SnpEff to assess the distribution and impact of SNPs in genes involved in flowering time was performed as a separate complement to the approach developed in the present manuscript. The analysis is recorded in a git repository identified by 10.5281/zenodo.5211461
